# Overcoming EGFR Resistance by Monovalent and Bident Inhibitors Targeting Cys775

**DOI:** 10.1021/acscentsci.6c01219

**Published:** 2026-09-29

**Authors:** Zhengnian Li, Jie Jiang, Yaning Wang, Scott B. Ficarro, Stephen J. Collins, Tyler S. Beyett, Ilse K. Schaeffner, Isidoro Tavares, Felix H. Gottlieb, Dhiraj Suda, Prafulla C. Gokhale, Leah M. Black-Holmes, Dimitris Gazgalis, Michael J. Eck, Pasi A. Jänne, Jarrod A. Marto, Jianwei Che, Nathanael S. Gray, Tinghu Zhang

**Affiliations:** † Department of Chemical and Systems Biology, Stanford University, Stanford, California 94305, United States; ‡ Stanford Cancer Institute, ChEM-H, 6429 Stanford University, Stanford, California 94305, United States; § Lowe Center for Thoracic Oncology and Department of Medical Oncology, Dana−Farber Cancer Institute, Boston, Massachusetts 02215, United States; ∥ Department of Medicine, Harvard Medical School, Boston, Massachusetts 02215, United States; ⊥ Department of Cancer Biology, Dana−Farber Cancer Institute, Boston, Massachusetts 02215, United States; # Department of Pathology, Brigham and Women’s Hospital and Harvard Medical School, Boston, Massachusetts 02115, United States; △ Department of Biological Chemistry and Molecular Pharmacology, Harvard Medical School, Boston, Massachusetts 02115, United States; ○ Experimental Therapeutics Core and Robert and Renée Belfer Center for Applied Cancer Science, Dana−Farber Cancer Institute, Boston, Massachusetts 02215, United States; ◆ Department of Biochemistry & Molecular Genetics, University of Virginia School of Medicine, Department of Chemistry, University of Virginia College and Graduate School of Arts & Sciences, Charlottesville, Virginia 22904, United States

## Abstract

Covalent targeting of EGFR cysteine 797 by osimertinib is one of the most successful breakthroughs in targeted therapy, fundamentally transforming the treatment landscape for non-small-cell lung cancer (NSCLC) patients. However, resistance driven by the mutation of C797 remains a major clinical challenge. Developing novel covalent strategies beyond C797 targeting presents a compelling opportunity for next-generation EGFR inhibitors. We first demonstrated that cysteine 775, located deep within the ATP-binding pocket, is accessible by a rationally designed covalent molecule ZNL-3, which as the first-in-class covalent cysteine 775 inhibitor exhibited strong efficacy in osimertinib-resistant mouse models. To further enhance resilience to resistance-causing mutations, we developed a dual-warhead, bident compound YNW-1 which covalently targets both cysteines 775 and 797 simultaneously. YNW-1 is the first intramolecular lock to exhibit balanced reactive efficiency on both cysteines, rendering single-site mutations ineffective at conferring resistance. This study establishes the therapeutic potential of an EGFR covalent inhibitor through unprecedented targeting of cysteine 775. It also provides the first evidence that dual-cysteine engagement offers superior efficacy to conventional covalent inhibitors by delaying the emergence of resistance. Further optimization for clinical translation is currently ongoing in our laboratory.

## Graphical abstract

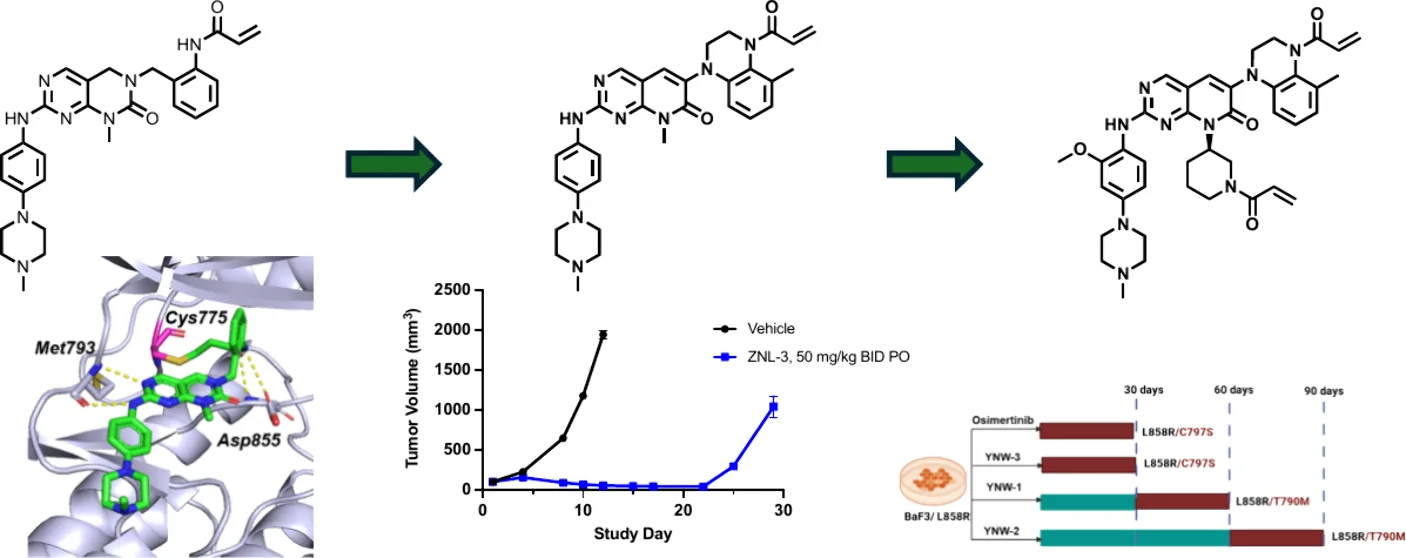

## Introduction

Mutations in the epidermal growth factor receptor (EGFR) gene are major drivers in non-small cell lung cancer (NSCLC), which accounts for over 80% of all lung cancer cases.^1,2^ Targeting EGFR with covalent small-molecule inhibitors has been one of the most successful strategies in precision oncology. Osimertinib, a third-generation covalent EGFR inhibitor, selectively modifies cysteine 797 (C797) and has demonstrated superior potency and efficacy compared to first-generation reversible inhibitors such as gefitinib and erlotinib, which target EGFR mutations like L858R.^3^ Its broad activity across a range of EGFR mutations is largely attributed to its covalent binding mechanism, which confers prolonged target engagement and enhanced pharmacodynamic effects.^4–6^ However, resistance to osimertinib inevitably emerges through diverse mechanisms. In the first-line setting, acquired EGFR mutations occur in approximately 10% of patients at progression, including ∼6% with C797S, whereas EGFR-independent mechanisms such as MET amplification (∼16%) are more frequent.^7^ Among EGFR-dependent mechanisms, the C797S mutation, which abolishes the covalent binding site, is the most prevalent and represents a major unmet need.^8–10^ Although several fourth-generation strategies have been pursued, including allosteric inhibitors, PROTAC degraders, and reversible C797S-active inhibitors,^11–15^ each approach faces limitations in mutation coverage, druglike properties, or kinase selectivity, and convincing clinical progress remains limited.

To address resistance driven by the loss of C797 engagement, we explored an alternative nucleophilic residue within the EGFR protein that could serve as an additional covalent anchoring point. This led to the discovery of cysteine 775 (C775), located in the back pocket of the active site.^16^ Compared to C797, C775 is more deeply buried and spatially constrained and less nucleophilic due to the lack of a polarizing environment that makes it challenging to target but also offers the potential for highly selective covalent inhibition as it is not highly conserved in the kinome.^16^ Our prior study demonstrated that C775 is targetable through a bidentate design (ZNL-0056) linking C797 and C775, offering an initial proof-of-concept, although the resulting inhibitor remained largely dependent on bonding with C797.^16^

In this study, we pursued a reverse design strategy, beginning with optimization of C775. This approach led to the identification of ZNL-3, a potent mutant EGFR inhibitor that is primarily driven by covalency with C775. Building upon this scaffold, we further developed a bidentate compound YNW-1, which is mechanistically distinct from ZNL-0056 and exhibits equal efficiency on both C797 and C775, offering enhanced resilience to mutation-driven resistance. Furthermore, we demonstrated that the single-warhead compound ZNL-3 exhibits high oral dosing efficacy in a xenograft mouse model harboring the L858R/C797S EGFR mutation, highlighting its promising therapeutic potential for patients who progress on osimertinib treatment. For the bident compound YNW-1, in vitro resistance studies showed a lower frequency of resistance-associated mutations at either covalent engagement site, compared with osimertinib, suggesting that dual covalent engagement may increase the genetic barrier to on-target, EGFR-mutation-mediated resistance.

## Results

### Evolution of Designs on Cysteine775-Targeting Covalent Inhibitor

The wealth of publicly accessible co-crystal structures of EGFR provides the opportunity for structure-guided design of many potential C775 targeted covalent inhibitors. We previously reported a two-step approach utilizing a benzimidazole scaffold in which initial reaction with C797 would tether the compound to the ATP-binding site, thereby achieving sufficient induced proximity for slower covalent bond formation to the less-reactive C775 residue. While this strategy successfully delivered the first dual covalent C775/C797 inhibitor, ZNL-0056, it renders the compound highly susceptible to resistance through the mutation of C797. The question of whether targeting C775 itself is a viable strategy for achieving a desired pharmacology remains to be addressed. We suspected that poor accessibility and lower reactivity of C775 relative to C797 might contribute to the challenge and, indeed, C775 has not scored as a ligandable cysteine in any reported covalent fragment screening chemoproteomics studies.^17^ We therefore decided to explore the reverse approach of designing compounds that would target C775 preferentially over C797. Using available liganded EGFR co-structures, we designed and synthesized a variety of molecules bearing electrophiles that were predicted to project toward C775. We developed a mass spectrometry based protein labeling assay with Cys797Ser mutant EGFR to enable detection of even weak covalent binders. Although many designs were unsuccessful, a focused medicinal chemistry campaign based on a dihydropyrimido­[4,5-*d*]­pyrimidin-4-one scaffold led to the identification of a promising hit compound, ZNL-1 (Figure 1A). Among over 50 designed analogues, ZNL-1 demonstrated robust target engagement, binding over 90% of the EGFR^L858R^ mutant in mass spectrometry analysis (Figure 1B). ZNL-1 inhibited EGFR kinase activity in a homogeneous time-resolved fluorescence (HTRF) kinase assay with an apparent IC_50_ of 17.6 nM, representing a 16-fold less potent than Osimertinib (Figure 1C). Because the potency of irreversible covalent inhibitors is typically time-dependent and not always adequately described by equilibrium IC_50_ measurements, instead of a precise measurement of *K*_inact_/*K*_I_, we use the term “apparent IC_50_” throughout this study to denote inhibitory potency measured under the defined assay conditions which allows comparison across compounds consistently. To enable structure-based optimization, we solved the co-crystal structure of ZNL-1 bond to EGFR. As shown in Figure 1D, ZNL-1 forms the expected two hydrogen bonds with methionine 793 in the hinge through 2-aminopyrimidine moiety and the methylated piperazine was exposed to the solvent region. The benzyl group that possesses an acrylamide warhead adopted a vertical pose to the urea ring and was well-accommodated into the hydrophobic back pocket, with the acrylamide warhead forming a covalent bond to C775. The amide group adopts a non-canonical cis configuration to form two hydrogen bonds with aspartic acid 855 and the methyl group on second N of urea reaches to the sugar pocket (Figure S1 and Table S1). Given that covalent bond formation is a typically slow step and requires proper geometry to form transition state could improve the kinetics of nucleophilic reaction with thiol group of cysteine. To test this hypothesis, we have systematically modified ZNL-1 to improve reversible interactions and the warhead orientation toward C775, leading to more than 100 designed analogues. Among this series, ZNL-2 featured a cyclized ring bridging the benzyl group and the acrylamide NH that proved to be the most effective in enhancing potency, exhibiting an apparent biochemical IC_50_ of 0.49 nM, while maintaining high labeling efficiency (67%) on L858R EGFR (Figure 1A and 1B). Next, a stereoselective synthetic route was developed to enable the synthesis of the enantiomeric pure compounds, ZNL-2S and ZNL-2R. The S enantiomer exhibited approximately 40-fold lower apparent IC_50_ in the HTRF kinase assay, compared to the R enantiomer (Figure 1C). Further optimization on the core heterocycle resulted in the discovery of ZNL-3 which is with pyrido­[2,3-*d*] pyrimidin-7­(8*H*)-one scaffold and equipped with methyl on phenyl group which was expected to improve compound stability and to modulate the warhead reactivity (Figure 1A). Labeling by intact MS was >90% with single digit nanomolar apparent IC_50_ in HTRF assay respectively (Figure 1B and 1C), while the reversible counterpart ZNL-3r lost significant potency (∼79-fold). More importantly, ZNL-3 demonstrated markedly improved metabolic stability in both hepatocyte and whole blood assay. In the mouse whole blood, the half-life (*T*_1/2_) of ZNL-3 increased to 205 min, a more than 16-fold improvement over ZNL-2, which displayed a *T*_1/2_ of only 12.4 min (Table S2).

**Figure 1:**
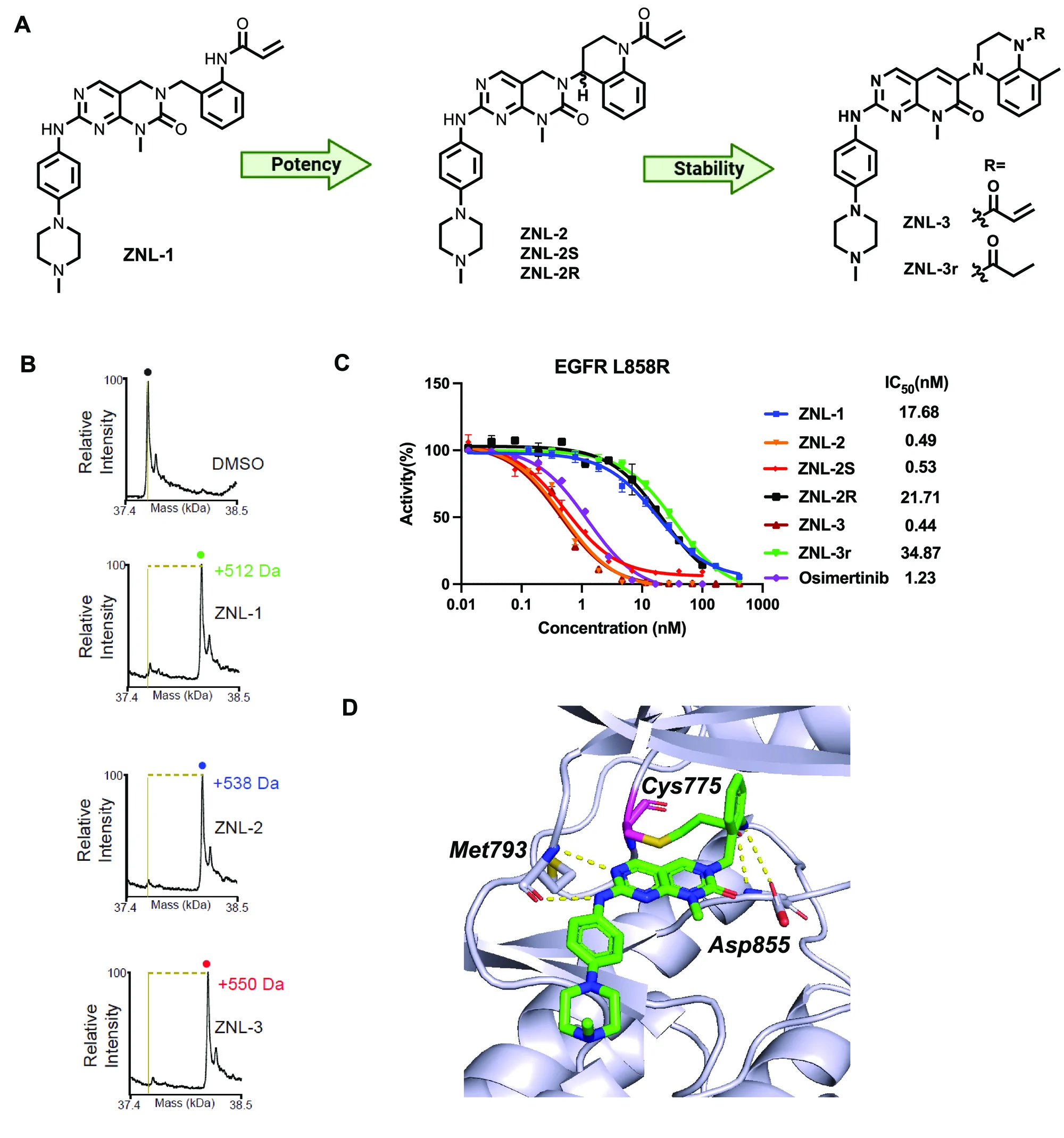
Evolution of designs on cysteine775-targeting covalent inhibitor. (A) Compound structures for EGFR cysteine-targeting molecules. (B) Intact protein mass spectrometry analysis of EGFR L858R treated with indicated compound or DMSO. (C) Biochemical IC_50_s for the indicated compounds against EGFR L858R. Data are presented as the mean ± SEM. Representative data from three independent experiments are shown. (D) Cocrystal structure of ZNL-1 with WT EGFR.

### ZNL-3 Covalently Inhibits EGFR Cys797-Ser Mutation and Is Efficacious in L858R/C797S Xenograft Mouse Model

EGFR-dependent Ba/F3 cells are a well-established and widely used platform in EGFR-targeted drug discovery. Accordingly, we employed three engineered Ba/F3 cells carrying single or double EGFR mutations—L858R, L858R/C797S, and L858R/C775S—to evaluate the anti-proliferation activity of ZNL-3. At 72-hour timepoint, ZNL-3 inhibited cell growth with an IC_50_ below 1 nM in L858R Ba/F3 cells, comparable to Osimertinib, whereas negative control ZNL-3r remarkably lost this activity (Figure 2A), supporting a covalent mechanism. Notably, mutation of cysteine 797 to a serine, ZNL-3 maintained its potency, while Osimertinib lost nearly 1000-fold activity (Figure 2B). The mutation of C775 to serine resulted in an opposite observation, and the activity for ZNL-3 significantly diminished, reminiscent of a covalent dependency as reflected by ZNL-3r (Figure 2C). To examine target engagement, two biotin probes conjugating osimertinib or ZNL-0013,^16^ which can covalently label on cysteine 797 and cysteine 775, respectively, in cell lysate were used to quantify the unmodified EGFR protein under pre-treatment of ZNL-3 in Ba/F3 cells. As exhibited in Figure 2D and Figure S2A, ZNL-3 achieved nearly complete engagement of EGFR at a concentration of 50 nM in both L858R and L858R/C797S cells. As an orthogonal assay, measuring downstream signaling on p-EGFR, p-ERK, and p-AKT collectively highlighted on-target effects with a starting concentration from 10 to 50 nM (Figure 2E and Figure S2B).

**Figure 2:**
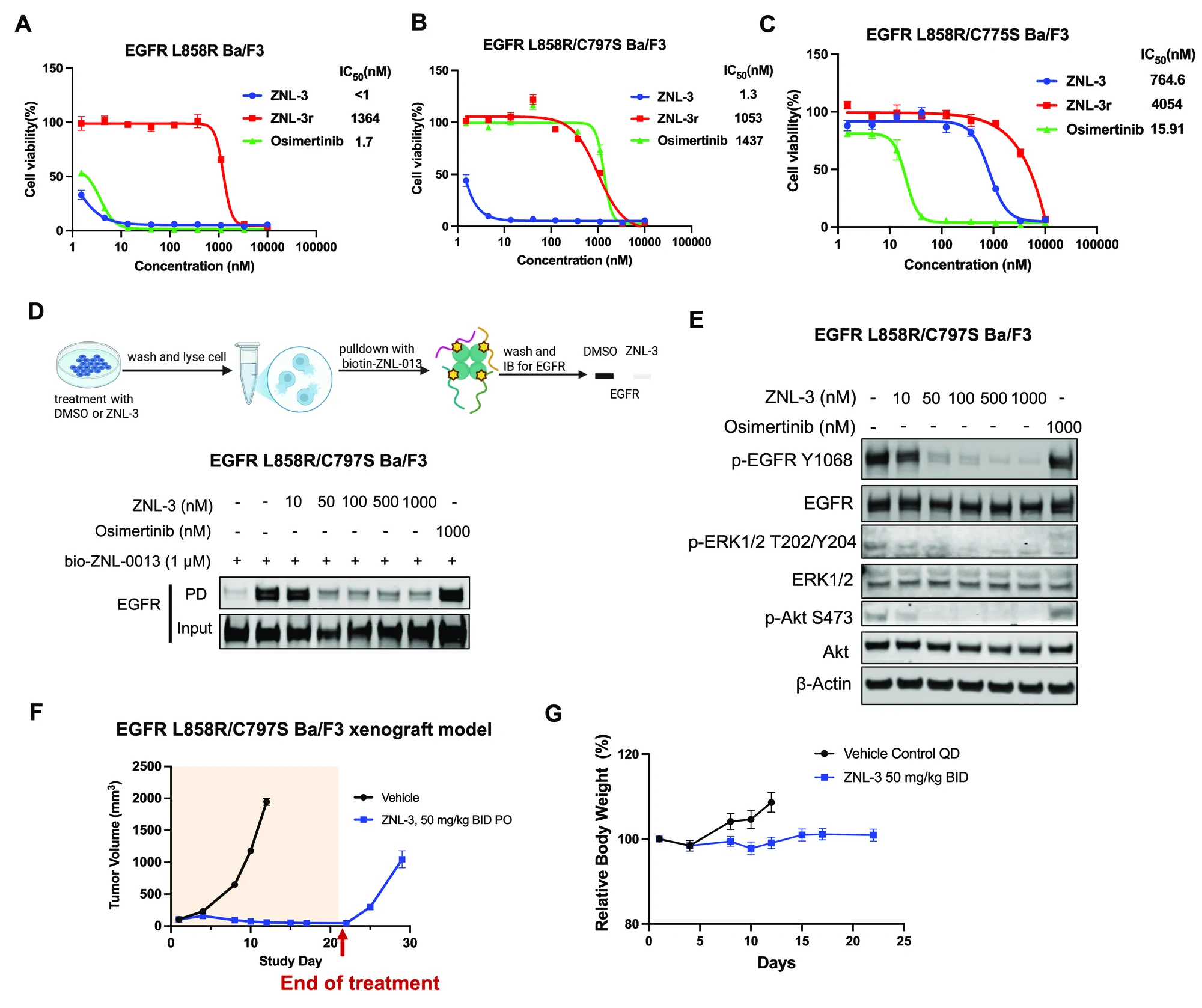
ZNL-3 covalently inhibits EGFR Cys797-Ser mutation and is efficacious in L858R/C797S xenograft mouse model. (A) Dose-response curves for ZNL-3, ZNL-3r and osimertinib in EGFR L858R Ba/F3 cells following 72 h of treatment. Cell viability was assessed with CellTiter-Glo. Data are presented as the mean ± SEM. Representative data from three independent experiments are shown. (B) Dose-response curves for ZNL-3, ZNL-3r, and osimertinib in EGFR L858R/C797S Ba/F3 cells following 72 h of treatment. Cell viability was assessed with CellTiter-Glo. Data are presented as the mean ±SEM. Representative data from three independent experiments are shown. (C) Dose-response curves for ZNL-3, ZNL-3r, and osimertinib in EGFR L858R/C775S Ba/F3 cells following 72 h of treatment. Cell viability was assessed with CellTiter-Glo. Data are presented as the mean ± SEM. Representative data from three independent experiments are shown. (D) Competitive pull-down assay in L858R/C797S Ba/F3 cells treated with ZNL-3 or Osimertinib at the indicated concentrations for 6 h. Cell lysates were incubated with bio-ZNL-0013. (E) Effects of ZNL-3 on EGFR signaling pathway in EGFR L858R/C797S Ba/F3 after 6 h treatment. (F) The in vivo antitumor efficacy of ZNL-3 in EGFR L858R/C797S-driven tumors (*n* = 9 per group). Data are presented as the mean ± SEM. (G) The body weight of EGFR L858R/C797S xenograft model (*n* = 9 per group). Data are presented as the mean ± SEM.

Supported by biochemical and cellular data, ZNL-3 was advanced to a mouse pharmacokinetic (PK) study and an in vivo efficacy study with a xenograft model of L858R/C797S Ba/F3 cells. In the PK study, mice were dosed with 1 mg/kg and 10 mg/kg of IV and PO, respectively. Analysis of mice plasma data demonstrated that ZNL-3 exhibits a moderate IV half-life of approximately 1 h and achieves greater than 100% oral bioavailability (Table S3). As shown in Figure 2F, ZNL-3 treatment with a regimen of 50 mg/kg twice daily (BID) resulted in significant tumor growth inhibition with regression during the treatment period. Tumor regrowth was only observed on the days after the drug was withdrawn. The compound was well-tolerated with an average body weight loss of <3% (Figure 2F and 2G).

ZNL-3 also exhibits an antiproliferative IC_50_ of less than 100 nM against the established human tumor derived cancer cell lines H3255 and PC-9, which harbor L858R and Del-19, respectively (Figure 3A). Furthermore, ZNL-3 inhibits phosphorylation of p-EGFR, p-ERK and p-AKT with a dose-dependent manner in H3255 and PC9, with effects observed at concentrations between 10 to 50 nM (Figure 3B).

**Figure 3:**
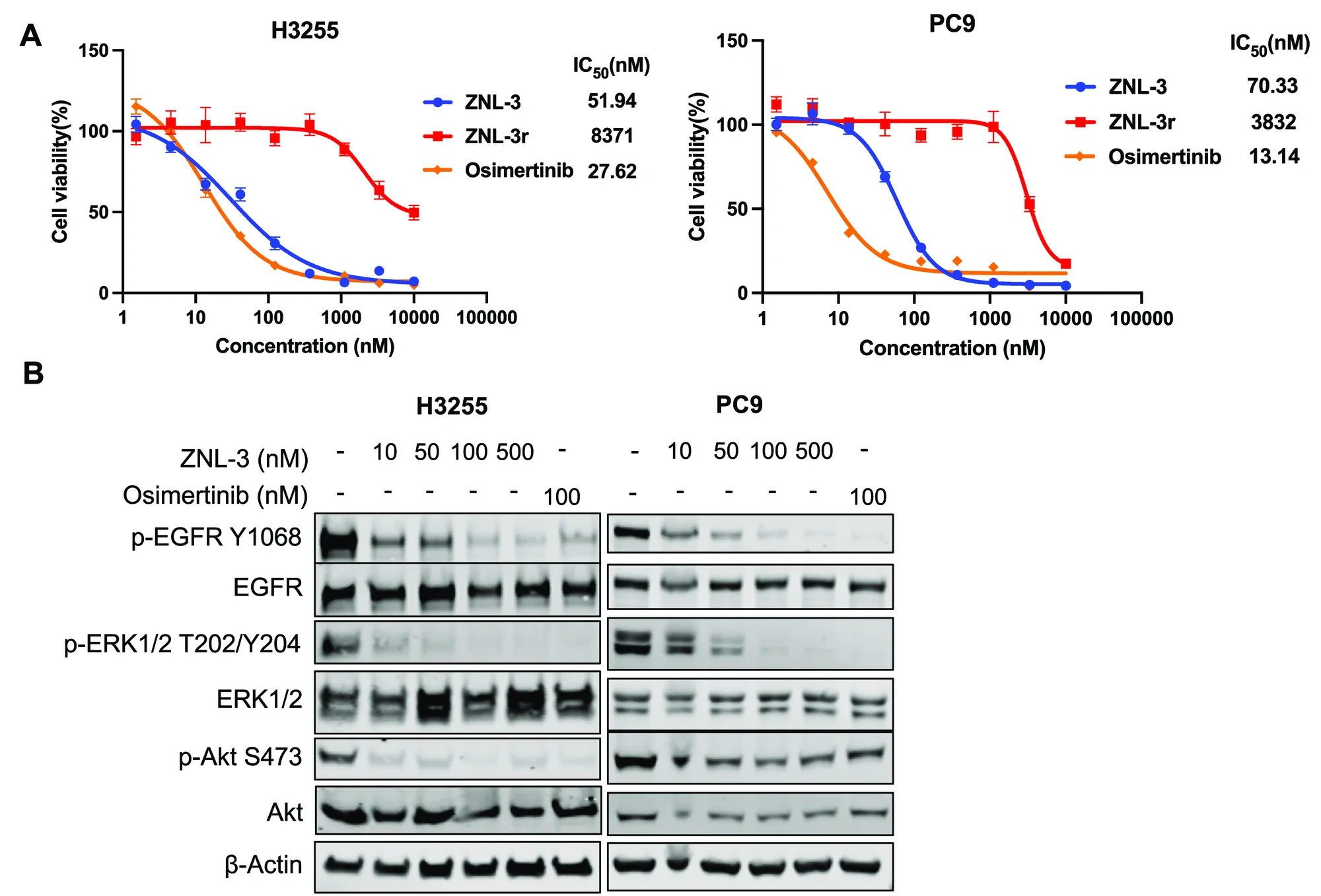
Antiproliferative and downstream inhibitory effect of ZNL-3 in human NSCLC cells. (A) Dose-response curves for ZNL-3, ZNL-3r in H3255 and PC9 following 72 h of treatment. Cell viability was assessed with CellTiter-Glo. Data are presented as the mean±SEM. Representative data from three independent experiments are shown. (B) Effects of ZNL-3 on EGFR signaling pathway in H3255 and PC9 after 6 h of treatment. Representative data from three independent experiments are shown.

### Cysteine 775-Directed Approach for the Design of Bident EGFR Inhibitor YNW-1

The first-generation bident EGFR inhibitor ZNL-0056 was designed from a molecule that labels front C797, and the optimization did not yield a potent covalent inhibitor through targeting C775 alone.^16^ Therefore, ZNL-0056 is inadequate for inhibition of the C797S mutant. The robust covalent engagement of C775 led us to envision that ZNL-3 might represent a novel starting point for a reverse approach to installing a second acrylamide warhead for C797. With this hypothesis, we introduced acrylamide-equipped pyrrolidine, piperidine or phenyl ring to the sugar pocket and reduced the molecular size by eliminating the solvent-exposed phenylpiperazine group, leading to compounds ZNL-4, ZNL-5, and ZNL-6 (Figure 4A). All these designs maintained high anti-proliferative activity in L858R Ba/F3 cells with IC_50_s below 100 nM. To elucidate if these bident compounds can exert the activity through independent engagement on either cysteine, we evaluated their activity in Ba/F3 cells expressing either C797S or C775S mutations. Unfortunately, these compounds lost between 20 and 140-fold activity against both mutations with C797S having the greatest impact (Figure 4B and Figure S3A). These results suggest that covalent engagement at C775 is substantially less efficient than that at C797. We hypothesized that improving the reversible binding for the residence time independent of covalency might achieve this balance. To improve the reversible binding, we re-introduced the hinge binding anilinopiperazine groups to generate ZNL-7 and ZNL-8, which exhibited sub-100 nM IC_50_ against both C797S and C775 mutations (Figure 4A and 4B, and Figure S3A).

**Figure 4:**
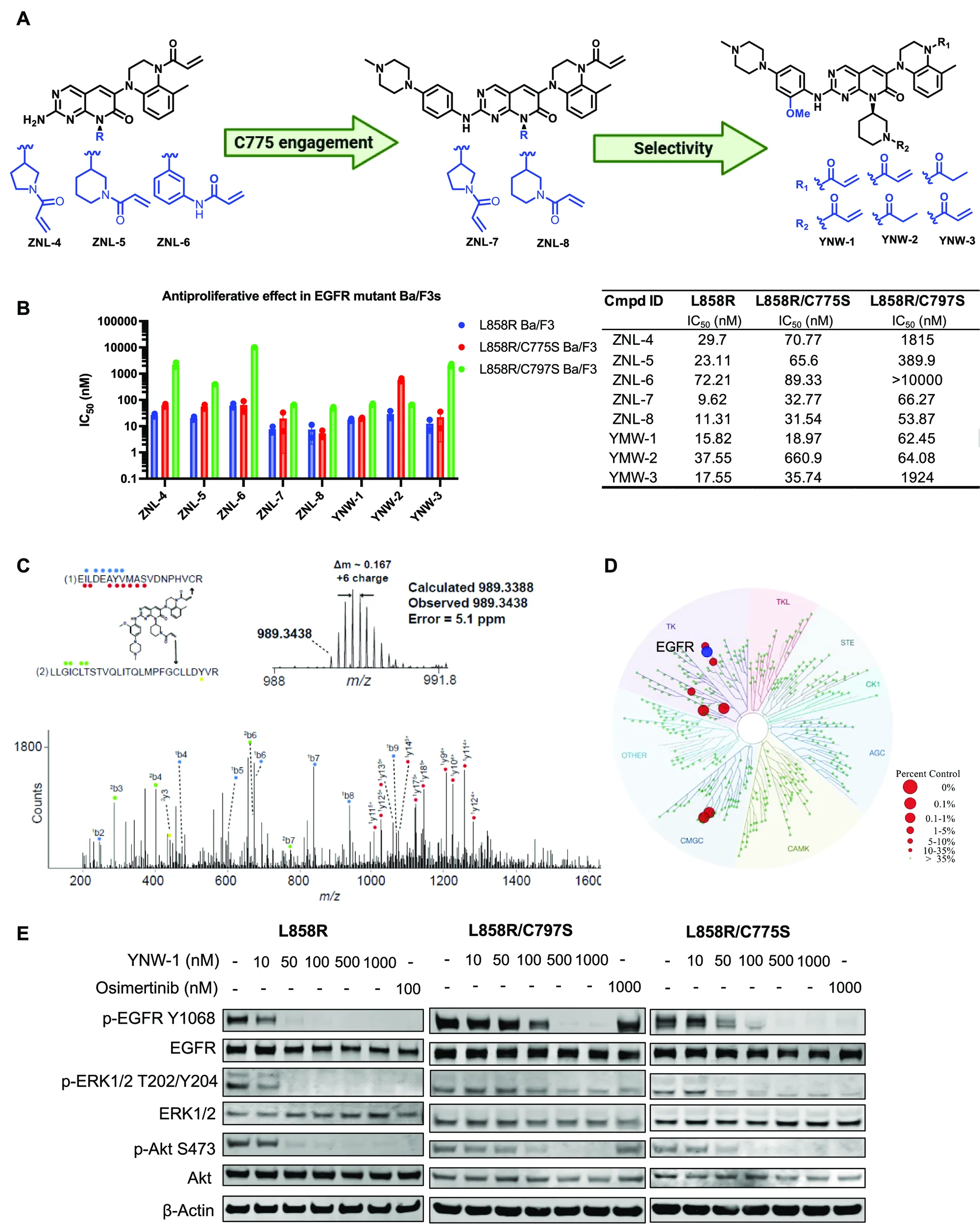
Cysteine 775-directed approach for the design of bident EGFR inhibitor YNW-1. (A) Compound structures for EGFR dual cysteine-targeting molecules. (B) Average IC50 values (mean ± SEM) of the indicated compounds in EGFR L858R Ba/F3, EGFR L858R/C775S Ba/F3, and EGFR L858R/C797S Ba/F3 cells after 72 h treatment. Data are presented as the mean±SEM. Representative data from three independent experiments are shown. (C) MS/MS spectrum for YNW-1 covalently modifying Cys775 and Cys797 of EGFR. Red and yellow circles indicate ions of type y for sequences marked (1) and (2), respectively. Blue and green circles indicate ions of b for sequences (1) and (2), respectively. [Legend: (*) carbamidomethyl cysteine, (Δ) oxidized methionine.] (D) KINOMEscan profiling of YNW-1 at 1 μM. (E) Effects of YNW-1 on EGFR signaling pathway in EGFR L858R Ba/F3, EGFR L858R/C775S Ba/F3, and EGFR L858R/C797S Ba/F3 after 6 h of treatment. Representative data from three independent experiments are shown.

One of the concerns for bident compounds is the potential for increased promiscuity due to the presence of a second electrophile warhead. To assess this, we conducted KinomeScan profiling. At 1 μM, ZNL-8 (Figure S3B) demonstrated moderate selectivity, with 37 off-targets based on the fraction of kinases inhibited by >90% (Table S4). YNW-1 was designed to incorporate a 2-methoxy group to the aniline, which is a well-known kinase selectivity-enhancing modification (Figure 4A). To confirm the covalent bond formation with Cys797 and Cys775 by YNW-1, we conducted mass spectrometry with trypsin digestion of purified recombinant EGFR^L858R^ kinase domain treated by YNW-1, identified cross-linked peptide corresponding to Cys797 and Cys775, supporting the conclusion that YNW-1 forms simultaneous, intramolecular covalent bonds with both residues (Figure 4C). YNW-1 demonstrated a highly improved selectivity profile across the whole kinome and only 7 kinases were detected as potential off-targets (>90% inhibition) (Figure 4D and Table S5), none of which contains a targetable cysteine (Table S6) in the kinase domain. Furthermore, YNW-1 exhibited robust EGFR L858R inhibition with an apparent IC_50_ below 10 nM in both cellular and biochemical levels (Figure 4B and Figure S3C). The anti-proliferative IC_50_ in Ba/F3 remained potent (19 nM and 62 nM) regardless of the mutations on either C775 or C797 (Figure S3A), but became inactive once the simultaneous mutations on both cysteines occurred (Figure S3D). This equal independence on cysteines was further evidenced by the evaluation of two control compounds YNW-2 and YNW-3, which were designed as mono-covalent inhibitors targeting C775 and C797, respectively (Figure 4A). They unambiguously demonstrated the necessity of cysteine-targeting as its anti-proliferation activity was significantly diminished once its designated cysteine was mutated (Figure 4B and Figure S3A). Consistent with anti-proliferation activity, the downstream signaling including p-EGFR/ERK and AKT were inhibited by 50-100 nM of YNW-1 with background of L858R, L858R/C797S, and L858R/C775S EGFR mutants (Figure 4E).

### YNW-1 has Broad Activity against EGFR Mutants and Delayed Acquired Mutations

The unique mechanism of action for YNW-1 prompted us to investigate two potential advantages: resilience to a broad spectrum of EGFR mutants and suppression of acquired point mutations. First, we tested YNW-1 in Ba/F3 cells engineered with a DEL19 background. As shown in Figure 5A, it unveiled a similar activity across Del19, Del19/T790M, and Del19/C797S. Second, the compound was active against three exon 20 insertion mutations (insASV, insSVD, and insFQEA), which universally displayed sensitivity with an IC_50_ ranging from 100 nM to 250 nM (Figure 5B). Other rarely occurred mutations including E790K, L861Q, and G719A/E709K were also susceptible to YNW-1 (Figure 5C). While del18, G719A, S768I, and G719A/T790M are less susceptible to YNW-1 (Figure S4). Notably, YNW-2 and YNW-3 showed reduced activity across these mutations, consistent with the enhanced potency conferred by the dual covalent bonding mechanism of YNW-1.

**Figure 5:**
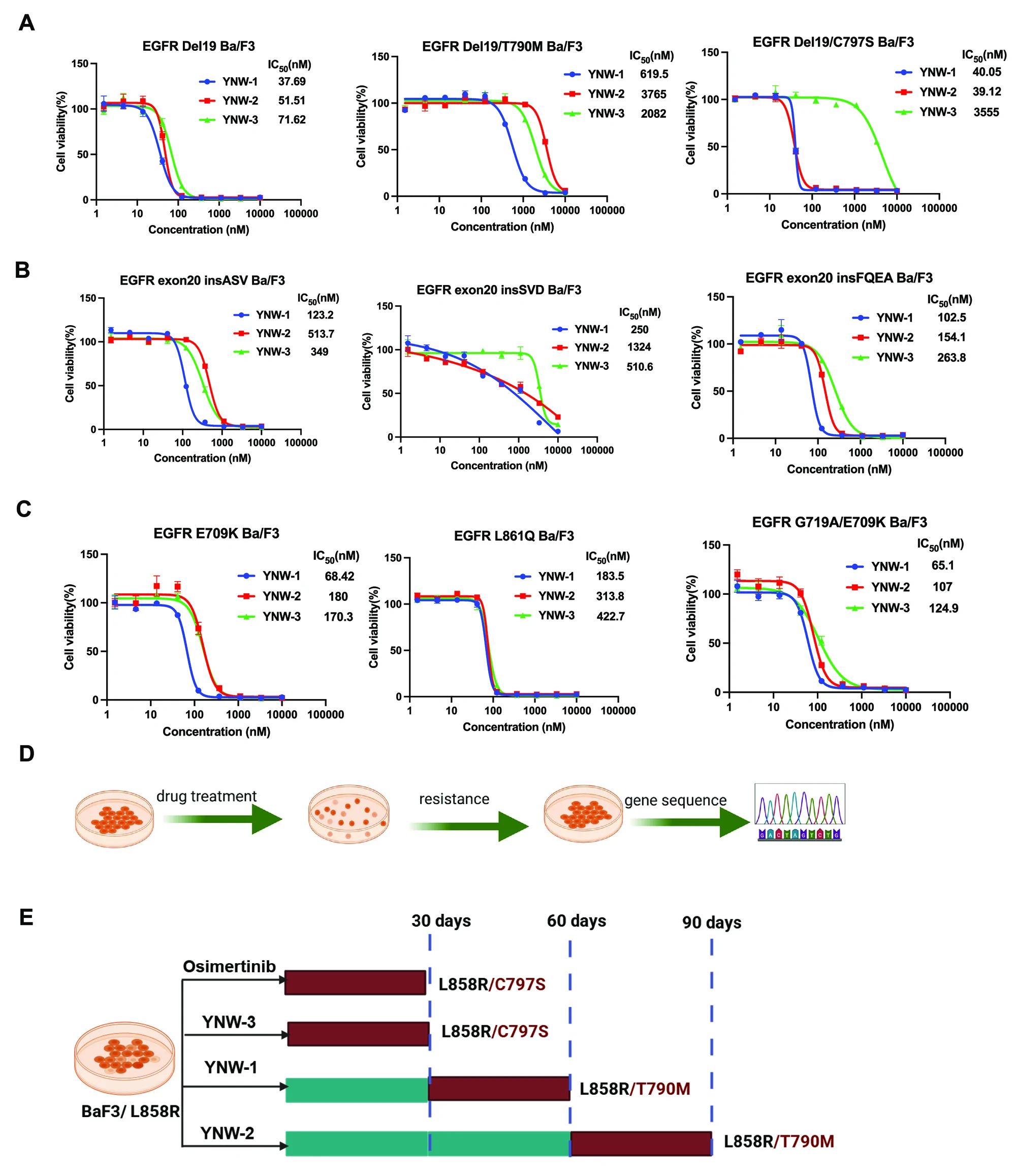
YNW-1 has broad activity against EGFR mutants and delayed acquired mutations. (A) Dose-response curves for YNW-1, YNW-2, YNW-3 in EGFR Del19 Ba/F3, EGFR Del19/T790M Ba/F3 and EGFR Del19/C797S Ba/F3 after 72 h treatment. Cell viability was assessed with CellTiter-Glo. Data are presented as mean ± SEM. Representative data from three independent experiments are shown. (B) Dose-response curves for YNW-1, YNW-2, YNW-3 in EGFR exon20 insASV Ba/F3, EGFR exon20 insSVD Ba/F3 and EGFR exon20 insFQEA Ba/F3 after 72 h treatment. Cell viability was assessed with CellTiter-Glo. Data are presented as the mean ± SEM. Representative data from three independent experiments are shown. (C) Dose-response curves for YNW-1, YNW-2, YNW-3 in EGFR E709 K Ba/F3, EGFR L861Q Ba/F3, and EGFR G719A/E709 K Ba/F3 after 72 h treatment. Cell viability was assessed with CellTiter-Glo. Data are presented as mean ± SEM. Representative data from three independent experiments are shown. (D) Schematic illustration of chronic exposure for YNW-1 acquired resistant mutation. (E) Mutations found in EGFR kinase domain for YNW-1, YNW-2, YNW-3, and osimertinib in EGFR L858R Ba/F3-resistant cells.

We next performed a chronic drug-exposure study, gradually escalating the concentrations of YNW-1, YNW-2, and YNW-3 until cells were able to proliferate normally at 500 nM. To assess whether resistance mechanisms differ between bident and monovalent inhibitors (Figure 5D). We harvested persistent cells for sequence analysis on EGFR after one-, two-, and three-month continuous drug exposure. As shown in Figure 5E and Figure S5, osimertinib and YNW-3 rapidly induced the C797S mutation within one month, whereas cells treated with the bident inhibitors YNW-1 and YNW-2 remained in the wild-type state. Interestingly, the bident compound began to induce the gatekeeper T790M mutation instead of the mutation on cysteines in two months. However, this mutation was absent with YNW-2 at this time point but emerged only after three-month treatments. The results indicate that targeting C775 might have a slow onset of acquired resistance through EGFR mutation compared with osimertinib under these experimental conditions. However, resistance mechanisms identified in this in-vitro model may not fully reflect the more complex and heterogeneous mechanisms that emerge clinically.

## Discussion

The emergence of resistance to the front-line therapy Osimertinib through both EGFR-dependent and EGFR-independent mechanisms remains an unmet medical need. Although multiple drug-resistant EGFR mutations have been identified, the C797S mutation accounts for a substantial proportion of patients who relapse following Osimertinib treatment. ZNL-3, a first-in-class cysteine 775-targeted covalent inhibitor for EGFR, has demonstrated promising therapeutic potential to overcome this resistance. Both in-vitro and in-vivo efficacy study suggested that ZNL-3 potently inhibited mutant EGFR signaling and drove tumor regression with good tolerability. ZNL-3 represents a promising alternative strategy for targeting C797S-mediated resistance, demonstrating robust antitumor activity and selectivity. Its mode of action differentiates it from reversible attempts by retaining the remarkable efficacy of covalent inhibitors for mutant EGFR NSCLC demonstrated by Osimertinib while addressing the C797S resistance. While ZNL-3 serves as a promising lead compound targeting the L858R and L858R/C797S mutations, the activity suffers from the T790M mutation, displaying IC_50_ values above 1 μM in Ba/F3 cells (Figure S6A), and losing activity in H3255GR and DFCI52 cells that harbor the T790M gatekeeper mutation (Figure S6B). Further optimization of ZNL-3 to overcome this limitation is underway. On the other hand, T790M in the real world mEGFR NSCLC patient population is diminishing due to the first line treatment of Osimertinib; clinical candidates that stemmed from ZNL-3 can be readily developed to address the drug-resistant mutants as the second line therapy. In addition, it can be used in combination with Osimertinib to delay the occurrence of drug resistance, even though both compounds are mutually exclusive when binding to an EGFR protein, since a double mutation (C797S/C775S) in cis would be required to confer resistance to the combination therapy.

The bident compound built upon ZNL-3 provides a new strategy and a breakthrough in the discovery of a new modality. YNW-1 is the first bident molecule disclosed so far with a nearly equal efficiency on both cysteines, despite drastically different protein microenvironments, demonstrating that small molecules can mitigate different nucleophilic reactivities within one molecule, a first in its class. The unambiguous characterization of YNW-1 with high selectivity, broad activity across EGFR mutants, and, more importantly, a slow onset of acquired point mutation compared to osimertinib is intriguing. Here, we observed that the gatekeeper mutation T790M emerged rather than either of cysteines for bident compound YNW-1, which is consistent with the observed IC_50_ on L858R/T790M (136 nM) (Figure S7A and S7B), compared to L858R/C797S (62 nM) and L858R/C775S (19 nM) (Figure S3A). It points to the direction to further optimize the inhibition of T790M for the YNW-1 chemical series. We believe this is an addressable issue with medicinal chemistry effort rather than an intrinsic weakness of the modality. Since a single mutation on either cysteine is incapable of causing resistance, the point mutation on EGFR is predicted as a minor resistance mechanism for a potent L858R/T790M bident inhibitor. Finally, the observation of delayed mutagenesis on T790M for YNW-3 was encouraging and might imply a potential therapeutic superiority to osimertinib. It may also pose an advantage over osimertinib combination therapy by less drug load to reduce any potential side effects. Unquestionably, the development of an optimized version of YNW-1 or ZNL-3 that overcomes T790M will be a major next step and offers a new strategy to manage mEGFR-driven non-small-cell lung cancer, which is an ongoing effort in our laboratory and will be reported in a following publication.

## Methods

### Crystallization and Structure Determination

Wild-type EGFR (residues 696-1022) was purified as described for other variants. Protein at ∼3 mg/mL was incubated with 0.5 mM ZNL-1 and incubated for 1 h at room temperature. Sitting drop screens were set with drops containing 0.2 μL protein and 0.2 μL reservoir solution adjacent to 50 μL reservoir solution. Screens were incubated at room temperature and crystals of two morphologies (rods and diamonds) observed after ∼1 month in condition C1 (3.5 M sodium formate (pH 7.0)) of the Index-HT screen (Hampton Research). Crystals were harvested directly from the screening tray and cryoprotected in a solution containing 3.5 M sodium formate (pH 7.0) and 25% ethylene glycol prior to flash freezing in liquid nitrogen.

Diffraction data were collected under a cryostream at NE-CAT beamlines at the Advanced Photon Source at the Argonne National Laboratory. Crystals with rod morphology diffracted significantly better than did diamond crystals. Data were processed using Dials via xia2 and SBGrid.^18,19^ The structure was phased via molecular replacement with PDB entry 2GS2, and the N-lobe of the kinase was manually rebuilt due to large rotational conformational changes.^20^ Refinement was performed using Phenix and manual model building in Coot.^21,22^ Custom ligand restraints were generated using eLBOW in Phenix with AM1 quantum-mechanical optimization.^23^ The resulting structure has been deposited in the Protein Data Bank (PDB) with accession code 9PWI.

### Mass Spectrometry Analysis

EGFR protein was treated with a 5-fold molar excess of indicated probe or DMSO for 2 h at 30 °C in 50 mM HEPES buffer 100 mM NaCl pH 7.5. Reactions were analyzed by LC-MS with a Shimadzu HPLC system (Shimadzu, Marlborough, MA) interfaced to an LTQ ion trap mass spectrometer (Thermofisher Scientific, San Jose, CA) operated in profile mode (*m*/*z* 300–2000, spray voltage = 5 kV). Proteins were desalted for 1 min with solvent A before eluting to the mass spectrometer with a ballistic gradient (0–100% B in 1 min, A = 0.1% formic acid in water, B = 0.1% formic acid in acetonitrile). Raw data were deconvoluted with Magtran 1.03b software.^24^

To identify sites of dual warhead crosslinking, protein was treated as described above and then reduced (10 mM dithiothreitol, 37 °C, 30 min), alkylated (22.5 mM iodoacetamide, 30 min, room temperature, protected from light), and digested overnight with trypsin (1:20 trypsin:EGFR; PROMEGA, Madison, WI) at 37 °C. Peptides were loaded onto EVO tips and analyzed with a 20-SPD method on an EVO Sep One (column = 75 μm I.D. packed with 15 cm × 1.5 μm Dr. Maisch C18 [ESI Source Solutions, Woburn, MA] with integrated emitter tip^25^) coupled to a Bruker timsTOF Pro2 mass spectrometer operated in DDA-PASEF mode. Dual warhead targets were identified by calculating *m*/*z* values for expected cross-linked peptides using the PepCalc tool in mzStudio.^26^ This tool was then used to make extracted ion chromatograms (XICs) for expected charge states of the cross-linked peptides (typically from +4 to +7). We then examined MS/MS spectra within XIC elution profiles that were identified with the “Find Precursors” function of PepCalc. We identified the cross-linked peptides by mapping *y*- and *b*-type ions onto MS spectra, as shown in Figure 4C.

### Cell Culture

H3255­(RRID: CVCL_6831) and H3255GR were cultured in ACL-4 media^27^ containing 10% fetal bovine serum (GeminiBio, Cat. No. 100-106) and 1% penicillin/streptomycin. The EGFR mutant Ba/F3 cells were generated and characterized as described previously,^11^ and cultured in RPMI media (Life Technologies, Cat. No. 11875119) containing 10% fetal bovine serum and 1% penicillin/streptomycin. All the cell lines were cultured at 37 °C in 5% CO_2_ humidified air and tested for mycoplasma negative.

### Immunoblotting and Antibodies

Cells were lysed in RIPA buffer (150 mM NaCl, 1.0% IGEPAL CA-630, 0.5% sodium deoxycholate, 0.1% SDS, 50 mM Tris, pH 8.0) (Sigma, Cat. No. R0278) with protease inhibitor and phosphatase inhibitor (Roche). The protein concentrations were measured by BCA analysis (Thermo Fisher Scientific, Cat. No. PI23225). Equal amounts of protein were resolved by 4%–12% Tris-Base gels (Life Technologies) and then transferred to the Immuno-Blot PVDF membrane (BioRad, Cat. No. 1620177). Proteins were probed with appropriate primary antibodies at 4 °C overnight and then with IRDye 800-labeled goat anti-rabbit IgG (LICOR Biosciences, Cat. No. 926-32211, RRID: AB_621843), IRDye 800-labeled goat anti-mouse IgG (LICOR Biosciences, Cat. No. 926-32210, RRID: AB_621842) or IRDye 680RD goat anti-Mouse IgG (LICOR Biosciences, Cat. No. 926-68070, RRID: AB_10956588) secondary antibodies at room temperature for 1 h. The membranes were detected on the Odyssey CLx system.

Antibodies used in this study include anti-following proteins: EGFR (Cell Signaling Technology, No. 4267S, 1:1000, RRID: AB_2895042), p-EGFR (Tyr1068) (Cell Signaling Technology, No. 3777S, 1:1000, RRID: AB_2096270), ERK1/2 (Cell Signaling Technology, No. 4696S, 1:1000, RRID: AB_390780), p-ERK1/2 (Cell Signaling Technology, No. 4370S, 1:1000, RRID: AB_2315112), Akt (Cell Signaling Technology, No. 9272L, 1:1000, RRID: AB_329827), p-Akt (Cell Signaling Technology, No. 4060S, 1:1000, RRID: AB_2315049) and β-Actin (Cell Signaling Technology, No. 3700, 1:1000, AB_10828322).

### Antiproliferation Assay

Cells were seeded at the density of 1000 cells/well for 384-well plates. For adherent cell lines, cells were cultured for 16 h before compounds were added into the media for 72 h treatment. Cell viability was determined by using CellTiter-Glo (Promega No. G7571) according to the manufacturer’s instructions, measuring luminescence using an FLUOstar Omega plate reader (BMG LABTEC). Dose-response curves were generated using nonlinear regression curve fit in GraphPad Prism (RRID: SCR_002798).

### Cellular Target Engagement Assays

After 6 h treatment, cells were pelleted, washed with PBS once and lysed with IP lysis buffer (25 mM Tris-HCl pH 7.4, 150 mM NaCl, 1 mM EDTA, 1% NP-40, and 5% glycerol) (Thermo Fisher Scientific, Cat. No. 87788) containing protease/phosphatase inhibitor cocktail (Roche). The protein concentrations were measured by BCA analysis (Pierce). Cell lysates were incubated with 1 μM of biotin conjugated probe (bio-Osimertinib or ZNL-0130) at 4 °C overnight, and incubated for 3 more hours at room temperature. Lysates with probe were then incubated with streptavidin beads (Thermo Fisher, No. 20349) for 2 h at 4 °C. The protein–probe complexes on the beads were then subjected to immunoblotting.

### Protein Expression and Purification

The human EGFR kinase domain (Residues 696–1022) harboring the L858R, L858R/T790M, L858R/C775S, or L858R/C797S mutations was cloned into a pFastBac vector with an N-terminal His-GST tag containing a TEV protease cleavage site. Recombinant proteins were expressed in *Spodoptera frugiperda* (SF9) insect cells using baculovirus generated via the pFastBac system (Invitrogen). SF9 cells were cultured to a density of approximately 1.5–2 × 10^6^ cells/mL and infected with 1%–2% (v/v) virus stock. After 68–72 h of incubation, cells were harvested via centrifugation and the resulting cell pellets were stored at −80 °C.

For protein extraction, frozen cell pellets were resuspended and lysed in buffer A comprised of 50 mM Tris-HCl, pH 8.0, 500 mM NaCl, 5% glycerol, 0.5 mM Tris­(2-carboxyethyl) phosphine [TCEP]). The resulting solution was lysed via sonication. Lysates were clarified by ultracentrifugation at >200 000*g* for 1 h at 4 °C. The resulting lysate was filtered with a 2 μm glass filter before being applied to Ni-NTA agarose resin by gravity flow. The resin was washed with buffer A supplemented with 40 mM imidazole and the bound protein was eluted with buffer A containing 200 mM imidazole.

To cleave the His-GST tag, a stoichiometric excess of in-house purified TEV protease was added to the eluate, and the mixture was dialyzed overnight at 4 °C in ∼1 L of buffer A. Following protease digestion, the sample was re-applied to Ni-NTA resin to remove the cleaved tag and uncleaved protein. The kinase domain was then further purified by size-exclusion chromatography on a Superdex 200 (S200) column (Cytiva) equilibrated in buffer A. Fractions containing the target protein with a purity of ≥95% (confirmed by SDS-PAGE) were pooled and concentrated for subsequent experiments.

### Kinase Inhibition Assay

Kinase inhibition assays were conducted using the HTRF KinEASE TK assay kit (Cisbio), following the manufacturer’s instructions. Small-molecule inhibitors were prepared from 10 mM DMSO stock solutions and dispensed into black 384-well plates using a D300e Digital Dispenser (Hewlett–Packard), normalized to a final DMSO concentration of 1% (v/v) in all wells.

Purified EGFR kinase domain was diluted to a final concentration of 0.2 nM in assay buffer and dispensed into the plates using a Multidrop Combi dispenser (Thermo Fisher Scientific). Plates were incubated at room temperature for 60 min to allow pre-equilibration with the inhibitors. Reactions were initiated by adding ATP to a final concentration of 100 μM and incubated for an additional 30 min at room temperature.

Reactions were quenched with the detection reagent provided in the KinEASE kit. Fluorescence resonance energy transfer (FRET) signals were measured at 620 and 665 nm using a PHERAstar microplate reader (BMG LABTECH). Data were analyzed in GraphPad Prism 10 and fitted to a three-parameter dose–response model to determine inhibitor potency.

### Efficacy Study with Mice

The efficacy study was conducted at Dana-Farber Cancer Institute with the approval of the Institutional Animal Care and Use Committee under Protocol No. 04-111 in an AAALAC accredited vivarium.

The L858R/C797S Ba/F3 cells, grown in RPMI-1640 with 10% FBS, were harvested and 2 million cells with 30% Matrigel implanted subcutaneously in female NOD.Cg-*Prkdc* ^*scid*^ *Il2rg* ^*tm1Wjl*^/SzJ (NSG) mice from The Jackson Laboratories (ME). Tumors were allowed to establish to 104.9 ± 11.9 mm^3^ in size before randomization into treatment groups (Studylog, CA software) with *n* = 9/group as vehicle control (10% *N*-methyl-2-pyrrolidone and 90% PEG 300) or ZNL-3 at 50 mg/kg administered twice daily. Mice were treated orally for 21 days. Tumor volumes were determined from caliper measurements by using the formula, Tumor volume = (length × width^2^)/2. Tumor volumes and body weights were measured twice weekly.

## Supplementary Material



## Notes

Electron density analysis of the ZNL-1–EGFR complex; cellular target engagement and EGFR signaling studies; biochemical and cellular potency characterization of ZNL and YNW compounds; kinase selectivity profiling; activity against EGFR rare mutants and resistant cell lines; data collection and refinement statistics; in vitro metabolic stability and mouse pharmacokinetic data; DiscoverX KINOMEscan profiling; compound synthesis and characterization; and NMR characterization data for synthesized compounds (PDF)

## &

Septerna, Inc., South San Francisco, CA, USA
