# Supplementary material for: Overcoming EGFR Resistance by Monovalent and Bident Inhibitors Targeting Cys775
Source: ACS Cent Sci. 2026 Sep 29;12(10):1647–58. doi: 10.1021/acscentsci.6c01219 (PMC13720926; doi:10.1021/acscentsci.6c01219)
Supplement: Supplementary file 1 [file oc6c01219_si_001.pdf]

## Supporting Information

# Overcoming EGFR resistance by monovalent and bident inhibitors targeting Cys775

Zhengnian Li[1,2,10#], Jie Jiang[3,4,#], Yaning Wang[1,2,#], Scott B. Ficarro[9], Stephen J. Collins[5,7], Tyler S. Beyett[5,7], Ilse K. Schaeffner[5,7], Isidoro Tavares[5,6], Felix H. Gottlieb[3,4], Dhiraj Suda[8], Prafulla C.Gokhale[8], Leah M. Black-Holmes[3,4], Dimitris Gazgalis[5,7], Michael J. Eck[5,7], Pasi A. Jänne[3,4], Jarrod A. Marto[9], Jianwei Che[5*], Nathanael S. Gray[1,2*] and Tinghu Zhang[1,2*]

**Abstract:** Covalent targeting of EGFR cysteine 797 by osimertinib is one of the most successful breakthroughs in targeted therapy, fundamentally transforming the treatment landscape for non-small cell lung cancer (NSCLC) patients. However, resistance driven by mutation of C797 remains a major clinical challenge. Developing novel covalent strategies beyond C797 targeting presents a compelling opportunity for next-generation EGFR inhibitors. We first demonstrated that cysteine 775, located deep within the ATP-binding pocket, is accessible by a rationally designed covalent molecule ZNL-3, which as the first-in-class covalent cysteine 775 inhibitor exhibited strong efficacy in osimertinib-resistant mouse models. To further enhance resilience to resistance-causing mutations, we developed a dual-warhead, bident compound YNW-1 which covalently targets both cysteine 775 and 797 simultaneously. YNW-1 is the first intramolecular lock to exhibit balanced reactive efficiency on both cysteines, rendering single-site mutations ineffective to confer resistance. This study establishes the therapeutic potential of an EGFR covalent inhibitor through the unprecedented targeting of cysteine 775. It also provides the first evidence that dual-cysteine engagement offers superior efficacy over conventional covalent inhibitors by delaying the emergence of resistance. Further optimization toward clinical translation is currently ongoing in our laboratory

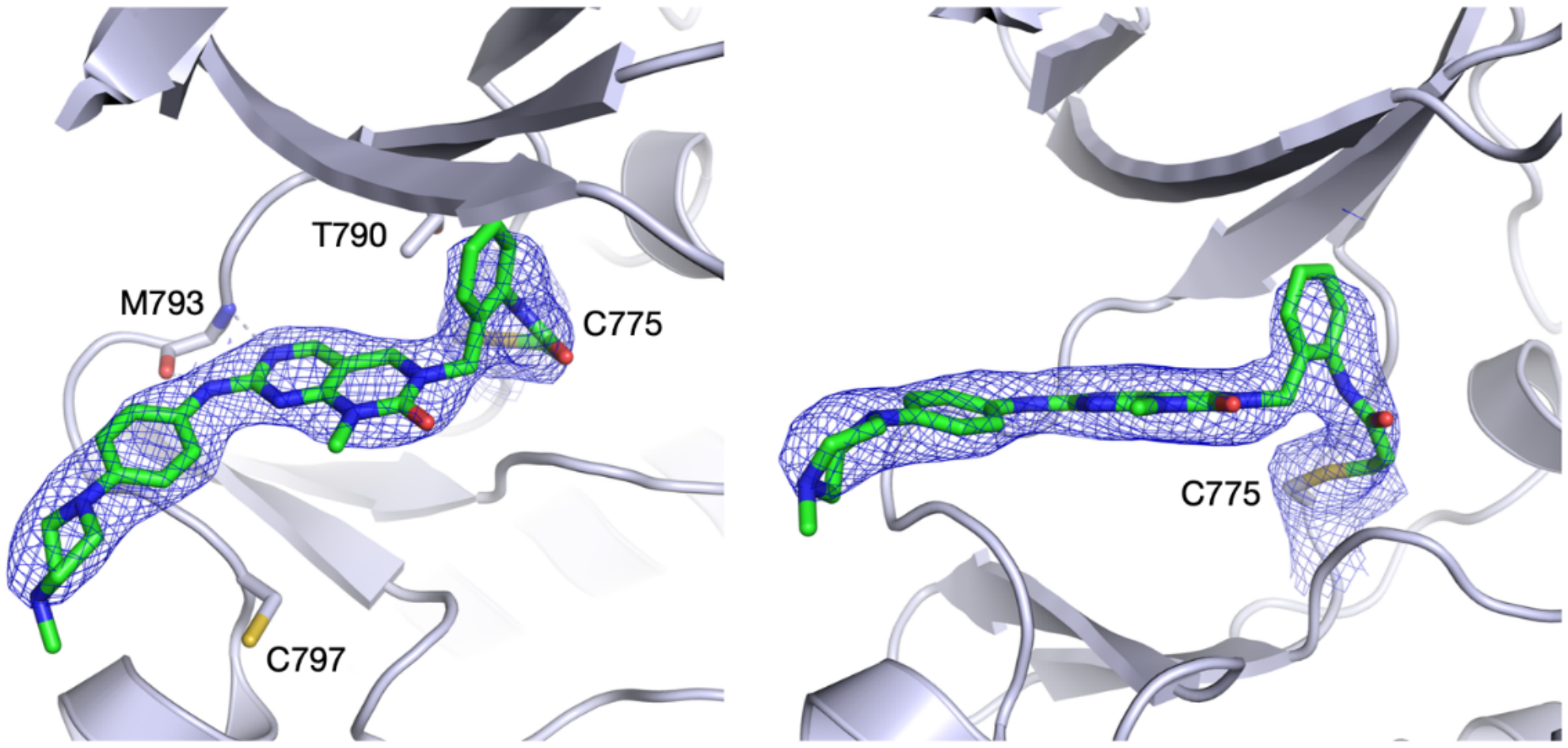


**Figure S1.** Electron density for ZNL-1 bound to EGFR**.** Mesh shown is $2F_o$-$F_c$ contoured at 1 sigma. Density shown includes covalent adduct with C775.

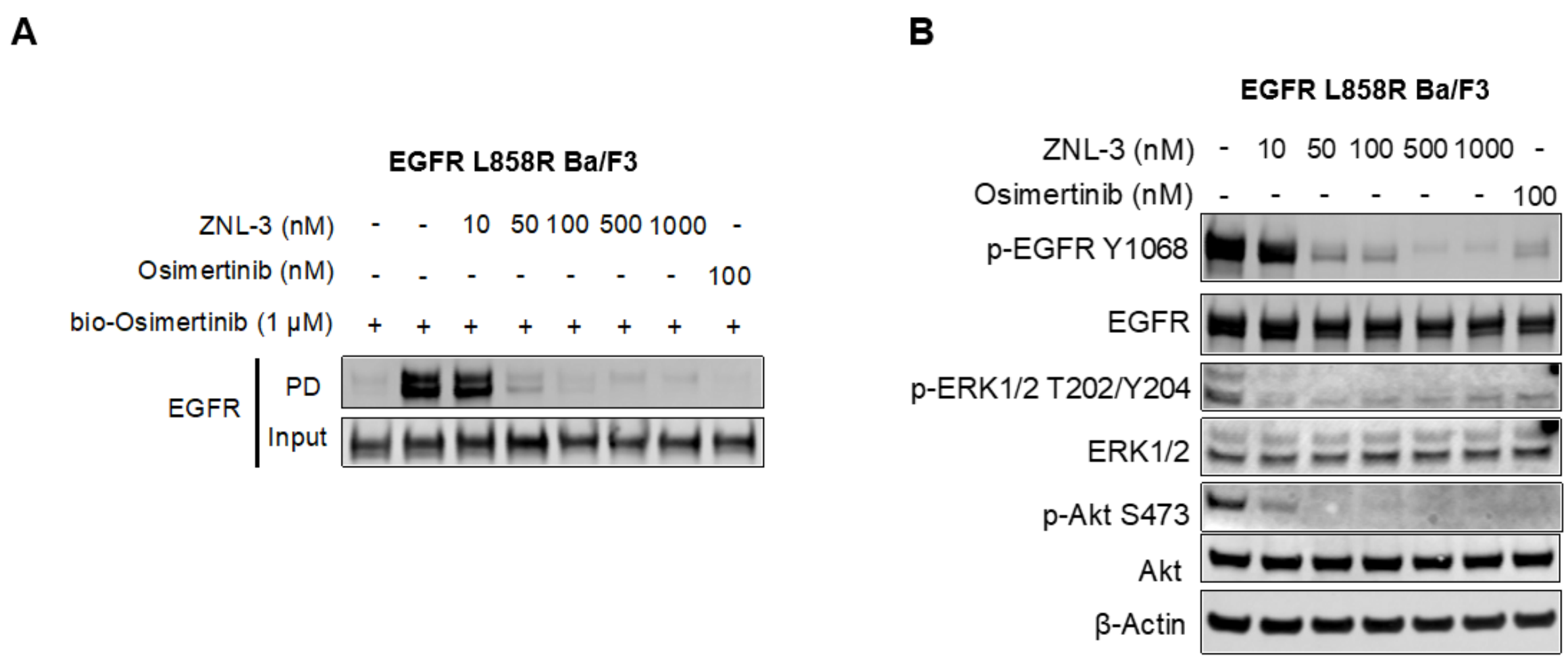


**Figure S2. A**. Competitive pull-down assay in EGFR L858R Ba/F3 cells treated with ZNL-3 or Osimertinib at the indicated concentrations for 6 h. Cell lysates were incubated with bio-osimertinib. **B.** Effects of ZNL-3 on EGFR signaling pathway in EGFR L858R Ba/F3 after 6 h treatment. Representative data from three independent experiments are shown.

**A**

| Cmpd ID | L858R IC$_{50}$ (nM) | L858R/C775S IC$_{50}$ (nM) | L858R/C797S IC$_{50}$ (nM) |
|---|---|---|---|
| ZNL-4 | 29.7 | 70.77 | 1815 |
| ZNL-5 | 23.11 | 65.6 | 389.9 |
| ZNL-6 | 72.21 | 89.33 | >10000 |
| ZNL-7 | 9.62 | 32.77 | 66.27 |
| ZNL-8 | 11.31 | 31.54 | 53.87 |
| YMW-1 | 15.82 | 18.97 | 62.45 |
| YMW-2 | 37.55 | 660.9 | 64.08 |
| YMW-3 | 17.55 | 35.74 | 1924 |

**B**

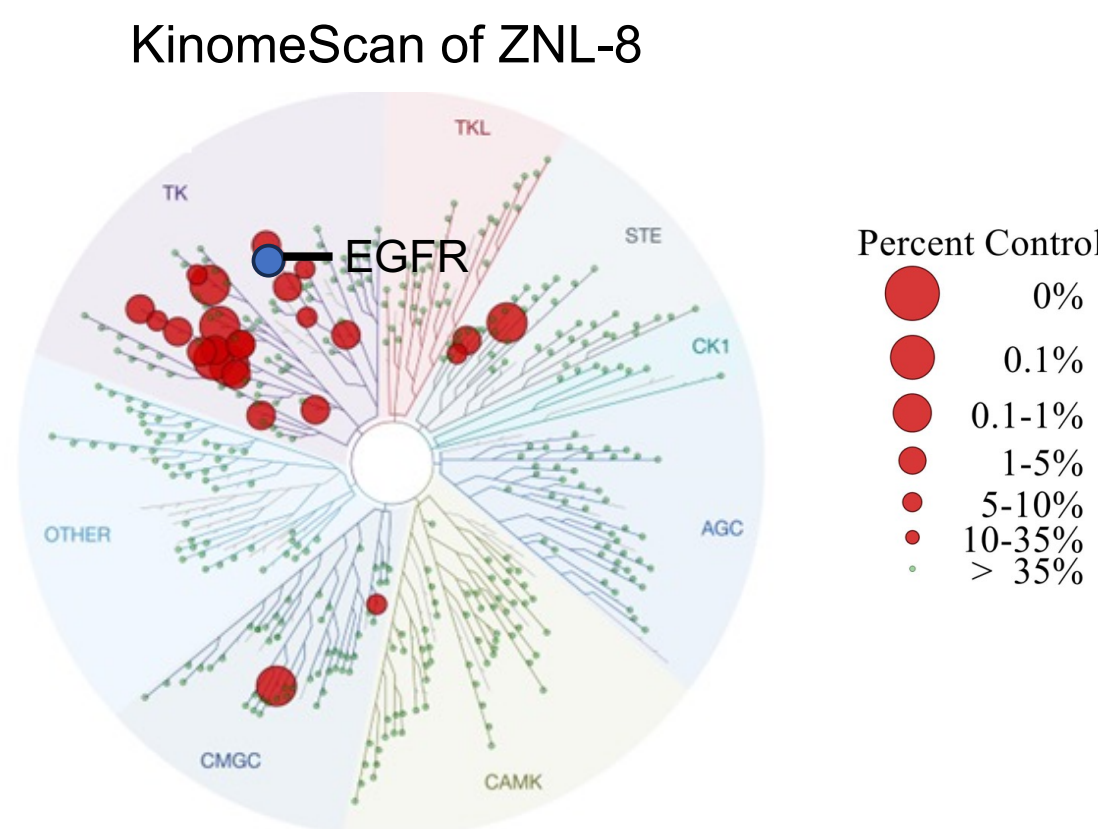


**C**

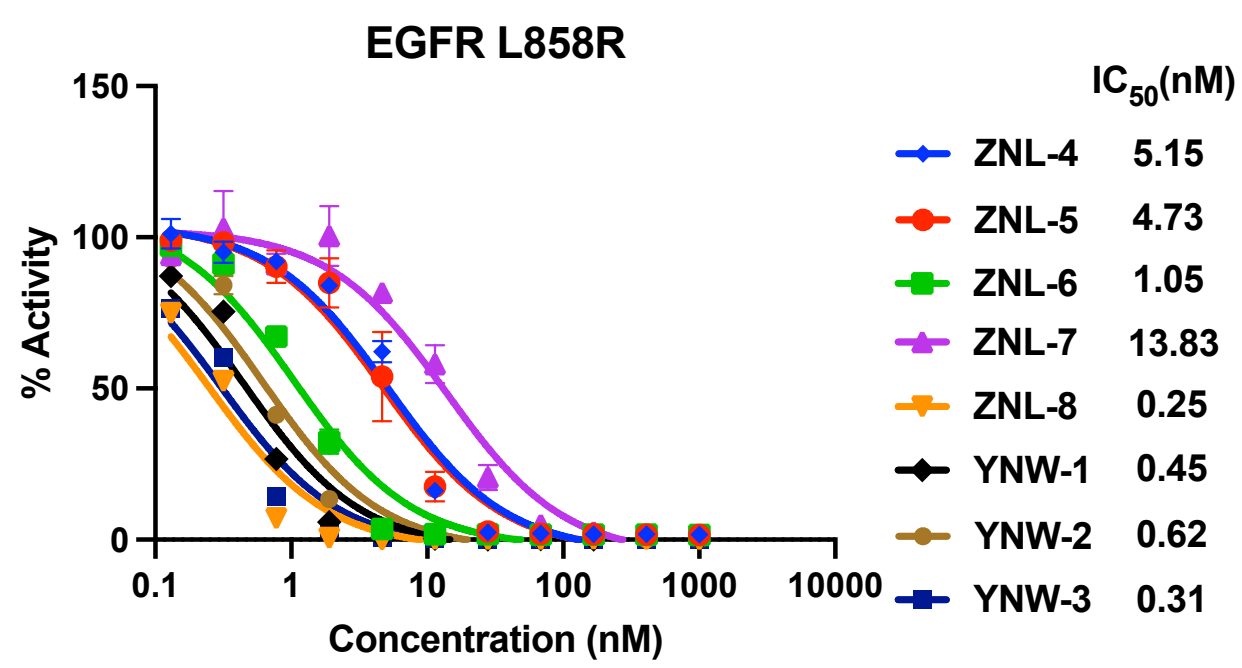


**D**

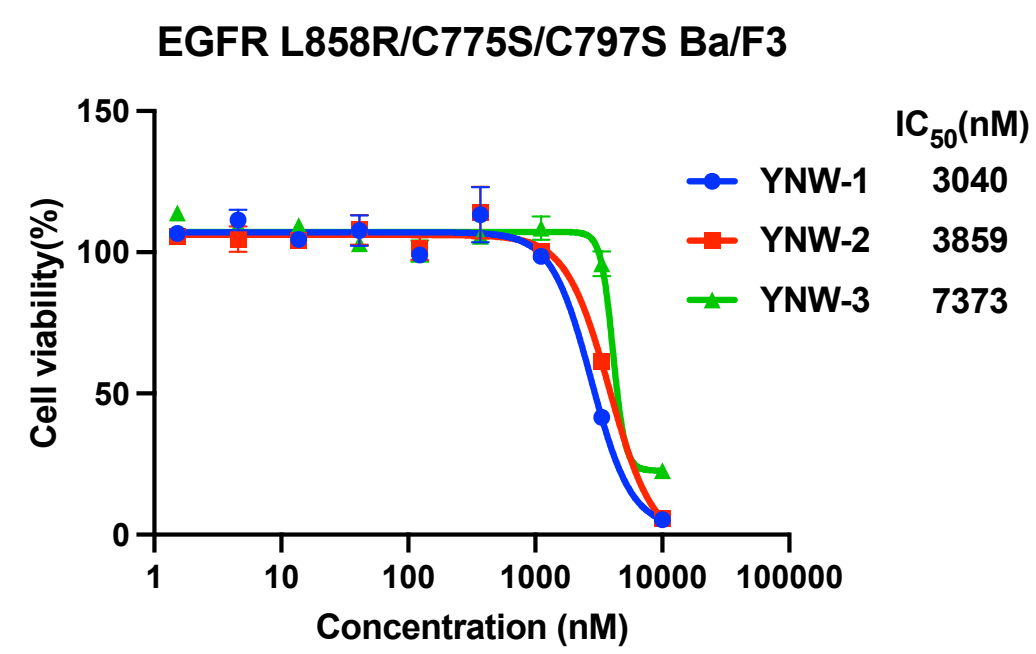


**Figure S3 A.** IC$_{50}$s of ZNL series compounds and YNW-1,2,3 in EGFR L858R, EGFR L858R/C775S and L858R/C797S Ba/F3. Cell viability was assessed with CellTiter-Glo. Representative data from three independent experiments are shown. **B.** The KINOMEscan profiling of ZNL-8 at 1μM. **C.** Biochemical IC50s for the indicated compounds against EGFR L858R. **D.** Dose-response curves for YNW-1, YNW-2 and YNW-3 in EGFR L858R/C775S/C797S Ba/F3 after 72 h treatment. Cell viability was assessed with CellTiter-Glo. Data are presented as the mean±SEM. Representative data from three independent experiments are shown.

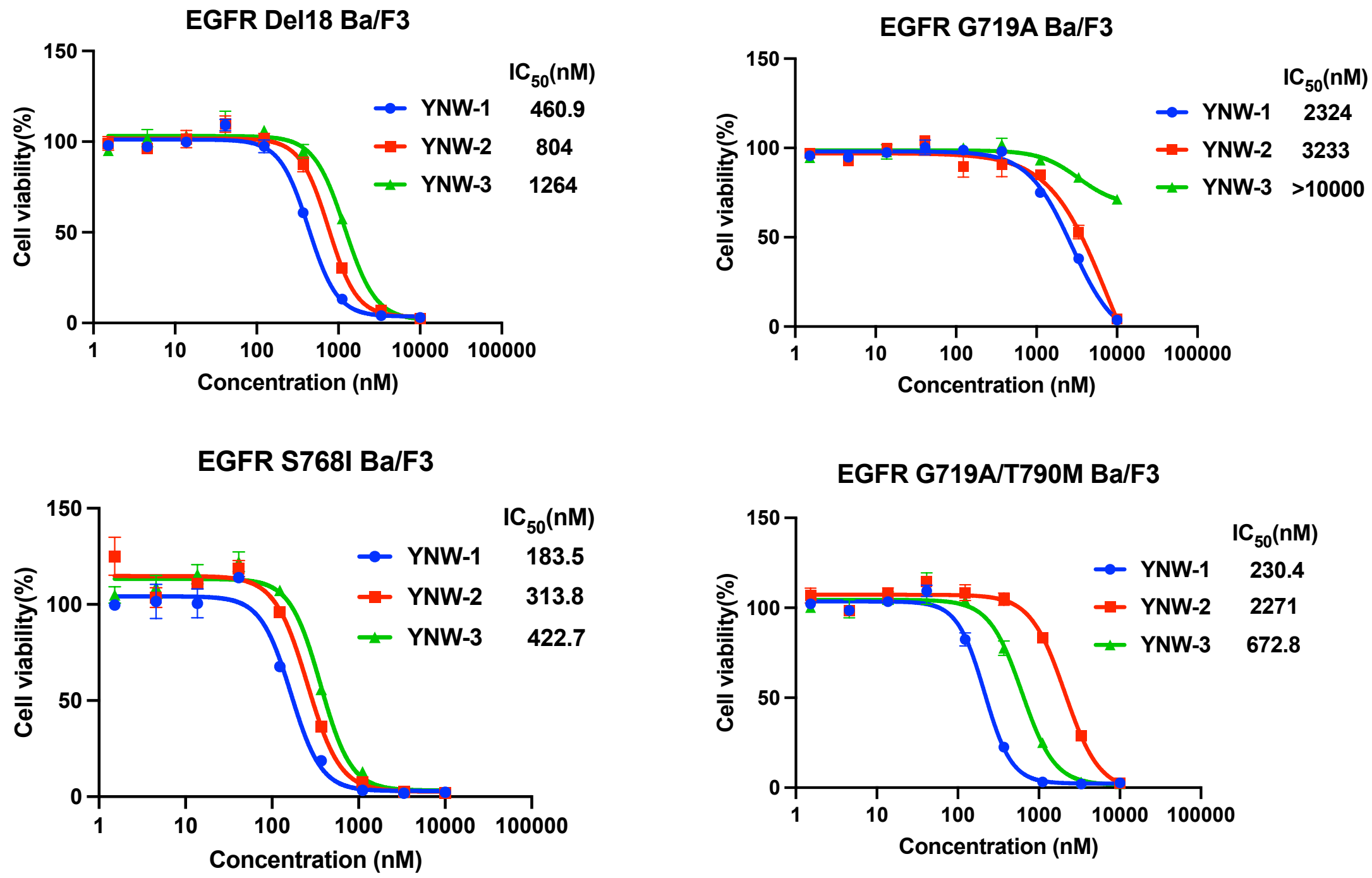


**Figure S4** Dose-response curves for YNW-1, YNW-2 and YNW-3 in the indicated EGFR rare mutant Ba/F3 cells after 72 h treatment. Cell viability was assessed with CellTiter-Glo. Data are presented as the mean±SEM. Representative data from three independent experiments are shown.

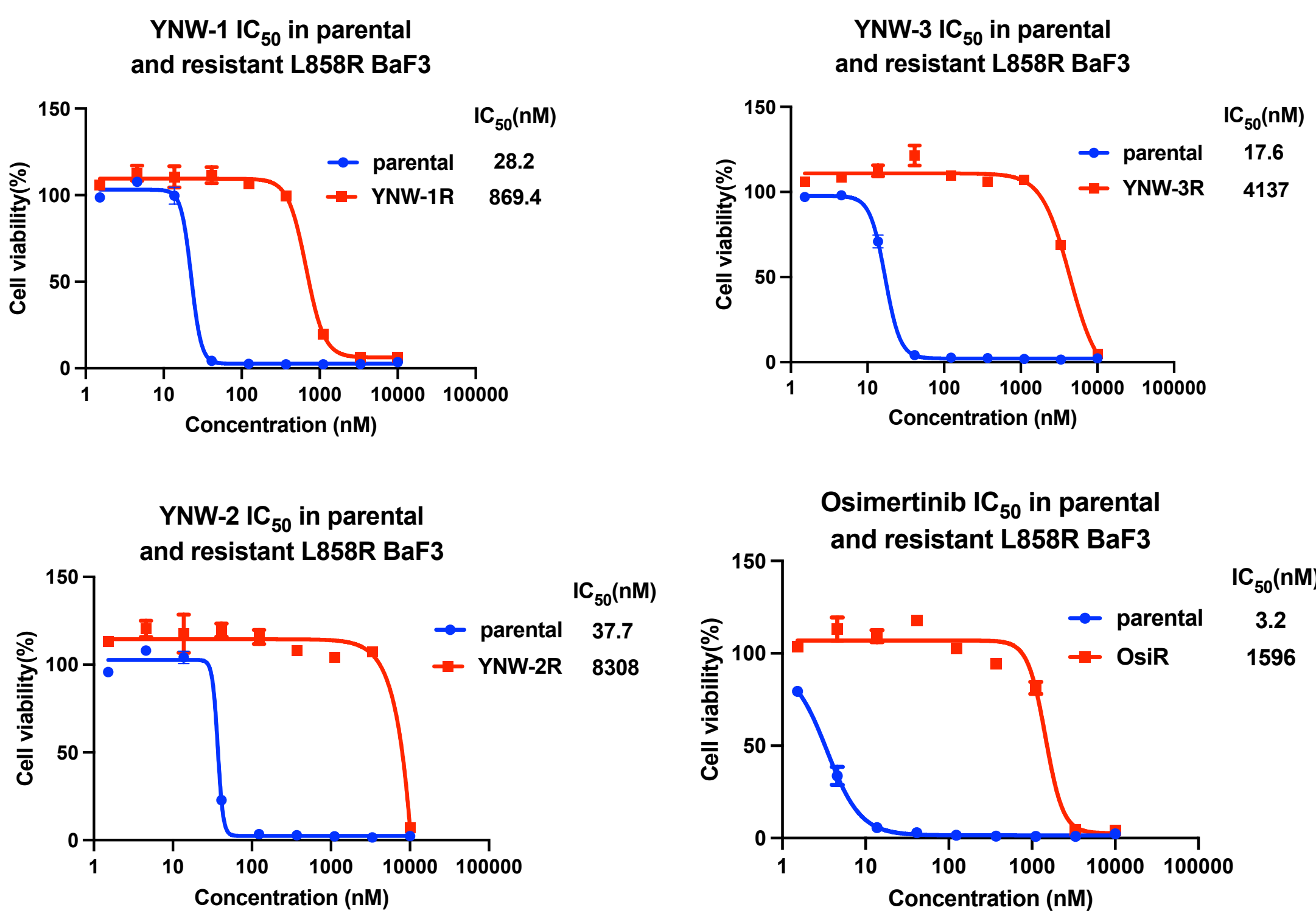

**Figure S5** Dose-response curves for YNW-1, YNW-2, YNW-3 and osimertinib in parental or resistant EGFR L858R Ba/F3 after 72 h treatment. Cell viability was assessed with CellTiter-Glo. Data are presented as the mean±SEM. Representative data from three independent experiments are shown.

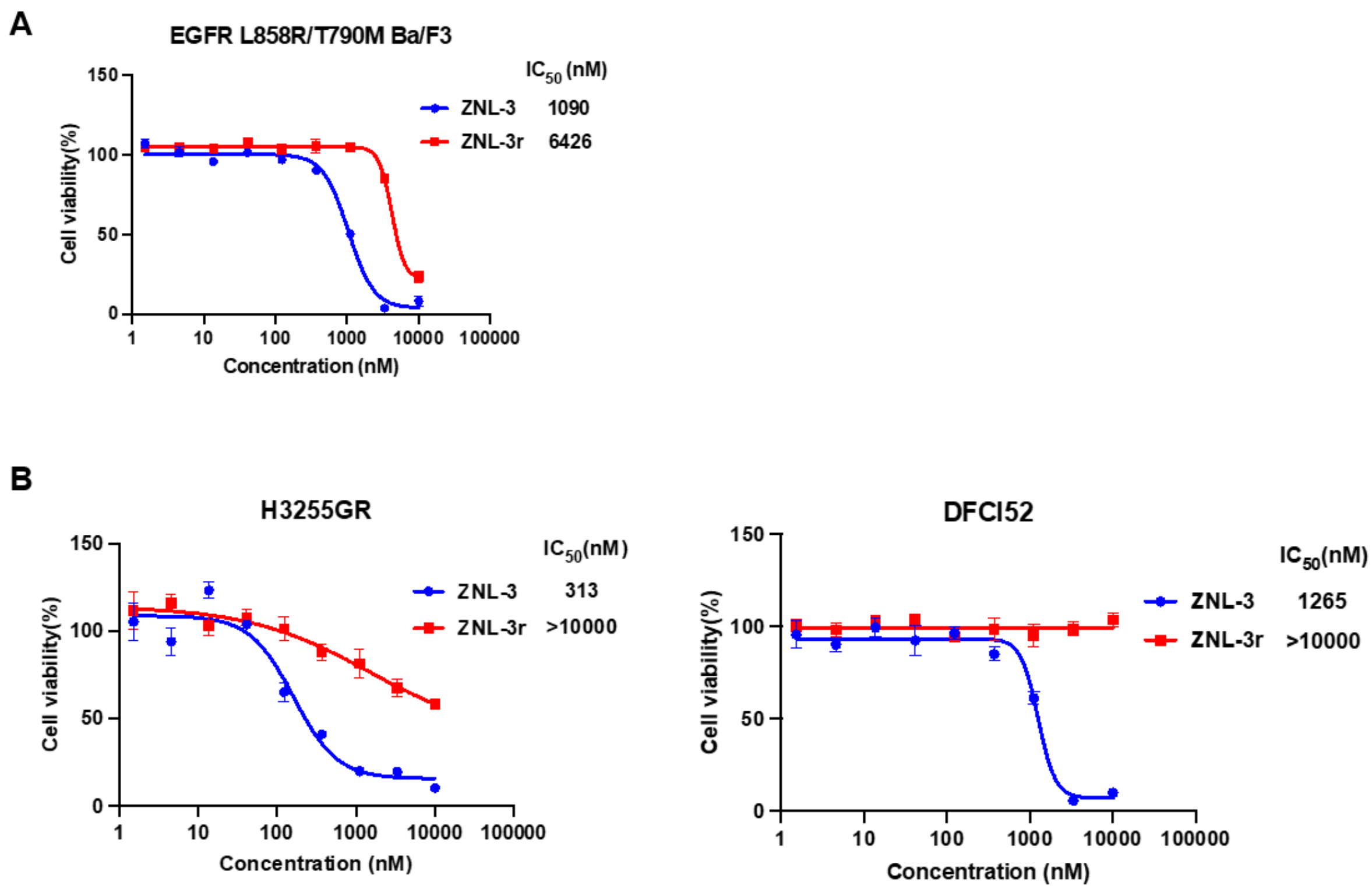


**Figure S6. A.** Dose-response curves for ZNL-3 and ZNL-3r in EGFR L858R/T790M Ba/F3 after 72 h treatment. Cell viability was assessed with CellTiter-Glo. Data are presented as mean±SEM. Representative data from three independent experiments are shown. **B**. Dose-response curves for ZNL-3 and ZNL-3r in H3255GR and DFCI52 after 72 h treatment. Cell viability was assessed with CellTiter-Glo. Data are presented as mean±SEM. Representative data from three independent experiments are shown.

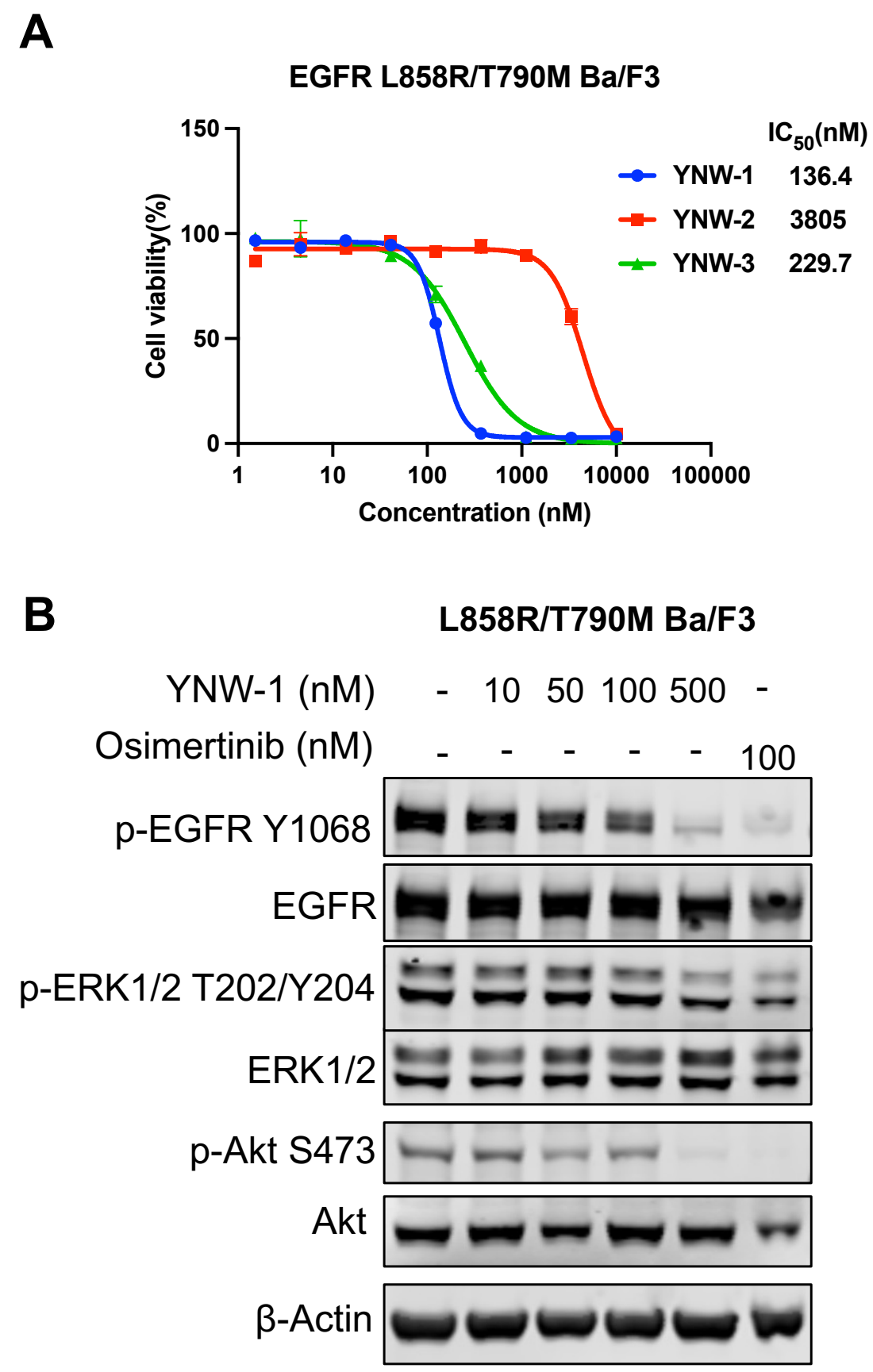


**Figure S7. A.** Dose-response curves for YNW-1, YNW-2, YNW-3 in EGFR L858R/T790M Ba/F3 and EGFR L858R/T790M/C797S Ba/F3 after 72 h treatment. Cell viability was assessed with CellTiter-Glo. Data are presented as mean±SEM. Representative data from three independent experiments are shown. **B**. Effects of YNW-1 on EGFR signaling pathway in EGFR L858R/T790M Ba/F3 after 6 h treatment. Representative data from three independent experiments are shown.

**Table S1.** Data collection and refinement statistics.

| | ZNL-1 |
|---|---|
| **Data Collection** | |
| **PDB Accession** | 9PWI |
| **Resolution range** | 64.54 - 2.6 (2.86 - 2.6) |
| **Space group** | P $4_3$ $2_1$ 2 |
| **Unit cell dimensions (Å)**<br>**Unit cell angles (º)** | 70.39 70.39 161.72<br>90 90 90 |
| **Total reflections** | 84961 (21759) |
| **Unique reflections** | 13148 (3200) |
| **Multiplicity** | 6.5 (6.8) |
| **Completeness (%)** | 99.51 (99.44) |
| **Mean I/sigma(I)** | 9.03 (1.03) |
| **Wilson B-factor** | 71.24 |
| **$R_{merge}$** | 0.0906 (0.8069) |
| **$R_{meas}$** | 0.0989 (0.8739) |
| **$R_{pim}$** | 0.0389 (0.3309) |
| **$CC_{1/2}$** | 0.997 (0.882) |
| **Data Refinement** | |
| **Reflections used in refinement** | 13108 (3183) |
| **Reflections used for R-free** | 605 (141) |
| **$R_{work}$** | 0.224 (0.334) |
| **$R_{free}$** | 0.254 (0.358) |
| **Number of non-hydrogen atoms** | 2243 |
| **macromolecules** | 2202 |
| **ligand** | 38 |
| **solvent** | 3 |
| **Protein residues** | 274 |
| **RMS (bonds)** | 0.177 |
| **RMS (angles)** | 3.18 |
| **Ramachandran favored (%)** | 94.1 |
| **Ramachandran allowed (%)** | 5.5 |
| **Ramachandran outliers (%)** | 0.4 |
| **Rotamer outliers (%)** | 4.5 |
| **Average B-factor** | 84.4 |

| | |
|---|---|
| **macromolecules** | 84.4 |
| **ligands** | 84.1 |

*Statistics for the highest-resolution shell are shown in parentheses.*

**Table S2.** Comparison of *in vitro* metabolic stability for compound ZNL-2S and ZNL-3

| Cmpd ID | Whole blood stability-mouse:T1/2(min) | Whole blood stability-human:T1/2(min) | Hepatocytes Stability- mouse T1/2(min) | Hepatocytes Stability- mouse Clint (hep) (μL/min/10e6) | Hepatocytes Stability- mouse Clint (liver) (μL/min/kg) | Hepatocytes Stability-human T1/2(min) | Hepatocytes Stability- human CLint (hep) (uL/min/10e6) | Hepatocytes Stability- human CLint (live) (uL/min/10e6) |
|---|---|---|---|---|---|---|---|---|
| ZNL-2S | 12.4 | 105 | 3.8 | 363 | 4310 | 24.3 | 56.9 | 158 |
| ZNL-3 | 205 | 214 | 11.2 | 124 | 1470 | 31.2 | 44.5 | 124 |

**Table S3.** Pharmacokinetics in mouse for ZNL-3

| Pharmacokinetics of ZNL-3 (ng/mL) | | | | | | | | | |
|---|---|---|---|---|---|---|---|---|---|
| IV bolus1 | | | | | PO2 | | | | |
| PK Parameters | M01 | M02 | M03 | Mean | PK Parameters | M04 | M05 | M06 | Mean |
| Rsq_adj | 0.996 | 0.996 | 0.988 | -- | Rsq_adj | 0.998 | 0.997 | 0.979 | -- |
| No. points used for $T_{1/2}$ | 3.00 | 3.00 | 3.00 | 3.00 | No. points used for $T_{1/2}$ | 3.00 | 3.00 | 3.00 | 3.00 |
| $C_0$ (ng/mL) | 527 | 519 | 779 | 608 | $C_{max}$ (ng/mL) | 119 | 738 | 186 | 348 |
| $T_{1/2}$ (h) | 1.36 | 1.10 | 0.798 | 1.09 | $T_{max}$ (h) | 0.500 | 1.00 | 0.250 | 0.583 |
| $Vd_{ss}$ (L/kg) | 3.35 | 4.50 | 2.60 | 3.48 | $T_{1/2}$ (h) | 1.42 | 1.49 | 2.05 | 1.65 |
| Cl(mL/min/kg) | 54.7 | 69.8 | 56.7 | 60.4 | $T_{last}$ (h) | 8.00 | 8.00 | 8.00 | 8.00 |
| $T_{last}$ (h) | 8.00 | 4.00 | 4.00 | ND | $AUC_{0\text{-}last}$ (ng.h/mL) | 360 | 1768 | 381 | 836 |
| $AUC_{0\text{-}last}$ (ng.h/mL) | 302 | 227 | 288 | 272 | $AUC_{0\text{-}inf}$ (ng.h/mL) | 370 | 1813 | 407 | 863 |
| $AUC_{0\text{-}inf}$ (ng.h/mL) | 305 | 239 | 294 | 279 | $MRT_{0\text{-}last}$ (h) | 2.42 | 2.02 | 2.21 | 2.22 |
| $MRT_{0\text{-}last}$ (h) | 0.954 | 0.842 | 0.677 | 0.824 | $MRT_{0\text{-}inf}$ (h) | 2.62 | 2.22 | 2.77 | 2.54 |
| $MRT_{0\text{-}inf}$ (h) | 1.02 | 1.08 | 0.764 | 0.955 | $AUC_{Extra}$ (%) | 2.70 | 2.49 | 6.37 | 3.85 |
| $AUC_{Extra}$ (%) | 0.727 | 4.93 | 1.95 | 2.54 | $AUMC_{Extra}$ (%) | 10.4 | 11.4 | 25.2 | 15.7 |
| $AUMC_{Extra}$ (%) | 7.10 | 25.6 | 13.2 | 15.3 | Bioavailability (%)[a] | - | - | - | 103 |

Note: Mouse #5 was treated as an outlier and bioavailability was re-calculated

**Table S4. ZNL-8** DiscoverX KINOME*scan* at 1 μM

| Kinase Targets | Percent Control | Compound Concentration (μM) |
|---|---|---|
| KIT | 0.2 | 1 |
| KIT(V559D) | 0.2 | 1 |
| EGFR(L861Q) | 0.35 | 1 |
| FLT4 | 0.35 | 1 |
| PDGFRB | 0.35 | 1 |
| EGFR(G719S) | 0.45 | 1 |

| | | |
|---|---|---|
| ABL1(Q252H)-nonphosphorylated | 0.6 | 1 |
| EGFR(G719C) | 0.65 | 1 |
| MAP3K4 | 0.7 | 1 |
| EGFR(L858R) | 0.75 | 1 |
| CDKL2 | 0.85 | 1 |
| VEGFR2 | 0.95 | 1 |
| ABL1(H396P)-nonphosphorylated | 1.2 | 1 |
| LOK | 1.3 | 1 |
| KIT(L576P) | 1.6 | 1 |
| RET | 2.3 | 1 |
| FLT1 | 2.5 | 1 |
| TIE2 | 2.5 | 1 |
| DDR1 | 2.8 | 1 |
| EGFR | 2.9 | 1 |
| EGFR(E746-A750del) | 3.1 | 1 |
| ABL1-nonphosphorylated | 3.2 | 1 |
| FGFR1 | 3.2 | 1 |
| EGFR(S752-I759del) | 3.7 | 1 |
| KIT(D816V) | 4.3 | 1 |
| EGFR(L747-E749del, A750P) | 4.7 | 1 |
| EGFR(L747-S752del, P753S) | 5.5 | 1 |
| TIE1 | 5.5 | 1 |
| EGFR(L747-T751del,Sins) | 5.6 | 1 |
| ABL1(Q252H)-phosphorylated | 5.9 | 1 |
| ABL1-phosphorylated | 6 | 1 |
| FGFR2 | 6.1 | 1 |
| NEK2 | 6.1 | 1 |
| RET(M918T) | 6.5 | 1 |
| ABL1(E255K)-phosphorylated | 6.8 | 1 |
| FGFR3 | 6.9 | 1 |
| ABL1(Y253F)-phosphorylated | 7 | 1 |
| LCK | 7.1 | 1 |
| ABL1(M351T)-phosphorylated | 7.5 | 1 |
| EPHB6 | 7.6 | 1 |

| | | |
|---|---|---|
| ABL1(H396P)-phosphorylated | 8.6 | 1 |
| FGFR3(G697C) | 8.6 | 1 |
| YSK4 | 8.9 | 1 |
| BLK | 9 | 1 |
| FGFR4 | 9.6 | 1 |
| ACVR1B | 9.7 | 1 |
| KIT(V559D,V654A) | 9.9 | 1 |
| TGFBR1 | 11 | 1 |
| JNK2 | 12 | 1 |
| GCN2(Kin.Dom.2,S808G) | 14 | 1 |
| KIT(A829P) | 14 | 1 |
| BRAF(V600E) | 15 | 1 |
| ERBB3 | 16 | 1 |
| KIT(D816H) | 16 | 1 |
| TAOK2 | 17 | 1 |
| GAK | 18 | 1 |
| EPHA1 | 20 | 1 |
| ZAK | 21 | 1 |
| MAP4K5 | 22 | 1 |
| RIPK2 | 22 | 1 |
| CSF1R | 23 | 1 |
| CSK | 23 | 1 |
| PDGFRA | 26 | 1 |
| RIOK1 | 26 | 1 |
| YES | 26 | 1 |
| FGR | 30 | 1 |
| LIMK2 | 30 | 1 |
| BRAF | 31 | 1 |
| MEK5 | 32 | 1 |
| TAOK3 | 33 | 1 |
| BMX | 34 | 1 |
| HPK1 | 34 | 1 |
| LIMK1 | 35 | 1 |
| PFCDPK1(P.falciparum) | 36 | 1 |
| MLK2 | 37 | 1 |
| STK36 | 37 | 1 |

| | | |
|---|---|---|
| TAOK1 | 39 | 1 |
| DDR2 | 41 | 1 |
| TESK1 | 41 | 1 |
| ERBB4 | 43 | 1 |
| SLK | 44 | 1 |
| ABL2 | 45 | 1 |
| EGFR(L858R,T790M) | 45 | 1 |
| MAP3K3 | 45 | 1 |
| CSNK1G1 | 47 | 1 |
| FLT3(ITD,F691L) | 47 | 1 |
| LYN | 47 | 1 |
| MAP4K3 | 48 | 1 |
| MINK | 49 | 1 |
| HCK | 50 | 1 |
| p38-alpha | 51 | 1 |
| TNK2 | 52 | 1 |
| DLK | 55 | 1 |
| PIK3C2B | 57 | 1 |
| MAST1 | 59 | 1 |
| EPHA8 | 60 | 1 |
| FRK | 61 | 1 |
| ABL1(F317L)-nonphosphorylated | 62 | 1 |
| FES | 62 | 1 |
| PIK3CA(E545A) | 62 | 1 |
| SIK2 | 62 | 1 |
| LZK | 65 | 1 |
| MAP4K2 | 65 | 1 |
| PIP5K2C | 65 | 1 |
| SRC | 65 | 1 |
| BRK | 66 | 1 |
| TYRO3 | 66 | 1 |
| MEK2 | 68 | 1 |
| SRMS | 68 | 1 |
| CDKL3 | 69 | 1 |
| MET | 69 | 1 |

| | | |
|---|---|---|
| MLK3 | 69 | 1 |
| MTOR | 69 | 1 |
| PIK3C2G | 69 | 1 |
| RAF1 | 69 | 1 |
| STK39 | 69 | 1 |
| PAK3 | 71 | 1 |
| NLK | 72 | 1 |
| TRKA | 72 | 1 |
| BMPR1B | 73 | 1 |
| PIK3CD | 73 | 1 |
| TNK1 | 74 | 1 |
| CAMK1 | 75 | 1 |
| PIK3CA(M1043I) | 75 | 1 |
| PRKD1 | 75 | 1 |
| ABL1(F317L)-phosphorylated | 76 | 1 |
| FLT3(D835V) | 76 | 1 |
| FYN | 76 | 1 |
| JNK3 | 76 | 1 |
| RIOK3 | 76 | 1 |
| TNIK | 76 | 1 |
| TNNI3K | 76 | 1 |
| CAMK1B | 77 | 1 |
| MAP3K2 | 77 | 1 |
| PRKCE | 77 | 1 |
| HASPIN | 78 | 1 |
| KIT(V559D,T670I) | 78 | 1 |
| KIT-autoinhibited | 78 | 1 |
| MLK1 | 78 | 1 |
| PIK3CA | 78 | 1 |
| ANKK1 | 79 | 1 |
| DRAK1 | 79 | 1 |
| JNK1 | 79 | 1 |
| PAK1 | 79 | 1 |
| PRKD3 | 79 | 1 |
| FLT3(R834Q) | 80 | 1 |
| NEK5 | 80 | 1 |

| | | |
|---|---|---|
| SgK110 | 80 | 1 |
| TTK | 80 | 1 |
| DMPK2 | 81 | 1 |
| LKB1 | 81 | 1 |
| MKK7 | 81 | 1 |
| SYK | 81 | 1 |
| ABL1(T315I)-phosphorylated | 82 | 1 |
| AMPK-alpha2 | 82 | 1 |
| EPHA2 | 82 | 1 |
| ERBB2 | 82 | 1 |
| FER | 82 | 1 |
| PCTK1 | 82 | 1 |
| RET(V804L) | 82 | 1 |
| SIK | 82 | 1 |
| CSNK2A1 | 83 | 1 |
| DRAK2 | 83 | 1 |
| MAP4K4 | 83 | 1 |
| RIPK1 | 83 | 1 |
| SBK1 | 83 | 1 |
| SGK2 | 83 | 1 |
| SNARK | 83 | 1 |
| BIKE | 84 | 1 |
| EPHB1 | 84 | 1 |
| ERK4 | 84 | 1 |
| FLT3(K663Q) | 84 | 1 |
| MEK3 | 84 | 1 |
| PIK3CA(Q546K) | 84 | 1 |
| PRKR | 84 | 1 |
| RET(V804M) | 84 | 1 |
| BMPR1A | 85 | 1 |
| CDK11 | 85 | 1 |
| p38-beta | 85 | 1 |
| PIK3CA(H1047L) | 85 | 1 |
| RPS6KA4(Kin.Dom.1-N-terminal) | 85 | 1 |
| RSK2(Kin.Dom.2-C-terminal) | 85 | 1 |

| | | |
|---|---|---|
| TSSK1B | 85 | 1 |
| HUNK | 86 | 1 |
| MEK1 | 86 | 1 |
| OSR1 | 86 | 1 |
| PIK3CA(H1047Y) | 86 | 1 |
| PIKFYVE | 86 | 1 |
| PKAC-alpha | 86 | 1 |
| TRKC | 86 | 1 |
| TXK | 86 | 1 |
| ABL1(T315I)-nonphosphorylated | 87 | 1 |
| CDC2L5 | 87 | 1 |
| MKNK2 | 87 | 1 |
| PIK3CA(E545K) | 87 | 1 |
| RPS6KA4(Kin.Dom.2-C-terminal) | 87 | 1 |
| SGK | 87 | 1 |
| AURKC | 88 | 1 |
| BMPR2 | 88 | 1 |
| CAMK2A | 88 | 1 |
| FLT3(D835H) | 88 | 1 |
| GRK3 | 88 | 1 |
| MAP3K15 | 88 | 1 |
| MRCKA | 88 | 1 |
| PIK3CA(E542K) | 88 | 1 |
| WNK4 | 88 | 1 |
| ACVR1 | 89 | 1 |
| AKT3 | 89 | 1 |
| CTK | 89 | 1 |
| DYRK1A | 89 | 1 |
| ERK3 | 89 | 1 |
| IRAK3 | 89 | 1 |
| LATS1 | 89 | 1 |
| MERTK | 89 | 1 |
| MYLK4 | 89 | 1 |
| p38-gamma | 89 | 1 |

| | | |
|---|---|---|
| PCTK2 | 89 | 1 |
| PIK3CA(C420R) | 89 | 1 |
| PIK3CG | 89 | 1 |
| SGK3 | 89 | 1 |
| SRPK3 | 89 | 1 |
| TEC | 89 | 1 |
| ASK2 | 90 | 1 |
| BUB1 | 90 | 1 |
| CDK4-cyclinD3 | 90 | 1 |
| CHEK2 | 90 | 1 |
| DCAMKL2 | 90 | 1 |
| DYRK1B | 90 | 1 |
| ERK5 | 90 | 1 |
| IRAK1 | 90 | 1 |
| MYLK2 | 90 | 1 |
| MYO3B | 90 | 1 |
| ROCK1 | 90 | 1 |
| RPS6KA5(Kin.Dom.2-C-terminal) | 90 | 1 |
| RSK4(Kin.Dom.1-N-terminal) | 90 | 1 |
| YANK2 | 90 | 1 |
| ADCK3 | 91 | 1 |
| CAMK1D | 91 | 1 |
| CDC2L1 | 91 | 1 |
| CDKL1 | 91 | 1 |
| CLK2 | 91 | 1 |
| EPHA3 | 91 | 1 |
| EPHB4 | 91 | 1 |
| MARK2 | 91 | 1 |
| MYLK | 91 | 1 |
| PIK3CA(I800L) | 91 | 1 |
| PIK4CB | 91 | 1 |
| RIOK2 | 91 | 1 |
| TGFBR2 | 91 | 1 |
| TLK2 | 91 | 1 |
| CLK3 | 92 | 1 |

| | | |
|---|---|---|
| FLT3(ITD,D835V) | 92 | 1 |
| GSK3B | 92 | 1 |
| ICK | 92 | 1 |
| LATS2 | 92 | 1 |
| MAPKAPK5 | 92 | 1 |
| MLCK | 92 | 1 |
| PIP5K1A | 92 | 1 |
| CAMK2D | 93 | 1 |
| CIT | 93 | 1 |
| EGFR(T790M) | 93 | 1 |
| GRK4 | 93 | 1 |
| MAK | 93 | 1 |
| MARK1 | 93 | 1 |
| MET(Y1235D) | 93 | 1 |
| MST4 | 93 | 1 |
| NEK4 | 93 | 1 |
| PRKCD | 93 | 1 |
| PRKG1 | 93 | 1 |
| TRPM6 | 93 | 1 |
| ULK3 | 93 | 1 |
| VPS34 | 93 | 1 |
| ACVRL1 | 94 | 1 |
| BRSK1 | 94 | 1 |
| CAMK2B | 94 | 1 |
| CDK9 | 94 | 1 |
| CSF1R-autoinhibited | 94 | 1 |
| CSNK1A1 | 94 | 1 |
| FLT3(D835Y) | 94 | 1 |
| HIPK2 | 94 | 1 |
| HIPK3 | 94 | 1 |
| MARK3 | 94 | 1 |
| PAK2 | 94 | 1 |
| PFPK5(P.falciparum) | 94 | 1 |
| PKN2 | 94 | 1 |
| ROS1 | 94 | 1 |
| S6K1 | 94 | 1 |

| | | |
|---|---|---|
| VRK2 | 94 | 1 |
| YANK1 | 94 | 1 |
| AAK1 | 95 | 1 |
| AKT1 | 95 | 1 |
| AXL | 95 | 1 |
| CAMK1G | 95 | 1 |
| CAMK4 | 95 | 1 |
| CDK5 | 95 | 1 |
| CSNK1E | 95 | 1 |
| EPHA6 | 95 | 1 |
| EPHA7 | 95 | 1 |
| FLT3-autoinhibited | 95 | 1 |
| MKNK1 | 95 | 1 |
| NDR2 | 95 | 1 |
| p38-delta | 95 | 1 |
| PAK6 | 95 | 1 |
| PAK7 | 95 | 1 |
| PIP5K2B | 95 | 1 |
| PKN1 | 95 | 1 |
| RPS6KA5(Kin.Dom.1-N-terminal) | 95 | 1 |
| TBK1 | 95 | 1 |
| ADCK4 | 96 | 1 |
| DAPK2 | 96 | 1 |
| EPHA5 | 96 | 1 |
| FAK | 96 | 1 |
| HIPK1 | 96 | 1 |
| ITK | 96 | 1 |
| MAP3K1 | 96 | 1 |
| NEK10 | 96 | 1 |
| PFTAIRE2 | 96 | 1 |
| PLK4 | 96 | 1 |
| PRKX | 96 | 1 |
| TYK2(JH2domain-pseudokinase) | 96 | 1 |
| ULK1 | 96 | 1 |

| | | |
|---|---|---|
| ZAP70 | 96 | 1 |
| CDK2 | 97 | 1 |
| CHEK1 | 97 | 1 |
| CSNK1D | 97 | 1 |
| EIF2AK1 | 97 | 1 |
| ERK2 | 97 | 1 |
| FLT3(N841I) | 97 | 1 |
| HIPK4 | 97 | 1 |
| INSRR | 97 | 1 |
| JAK2(JH1domain-catalytic) | 97 | 1 |
| MAPKAPK2 | 97 | 1 |
| MEK4 | 97 | 1 |
| MST1 | 97 | 1 |
| MST1R | 97 | 1 |
| NEK6 | 97 | 1 |
| NIM1 | 97 | 1 |
| PAK4 | 97 | 1 |
| PDPK1 | 97 | 1 |
| PIK3CB | 97 | 1 |
| PIP5K1C | 97 | 1 |
| QSK | 97 | 1 |
| RSK1(Kin.Dom.1-N-terminal) | 97 | 1 |
| TSSK3 | 97 | 1 |
| TYK2(JH1domain-catalytic) | 97 | 1 |
| YANK3 | 97 | 1 |
| AMPK-alpha1 | 98 | 1 |
| CASK | 98 | 1 |
| CDKL5 | 98 | 1 |
| EPHA4 | 98 | 1 |
| EPHB3 | 98 | 1 |
| ERN1 | 98 | 1 |
| GRK2 | 98 | 1 |
| IRAK4 | 98 | 1 |
| MET(M1250T) | 98 | 1 |
| PFTK1 | 98 | 1 |
| PIM2 | 98 | 1 |

| | | |
|---|---|---|
| PRKG2 | 98 | 1 |
| AKT2 | 99 | 1 |
| ALK | 99 | 1 |
| ALK(L1196M) | 99 | 1 |
| BRSK2 | 99 | 1 |
| CAMK2G | 99 | 1 |
| DAPK3 | 99 | 1 |
| DMPK | 99 | 1 |
| EPHB2 | 99 | 1 |
| FLT3(ITD) | 99 | 1 |
| JAK1(JH2domain-pseudokinase) | 99 | 1 |
| LTK | 99 | 1 |
| MARK4 | 99 | 1 |
| NIK | 99 | 1 |
| RIPK4 | 99 | 1 |
| ROCK2 | 99 | 1 |
| SRPK2 | 99 | 1 |
| STK16 | 99 | 1 |
| WEE2 | 99 | 1 |
| ABL1(F317I)-nonphosphorylated | 100 | 1 |
| ABL1(F317I)-phosphorylated | 100 | 1 |
| ACVR2A | 100 | 1 |
| ACVR2B | 100 | 1 |
| ALK(C1156Y) | 100 | 1 |
| ARK5 | 100 | 1 |
| ASK1 | 100 | 1 |
| AURKA | 100 | 1 |
| AURKB | 100 | 1 |
| BTK | 100 | 1 |
| CAMKK1 | 100 | 1 |
| CAMKK2 | 100 | 1 |
| CDC2L2 | 100 | 1 |
| CDK3 | 100 | 1 |
| CDK4 | 100 | 1 |

| | | |
|---|---|---|
| CDK4-cyclinD1 | 100 | 1 |
| CDK7 | 100 | 1 |
| CDK8 | 100 | 1 |
| CLK1 | 100 | 1 |
| CLK4 | 100 | 1 |
| CSNK1A1L | 100 | 1 |
| CSNK1G2 | 100 | 1 |
| CSNK1G3 | 100 | 1 |
| CSNK2A2 | 100 | 1 |
| DAPK1 | 100 | 1 |
| DCAMKL1 | 100 | 1 |
| DCAMKL3 | 100 | 1 |
| DYRK2 | 100 | 1 |
| ERK1 | 100 | 1 |
| ERK8 | 100 | 1 |
| FLT3 | 100 | 1 |
| GRK1 | 100 | 1 |
| GRK7 | 100 | 1 |
| GSK3A | 100 | 1 |
| IGF1R | 100 | 1 |
| IKK-alpha | 100 | 1 |
| IKK-beta | 100 | 1 |
| IKK-epsilon | 100 | 1 |
| INSR | 100 | 1 |
| JAK1(JH1domain-catalytic) | 100 | 1 |
| JAK3(JH1domain-catalytic) | 100 | 1 |
| LRRK2 | 100 | 1 |
| LRRK2(G2019S) | 100 | 1 |
| MEK6 | 100 | 1 |
| MELK | 100 | 1 |
| MRCKB | 100 | 1 |
| MST2 | 100 | 1 |
| MST3 | 100 | 1 |
| MUSK | 100 | 1 |
| MYO3A | 100 | 1 |
| NDR1 | 100 | 1 |

| | | |
|---|---|---|
| NEK1 | 100 | 1 |
| NEK11 | 100 | 1 |
| NEK3 | 100 | 1 |
| NEK7 | 100 | 1 |
| NEK9 | 100 | 1 |
| PCTK3 | 100 | 1 |
| PHKG1 | 100 | 1 |
| PHKG2 | 100 | 1 |
| PIM1 | 100 | 1 |
| PIM3 | 100 | 1 |
| PKAC-beta | 100 | 1 |
| PKMYT1 | 100 | 1 |
| PKNB(M.tuberculosis) | 100 | 1 |
| PLK1 | 100 | 1 |
| PLK2 | 100 | 1 |
| PLK3 | 100 | 1 |
| PRKCH | 100 | 1 |
| PRKCI | 100 | 1 |
| PRKCQ | 100 | 1 |
| PRKD2 | 100 | 1 |
| PRP4 | 100 | 1 |
| PYK2 | 100 | 1 |
| RIPK5 | 100 | 1 |
| RSK1(Kin.Dom.2-C-terminal) | 100 | 1 |
| RSK2(Kin.Dom.1-N-terminal) | 100 | 1 |
| RSK3(Kin.Dom.1-N-terminal) | 100 | 1 |
| RSK3(Kin.Dom.2-C-terminal) | 100 | 1 |
| RSK4(Kin.Dom.2-C-terminal) | 100 | 1 |
| SNRK | 100 | 1 |
| SRPK1 | 100 | 1 |
| STK33 | 100 | 1 |
| STK35 | 100 | 1 |
| TAK1 | 100 | 1 |
| TLK1 | 100 | 1 |
| TRKB | 100 | 1 |
| ULK2 | 100 | 1 |

| | | |
|---|---|---|
| WEE1 | 100 | 1 |
| WNK1 | 100 | 1 |
| WNK2 | 100 | 1 |
| WNK3 | 100 | 1 |
| YSK1 | 100 | 1 |

**Table S5. YNW-1** DiscoverX KINOME*scan* at 1 μM

| **Kinase Targets** | **Percent Control** | **Compound Concentration (μM)** |
|---|---|---|
| EGFR(L858R) | 0.7 | 1 |
| EGFR(G719C) | 1.1 | 1 |
| EGFR(L861Q) | 1.6 | 1 |
| p38-alpha | 1.8 | 1 |
| EGFR(L747-E749del, A750P) | 2.1 | 1 |
| EGFR | 2.7 | 1 |
| JNK2 | 2.9 | 1 |
| TIE2 | 3.2 | 1 |
| EGFR(G719S) | 3.8 | 1 |
| EPHB6 | 3.9 | 1 |
| EGFR(S752-I759del) | 5.3 | 1 |
| EGFR(T790M) | 5.7 | 1 |
| EGFR(L747-T751del,Sins) | 6 | 1 |
| FLT4 | 6 | 1 |
| ERBB4 | 6.9 | 1 |
| ERBB2 | 7 | 1 |
| EGFR(L858R,T790M) | 7.3 | 1 |
| EGFR(L747-S752del, P753S) | 14 | 1 |
| EGFR(E746-A750del) | 18 | 1 |
| VEGFR2 | 21 | 1 |
| TNK2 | 22 | 1 |
| FGFR4 | 30 | 1 |
| FGFR1 | 40 | 1 |
| FLT1 | 46 | 1 |
| SRMS | 49 | 1 |
| BMPR1A | 53 | 1 |

| | | |
|---|---|---|
| KIT(A829P) | 53 | 1 |
| BLK | 56 | 1 |
| FGFR2 | 57 | 1 |
| PRP4 | 57 | 1 |
| GRK4 | 59 | 1 |
| KIT(V559D) | 59 | 1 |
| KIT(L576P) | 60 | 1 |
| ABL1-phosphorylated | 65 | 1 |
| KIT | 66 | 1 |
| KIT(D816V) | 66 | 1 |
| MARK1 | 66 | 1 |
| TAOK2 | 66 | 1 |
| CSNK1E | 69 | 1 |
| CSNK2A1 | 69 | 1 |
| FER | 69 | 1 |
| FGFR3 | 70 | 1 |
| STK39 | 70 | 1 |
| FES | 71 | 1 |
| FLT3(D835H) | 71 | 1 |
| TIE1 | 71 | 1 |
| JNK3 | 72 | 1 |
| MAP3K15 | 72 | 1 |
| PIK3CA(H1047L) | 72 | 1 |
| CTK | 73 | 1 |
| PIK3CA(E542K) | 73 | 1 |
| TYRO3 | 73 | 1 |
| CLK4 | 74 | 1 |
| PIK3CA(Q546K) | 74 | 1 |
| KIT(D816H) | 75 | 1 |
| MEK2 | 75 | 1 |
| RET | 75 | 1 |
| ABL1(E255K)-phosphorylated | 76 | 1 |
| ACVR1B | 76 | 1 |
| LATS2 | 76 | 1 |
| MAST1 | 76 | 1 |
| SRPK2 | 76 | 1 |

| | | |
|---|---|---|
| ABL1(F317L)-phosphorylated | 77 | 1 |
| ABL1(H396P)-phosphorylated | 77 | 1 |
| BMX | 77 | 1 |
| DRAK1 | 77 | 1 |
| DYRK1B | 77 | 1 |
| MKNK1 | 77 | 1 |
| CSF1R | 78 | 1 |
| HUNK | 78 | 1 |
| LIMK1 | 78 | 1 |
| PIK3CA | 78 | 1 |
| MEK3 | 79 | 1 |
| NDR1 | 79 | 1 |
| PRKCH | 79 | 1 |
| YANK2 | 79 | 1 |
| EPHA2 | 80 | 1 |
| FLT3 | 80 | 1 |
| JAK1(JH1domain-catalytic) | 81 | 1 |
| PIK3CA(E545K) | 81 | 1 |
| RPS6KA5(Kin.Dom.2-C-terminal) | 81 | 1 |
| BTK | 82 | 1 |
| CASK | 82 | 1 |
| DDR2 | 82 | 1 |
| FRK | 82 | 1 |
| JNK1 | 82 | 1 |
| LIMK2 | 82 | 1 |
| RET(V804M) | 82 | 1 |
| ARK5 | 83 | 1 |
| BRSK2 | 83 | 1 |
| CLK1 | 83 | 1 |
| GRK7 | 83 | 1 |
| ICK | 83 | 1 |
| MAPKAPK2 | 83 | 1 |
| NIK | 83 | 1 |
| p38-beta | 83 | 1 |
| PFPK5(P.falciparum) | 83 | 1 |

| | | |
|---|---|---|
| ULK1 | 83 | 1 |
| ABL1(F317L)-nonphosphorylated | 84 | 1 |
| AMPK-alpha2 | 84 | 1 |
| AURKB | 84 | 1 |
| CDK4 | 84 | 1 |
| CSF1R-autoinhibited | 84 | 1 |
| DDR1 | 84 | 1 |
| DMPK2 | 84 | 1 |
| EPHA3 | 84 | 1 |
| LOK | 84 | 1 |
| MST2 | 84 | 1 |
| STK33 | 84 | 1 |
| TNK1 | 84 | 1 |
| ABL1(H396P)-nonphosphorylated | 85 | 1 |
| DYRK2 | 85 | 1 |
| EPHA4 | 85 | 1 |
| GAK | 85 | 1 |
| GSK3A | 85 | 1 |
| MERTK | 85 | 1 |
| DAPK2 | 86 | 1 |
| FLT3(ITD,D835V) | 86 | 1 |
| JAK2(JH1domain-catalytic) | 86 | 1 |
| MEK5 | 86 | 1 |
| MELK | 86 | 1 |
| MINK | 86 | 1 |
| MYLK2 | 86 | 1 |
| NLK | 86 | 1 |
| ROCK2 | 86 | 1 |
| SNRK | 86 | 1 |
| ABL1(F317I)-nonphosphorylated | 87 | 1 |
| ABL1-nonphosphorylated | 87 | 1 |
| CDKL2 | 87 | 1 |
| LTK | 87 | 1 |

| | | |
|---|---|---|
| PIK3C2G | 87 | 1 |
| PRKD2 | 87 | 1 |
| PYK2 | 87 | 1 |
| RPS6KA5(Kin.Dom.1-N-terminal) | 87 | 1 |
| YSK1 | 87 | 1 |
| ABL1(F317I)-phosphorylated | 88 | 1 |
| ABL1(Y253F)-phosphorylated | 88 | 1 |
| CDK8 | 88 | 1 |
| CDKL5 | 88 | 1 |
| CSNK1D | 88 | 1 |
| FLT3(D835Y) | 88 | 1 |
| MARK4 | 88 | 1 |
| PAK2 | 88 | 1 |
| TNIK | 88 | 1 |
| VRK2 | 88 | 1 |
| BRAF(V600E) | 89 | 1 |
| CDKL1 | 89 | 1 |
| EPHA6 | 89 | 1 |
| MLK2 | 89 | 1 |
| OSR1 | 89 | 1 |
| PKNB(M.tuberculosis) | 89 | 1 |
| PRKCI | 89 | 1 |
| RIOK2 | 89 | 1 |
| SIK2 | 89 | 1 |
| ABL1(Q252H)-nonphosphorylated | 90 | 1 |
| CAMK2B | 90 | 1 |
| DCAMKL1 | 90 | 1 |
| FGFR3(G697C) | 90 | 1 |
| GRK3 | 90 | 1 |
| HIPK4 | 90 | 1 |
| MST4 | 90 | 1 |
| NEK2 | 90 | 1 |
| PDGFRB | 90 | 1 |
| PDPK1 | 90 | 1 |

| | | |
|---|---|---|
| QSK | 90 | 1 |
| RIPK1 | 90 | 1 |
| RIPK5 | 90 | 1 |
| SRPK1 | 90 | 1 |
| TAK1 | 90 | 1 |
| TEC | 90 | 1 |
| ASK2 | 91 | 1 |
| ERBB3 | 91 | 1 |
| MET(M1250T) | 91 | 1 |
| PHKG2 | 91 | 1 |
| RET(V804L) | 91 | 1 |
| RIPK4 | 91 | 1 |
| RSK1(Kin.Dom.2-C-terminal) | 91 | 1 |
| ABL1(Q252H)-phosphorylated | 92 | 1 |
| AURKC | 92 | 1 |
| AXL | 92 | 1 |
| BRK | 92 | 1 |
| CDK4-cyclinD1 | 92 | 1 |
| CHEK1 | 92 | 1 |
| CSNK1G2 | 92 | 1 |
| ERK1 | 92 | 1 |
| ERK8 | 92 | 1 |
| ERN1 | 92 | 1 |
| GRK1 | 92 | 1 |
| KIT(V559D,T670I) | 92 | 1 |
| LCK | 92 | 1 |
| LZK | 92 | 1 |
| NEK4 | 92 | 1 |
| PAK7 | 92 | 1 |
| PFTAIRE2 | 92 | 1 |
| PIK3C2B | 92 | 1 |
| PIK3CA(H1047Y) | 92 | 1 |
| PIK3CG | 92 | 1 |
| PKMYT1 | 92 | 1 |
| ROS1 | 92 | 1 |
| SLK | 92 | 1 |

| | | |
|---|---|---|
| SRC | 92 | 1 |
| TGFBR1 | 92 | 1 |
| ADCK3 | 93 | 1 |
| ASK1 | 93 | 1 |
| CLK3 | 93 | 1 |
| DAPK1 | 93 | 1 |
| HASPIN | 93 | 1 |
| INSR | 93 | 1 |
| MAP3K3 | 93 | 1 |
| MEK1 | 93 | 1 |
| NDR2 | 93 | 1 |
| NEK5 | 93 | 1 |
| NEK7 | 93 | 1 |
| PFCDPK1(P.falciparum) | 93 | 1 |
| PIK3CB | 93 | 1 |
| RET(M918T) | 93 | 1 |
| TAOK1 | 93 | 1 |
| TNNI3K | 93 | 1 |
| WNK3 | 93 | 1 |
| YES | 93 | 1 |
| ACVR2A | 94 | 1 |
| CDC2L5 | 94 | 1 |
| CDK2 | 94 | 1 |
| DAPK3 | 94 | 1 |
| EPHA1 | 94 | 1 |
| EPHA7 | 94 | 1 |
| EPHA8 | 94 | 1 |
| IGF1R | 94 | 1 |
| JAK1(JH2domain-pseudokinase) | 94 | 1 |
| MAP3K1 | 94 | 1 |
| MAP4K3 | 94 | 1 |
| MET(Y1235D) | 94 | 1 |
| MLK3 | 94 | 1 |
| PAK1 | 94 | 1 |
| PIK3CA(E545A) | 94 | 1 |

| | | |
|---|---|---|
| PLK2 | 94 | 1 |
| PRKCD | 94 | 1 |
| RAF1 | 94 | 1 |
| RIOK1 | 94 | 1 |
| SBK1 | 94 | 1 |
| SRPK3 | 94 | 1 |
| STK35 | 94 | 1 |
| TLK1 | 94 | 1 |
| VPS34 | 94 | 1 |
| ABL1(T315I)-phosphorylated | 95 | 1 |
| ALK(L1196M) | 95 | 1 |
| CAMK1D | 95 | 1 |
| CDK11 | 95 | 1 |
| CSNK1G3 | 95 | 1 |
| ERK3 | 95 | 1 |
| FLT3(R834Q) | 95 | 1 |
| MLK1 | 95 | 1 |
| MST3 | 95 | 1 |
| MYO3A | 95 | 1 |
| PKAC-alpha | 95 | 1 |
| PLK3 | 95 | 1 |
| PRKG2 | 95 | 1 |
| PRKR | 95 | 1 |
| S6K1 | 95 | 1 |
| TAOK3 | 95 | 1 |
| TSSK3 | 95 | 1 |
| TTK | 95 | 1 |
| TYK2(JH2domain-pseudokinase) | 95 | 1 |
| WNK2 | 95 | 1 |
| ABL2 | 96 | 1 |
| AKT2 | 96 | 1 |
| EPHB1 | 96 | 1 |
| FGR | 96 | 1 |
| GRK2 | 96 | 1 |
| ITK | 96 | 1 |

| | | |
|---|---|---|
| JAK3(JH1domain-catalytic) | 96 | 1 |
| KIT(V559D,V654A) | 96 | 1 |
| LYN | 96 | 1 |
| MLCK | 96 | 1 |
| MYO3B | 96 | 1 |
| NEK11 | 96 | 1 |
| PIK3CA(I800L) | 96 | 1 |
| PIK3CD | 96 | 1 |
| ABL1(T315I)-nonphosphorylated | 97 | 1 |
| BRAF | 97 | 1 |
| CAMK1B | 97 | 1 |
| CDC2L1 | 97 | 1 |
| CDKL3 | 97 | 1 |
| CLK2 | 97 | 1 |
| CSNK1G1 | 97 | 1 |
| DLK | 97 | 1 |
| MAP3K4 | 97 | 1 |
| MAP4K2 | 97 | 1 |
| MAPKAPK5 | 97 | 1 |
| PIK4CB | 97 | 1 |
| PLK1 | 97 | 1 |
| SGK2 | 97 | 1 |
| SYK | 97 | 1 |
| TXK | 97 | 1 |
| ULK3 | 97 | 1 |
| WEE2 | 97 | 1 |
| ALK | 98 | 1 |
| CAMK2A | 98 | 1 |
| CDK7 | 98 | 1 |
| DYRK1A | 98 | 1 |
| EPHB3 | 98 | 1 |
| ERK5 | 98 | 1 |
| FAK | 98 | 1 |
| FLT3(K663Q) | 98 | 1 |
| GCN2(Kin.Dom.2,S808G) | 98 | 1 |

| | | |
|---|---|---|
| HIPK3 | 98 | 1 |
| IRAK1 | 98 | 1 |
| LKB1 | 98 | 1 |
| MARK2 | 98 | 1 |
| MET | 98 | 1 |
| MRCKB | 98 | 1 |
| NEK3 | 98 | 1 |
| PCTK1 | 98 | 1 |
| PCTK2 | 98 | 1 |
| PFTK1 | 98 | 1 |
| PIM2 | 98 | 1 |
| PKN2 | 98 | 1 |
| SGK | 98 | 1 |
| TSSK1B | 98 | 1 |
| ABL1(M351T)-phosphorylated | 99 | 1 |
| ACVR1 | 99 | 1 |
| ANKK1 | 99 | 1 |
| CAMKK2 | 99 | 1 |
| DRAK2 | 99 | 1 |
| FLT3(N841I) | 99 | 1 |
| HIPK1 | 99 | 1 |
| LRRK2(G2019S) | 99 | 1 |
| MAP3K2 | 99 | 1 |
| MEK4 | 99 | 1 |
| MKNK2 | 99 | 1 |
| MYLK4 | 99 | 1 |
| NEK1 | 99 | 1 |
| NIM1 | 99 | 1 |
| PIKFYVE | 99 | 1 |
| PRKD1 | 99 | 1 |
| PRKD3 | 99 | 1 |
| RSK1(Kin.Dom.1-N-terminal) | 99 | 1 |
| RSK3(Kin.Dom.2-C-terminal) | 99 | 1 |
| RSK4(Kin.Dom.2-C-terminal) | 99 | 1 |
| STK36 | 99 | 1 |
| WEE1 | 99 | 1 |

| | | |
|---|---|---|
| YANK3 | 99 | 1 |
| AAK1 | 100 | 1 |
| ACVR2B | 100 | 1 |
| ACVRL1 | 100 | 1 |
| ADCK4 | 100 | 1 |
| AKT1 | 100 | 1 |
| AKT3 | 100 | 1 |
| ALK(C1156Y) | 100 | 1 |
| AMPK-alpha1 | 100 | 1 |
| AURKA | 100 | 1 |
| BIKE | 100 | 1 |
| BMPR1B | 100 | 1 |
| BMPR2 | 100 | 1 |
| BRSK1 | 100 | 1 |
| BUB1 | 100 | 1 |
| CAMK1 | 100 | 1 |
| CAMK1G | 100 | 1 |
| CAMK2D | 100 | 1 |
| CAMK2G | 100 | 1 |
| CAMK4 | 100 | 1 |
| CAMKK1 | 100 | 1 |
| CDC2L2 | 100 | 1 |
| CDK3 | 100 | 1 |
| CDK4-cyclinD3 | 100 | 1 |
| CDK5 | 100 | 1 |
| CDK9 | 100 | 1 |
| CHEK2 | 100 | 1 |
| CIT | 100 | 1 |
| CSK | 100 | 1 |
| CSNK1A1 | 100 | 1 |
| CSNK1A1L | 100 | 1 |
| CSNK2A2 | 100 | 1 |
| DCAMKL2 | 100 | 1 |
| DCAMKL3 | 100 | 1 |
| DMPK | 100 | 1 |
| EIF2AK1 | 100 | 1 |

| | | |
|---|---|---|
| EPHA5 | 100 | 1 |
| EPHB2 | 100 | 1 |
| EPHB4 | 100 | 1 |
| ERK2 | 100 | 1 |
| ERK4 | 100 | 1 |
| FLT3(D835V) | 100 | 1 |
| FLT3(ITD) | 100 | 1 |
| FLT3(ITD,F691L) | 100 | 1 |
| FLT3-autoinhibited | 100 | 1 |
| FYN | 100 | 1 |
| GSK3B | 100 | 1 |
| HCK | 100 | 1 |
| HIPK2 | 100 | 1 |
| HPK1 | 100 | 1 |
| IKK-alpha | 100 | 1 |
| IKK-beta | 100 | 1 |
| IKK-epsilon | 100 | 1 |
| INSRR | 100 | 1 |
| IRAK3 | 100 | 1 |
| IRAK4 | 100 | 1 |
| KIT-autoinhibited | 100 | 1 |
| LATS1 | 100 | 1 |
| LRRK2 | 100 | 1 |
| MAK | 100 | 1 |
| MAP4K4 | 100 | 1 |
| MAP4K5 | 100 | 1 |
| MARK3 | 100 | 1 |
| MEK6 | 100 | 1 |
| MKK7 | 100 | 1 |
| MRCKA | 100 | 1 |
| MST1 | 100 | 1 |
| MST1R | 100 | 1 |
| MTOR | 100 | 1 |
| MUSK | 100 | 1 |
| MYLK | 100 | 1 |
| NEK10 | 100 | 1 |

| | | |
|---|---|---|
| NEK6 | 100 | 1 |
| NEK9 | 100 | 1 |
| p38-delta | 100 | 1 |
| p38-gamma | 100 | 1 |
| PAK3 | 100 | 1 |
| PAK4 | 100 | 1 |
| PAK6 | 100 | 1 |
| PCTK3 | 100 | 1 |
| PDGFRA | 100 | 1 |
| PHKG1 | 100 | 1 |
| PIK3CA(C420R) | 100 | 1 |
| PIK3CA(M1043I) | 100 | 1 |
| PIM1 | 100 | 1 |
| PIM3 | 100 | 1 |
| PIP5K1A | 100 | 1 |
| PIP5K1C | 100 | 1 |
| PIP5K2B | 100 | 1 |
| PIP5K2C | 100 | 1 |
| PKAC-beta | 100 | 1 |
| PKN1 | 100 | 1 |
| PLK4 | 100 | 1 |
| PRKCE | 100 | 1 |
| PRKCQ | 100 | 1 |
| PRKG1 | 100 | 1 |
| PRKX | 100 | 1 |
| RIOK3 | 100 | 1 |
| RIPK2 | 100 | 1 |
| ROCK1 | 100 | 1 |
| RPS6KA4(Kin.Dom.1-N-terminal) | 100 | 1 |
| RPS6KA4(Kin.Dom.2-C-terminal) | 100 | 1 |
| RSK2(Kin.Dom.1-N-terminal) | 100 | 1 |
| RSK2(Kin.Dom.2-C-terminal) | 100 | 1 |
| RSK3(Kin.Dom.1-N-terminal) | 100 | 1 |
| RSK4(Kin.Dom.1-N-terminal) | 100 | 1 |

| | | |
|---|---|---|
| SgK110 | 100 | 1 |
| SGK3 | 100 | 1 |
| SIK | 100 | 1 |
| SNARK | 100 | 1 |
| STK16 | 100 | 1 |
| TBK1 | 100 | 1 |
| TESK1 | 100 | 1 |
| TGFBR2 | 100 | 1 |
| TLK2 | 100 | 1 |
| TRKA | 100 | 1 |
| TRKB | 100 | 1 |
| TRKC | 100 | 1 |
| TRPM6 | 100 | 1 |
| TYK2(JH1domain-catalytic) | 100 | 1 |
| ULK2 | 100 | 1 |
| WNK1 | 100 | 1 |
| WNK4 | 100 | 1 |
| YANK1 | 100 | 1 |
| YSK4 | 100 | 1 |
| ZAK | 100 | 1 |
| ZAP70 | 100 | 1 |

**Table S6.** Biochemical activities against a panel of kinase targets

| YNW-1 | |
|---|---|
| ERBB2 (HER2) | 126 |
| ERBB4 (HER4) | 73.6 |
| FLT4 (VEGFR3) | 108 |
| MAPK14 (p38 alpha) Direct | 732 |
| TEK (Tie2) | 250 |
| EPHA6 | >10000 |

[a]IC50 (nM) against EGFR L858R, BTK, JAK3, ITK and BLK were obtained with Z'-LYTE

biochemical assay. [b] Kd (nM) of EPHA6 was obtained from KdELECT assays (DiscoverX).

**Chemistry**

Unless otherwise stated, all reagents and solvents were purchased from commercial suppliers and used without further purification. $^1$H NMR spectra were recorded on a Bruker A400 or A500 spectrometer operating at 400 or 500 MHz. Chemical shifts (δ) are reported in parts per million (ppm, d) relative to tetramethyl silane (TMS) as an internal standard. Coupling constants (J) are given in hertz (Hz). Signal multiplicities are designated as follows: s (singlet), br (broad singlet), d (doublet), t (triplet), q (quartet), and m (multiplet). All compounds used in biological assays were confirmed to be >95% purities by analytical reverse-phase HPLC.

**Synthesis of ZNL-1**

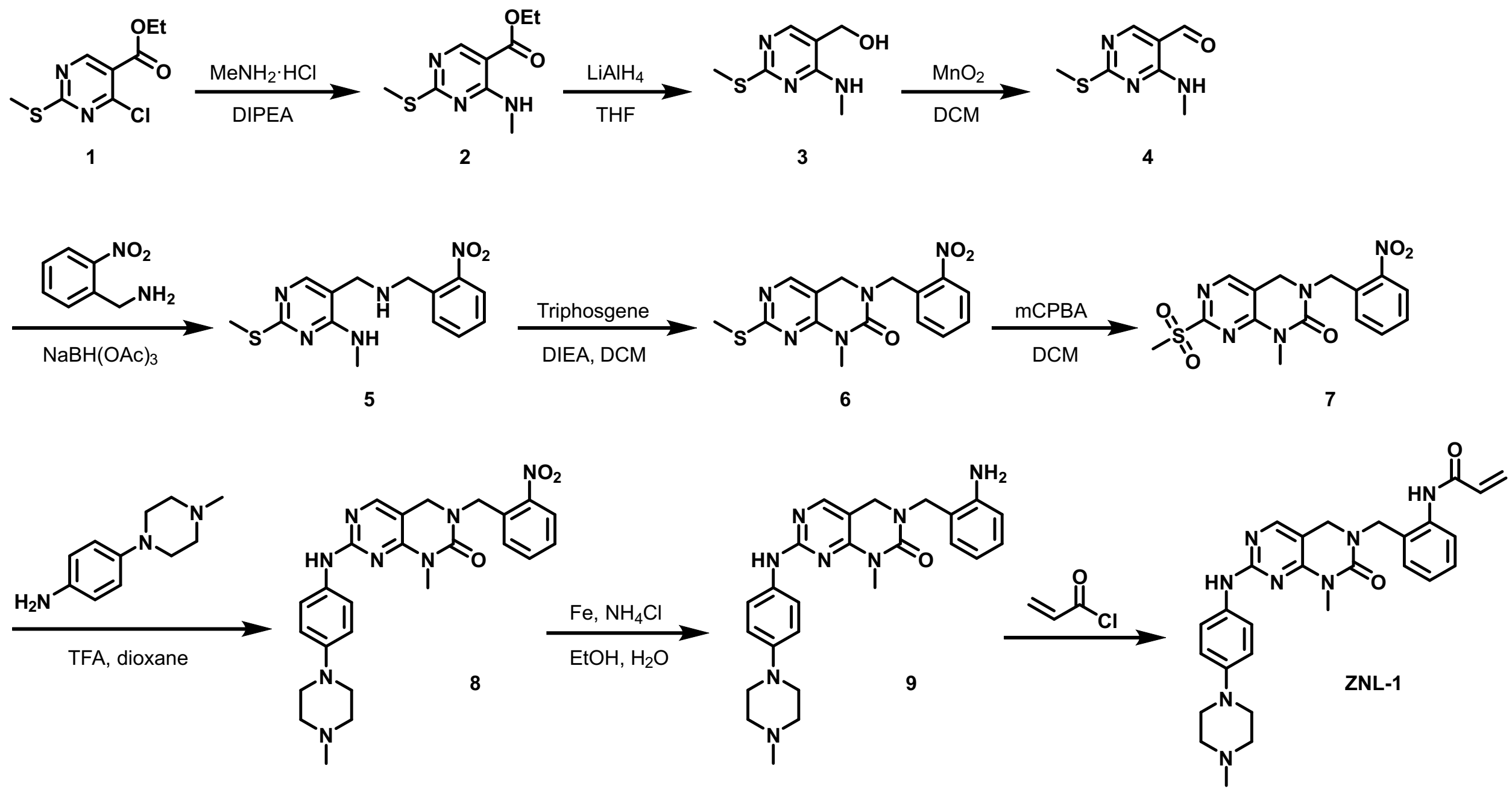


**Compound 2**:

To a solution of ethyl 4-chloro-2-methylsulfanyl-pyrimidine-5-carboxylate **1** (10 g, 42.98 mmol, 1 eq) in THF (200 mL) was added TEA (10.00 g, 98.85 mmol, 13.76 mL, 2.3 eq) and methenamine hydrochloride (6.67 g, 98.85 mmol, 2.3 eq) at 0°C.The mixture was stirred at 0°C for 1 hr and at 25°C for 12 hr. LCMS showed complete consumption of the reactant and formation of the desired product mass. The reaction mixture was concentrated under reduced pressure. The residue was diluted with $H_2O$ 100 mL and extracted with EtOAc

(100 mL × 3). The combined organic layers were washed with brine (100 mL × 3), dried over $Na_2SO_4$, filtered and concentrated under reduced pressure to give **2** (9.4 g) as a white solid.

**Compound 3**:

The suspension of LAH (2.35 g, 62.04 mmol, 1.5 eq) in THF (40 mL) was cooled at 0°C and to this the solution of **2** (9.4 g, 41.36 mmol, 1 eq) in THF (20 mL) was added dropwise under argon and allowed to stir at 0 °C for 30 min. The reaction was quenched by addition of saturated $MgSO_4$ solution (20 mL). The mixture and filtered and the filtrate concentrated under reduced pressure to give **3** (5.5 g, crude) as a yellow solid.

**Compound 4:**

To a solution of **3** (3.5 g, 18.89 mmol, 1 eq) in DCM (50 mL) was added $MnO_2$ (9.86 g, 113.36 mmol, 6 eq). The mixture was stirred at 40°C for 12 hr. The reaction mixture was filtered and the filtrate was concentrated under reduced pressure to give a residue, the residue was purified by flash silica gel chromatography (ISCO®; 20 g SepaFlash® Silica Flash Column, Eluent of 5~50% Ethyl acetate/Petroleum ether gradient @ 80mL/min) to give **4** (3 g, 16.37 mmol, 87% yield) as a yellow solid.

**Compound 5:**

To a mixture of (2-nitrophenyl)methanamine hydrochloride (2 g, 10.60 mmol, 1 eq) in DCE (30 mL) was added TEA to adjust pH~=8, **4** (2.91 g, 15.91 mmol, 1.5 eq) and AcOH was added to adjust pH~=5 and the mixture was stirred at 25°C for 2 hr. $NaBH(OAc)_3$ (6.74 g, 31.81 mmol, 3 eq) was added to the reaction. The mixture was stirred at 25°C for 12 hr. The reaction mixture was partitioned between $H_2O$ 30 mL and DCM 30 mL. The water phase was separated, extracted with DCM (30 mL × 3), dried over $Na_2SO_4$, filtered and concentrated under reduced pressure to give a residue. The residue was purified by flash silica gel chromatography (ISCO®; 20 g SepaFlash® Silica Flash Column, Eluent of 0~65% Ethyl acetate/Petroleum ether gradient @ 80mL/min) to give **5** (2.65 g, 8.30 mmol, 78.25% yield) as a yellow oil.

**Compound 6:**

To a solution of **5** (2.65 g, 8.30 mmol, 1 eq) in DCM (30 mL) was added DIEA (3.22 g, 24.89 mmol, 4.34 mL, 3 eq) and bis(trichloromethyl) carbonate (984.88 mg, 3.32 mmol, 0.4 eq) in DCM (10 mL) at 0°C. The mixture was stirred at 25°C for 2 hr. LCMS showed complete consumption of the reactant and formation of the desired product mass. The reaction mixture was partitioned between $NaHCO_3$ 50 mL and DCM 50 mL. The water phase was separated, extracted with DCM (50 mL × 3), dried over $Na_2SO_4$, filtered and concentrated under reduced pressure to give a residue. The residue was purified by flash silica gel chromatography (ISCO®; 20 g SepaFlash® Silica Flash Column, Eluent of 0~100% Ethyl acetate/Petroleum ether gradient @80 mL/min) to give **6** (700 mg, 2.03 mmol, 24.43% yield) as a yellow solid.

**Compound 7:**

A solution of m-CPBA (1.53 g, 7.53 mmol, 85% purity, 4 eq) in DCM (10 mL) was added to **6** (650 mg, 1.88 mmol, 1 eq) in DCM (10 mL) at 0°C. The mixture was stirred at 25°C for 12 hr. LCMS showed complete consumption of the reactant and formation of the desired product mass. The residue was diluted with $Na_2SO_3$ (10%) 14 mL and extracted with DCM (10 mL × 3). The combined organic layers were washed with $NaHCO_3$ (10 mL × 3), dried over $Na_2SO_4$, filtered and concentrated under reduced pressure to give a residue. The crude material was suspended in ethanol and stirred at room temperature for 30 min. The solid was filtered, washed with ethanol and dried. The residue was purified by flash silica gel chromatography (ISCO®; 12 g SepaFlash® Silica Flash Column, Eluent of 0~100% Ethyl acetate/Petroleum ether gradient @ 80 mL/min) to give **7** (450 mg, 1.19 mmol, 63.36% yield) as yellow gum.

**Compound 8:**

To a solution of **7** (160 mg, 423.98 umol, 1 eq) in dioxane (5 mL) was added TFA (72.52 mg, 635.97 umol, 47.09 uL, 1.5 eq) and 4-(4-methylpiperazin-1-yl) aniline (121.64 mg, 635.97 umol, 1.5 eq). The mixture was stirred at 120°C for 12 hr. The reaction mixture was concentrated under reduced pressure. The residue was diluted with $NaHCO_3$ 10 mL and extracted with EtOAc (10 mL × 3). The combined organic layers were washed with brine (10 mL × 3), dried over $Na_2SO_4$, filtered and concentrated under reduced pressure to give a residue. The residue was purified by prep-TLC (SiO2, DCM: MeOH = 10:1) to give **8** (180 mg, 305.81 umol, 72.13% yield, 83% purity) as a yellow solid.

**Compound 9:**

To a solution of **8** (180 mg, 368.44 umol, 1 eq) in EtOH (6 mL) and $H_2O$ (2 mL) was added $NH_4Cl$ (39.42 mg, 736.89 umol, 2 eq) and stirred at 25°C for 5 min. Then the mixture was heated to 90°C. Fe (82.30 mg, 1.47 mmol, 4 eq) was added to the mixture. The mixture was stirred at 90°C for 2 hr. The reaction mixture was filtered and the filtrate dried in vacuum to give a residue. The residue was purified by prep-TLC ($SiO_2$, 10% methol in DCM) to give **9** (110 mg, 239.9 umol, 65% yield) as a yellow solid.

**ZNL-1**:

To a solution of **9** (50 mg, 109.04 umol, 1 eq) and DIPEA (42.3 mg, 327.11 umol, 54.1 uL, 3 eq) in DMF (1 mL) was added and prop-2-enoyl chloride (9.87 mg, 109.04 umol, 8.9 uL, 1 eq) at 0°C. The mixture was stirred at 0°C for 90 min. The reaction mixture was concentrated under reduced pressure to give a residue. The residue was purified by prep-HPLC (FA condition, Phenomenex Luna C18 75*30mm*3um; [water (FA)-ACN]; B%:1%-30%, 8min) to give **ZNL-1** (11 mg, 18.54 umol, 17.01% yield, 94.166% purity, FA) as a white solid. MS (ESI): m/z = 513.2 $[M+H]^+$

$^1$HNMR (400 MHz, DMSO-*d*$_6$) *δ* ppm 9.93 (s, 1 H), 9.24 (s, 1 H), 8.18 ( s, 1 H), 8.01 (s, 1 H), 7.77 (d, J=8.0 Hz, 1 H), 7.56 (d, J=8.0 Hz, 2 H), 7.35-7.22 (m, 2 H), 7.21-7.17 (m, 1 H), 6.88 (d, J=8.0 Hz, 2 H), 6.53-6.46 (m, 1 H), 6.30-6.25 (m, 1 H), 5.82-5.79 (m, 1 H), 4.61 (s, 2 H), 4.26 (s, 2 H), 3.31 (s, 3 H), 3.07-3.04 (m, 4 H), 2.51-2.48 (m, 4 H), 2.25 (s, 3 H).

**Synthesis of ZNL-2 (*S*)/(*R*)**

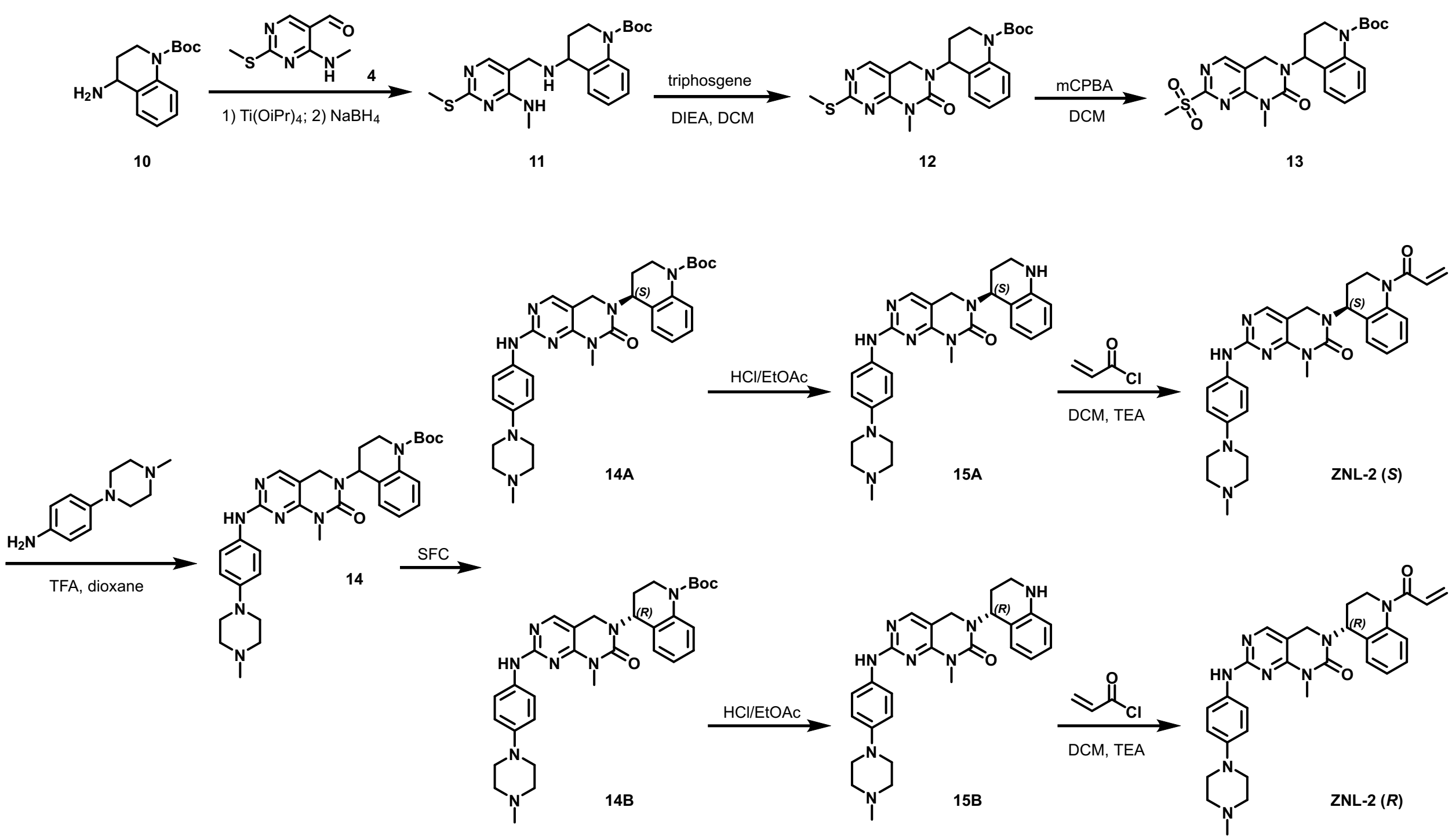


Compound **11**:

To a solution of **4** (3 g, 16.37 mmol, 1 eq) in MeOH (100 mL) was added **10** (4.07 g, 16.37 mmol, 1 eq) and Ti(O$^i$Pr)$_4$ (13.96 g, 49.12 mmol, 14.50 mL, 3 eq). The mixture was stirred at 30°C for 12 hr. Then $NaBH_4$ (3.72 g, 98.24 mmol, 6 eq) was added to the mixture at 0°C. The mixture was stirred at 25°C for 3 hr. The reaction mixture was concentrated under reduced pressure to give a residue. The reaction mixture was quenched by addition $NH_4Cl$ (100 mL) at 0°C. The reaction mixture was extracted with acetate ethyl (100 mL × 3). The combined organic layers were washed with brine (30 mL × 2), dried over $Na_2SO_4$, filtered and concentrated under reduced pressure to give a residue. The residue was purified by flash silica gel chromatography (ISCO®; 40 g SepaFlash® Silica Flash Column, Eluent of 0~50% Ethyl acetate/Petroleum ether gradient @ 120 mL/min) to **11** (6 g) as a light-yellow oil.

Compound **12**:

To a solution **11** (6 g, 14.44 mmol, 1 eq) and DIEA (9.33 g, 72.19 mmol, 12.57 mL, 5 eq) in DCM (100 mL) was added triphosgene (4.28 g, 14.44 mmol, 1 eq) at 0°C. The mixture was stirred at 25°C for 12 hr. The reaction mixture was partitioned between $NaHCO_3$ (100 mL) and dDCM (100 mL × 2). The organic phase was separated, washed with brine (100 mL × 1), dried over $Na_2SO_4$, filtered and concentrated under reduced pressure to give a residue. The residue was purified by flash silica gel chromatography (ISCO®; 40 g SepaFlash® Silica Flash Column, Eluent of 0~50% Ethyl acetate/Petroleum ether gradient @ 120 mL/min) to **12** (5.8 g, 11.71 mmol, 81.13% yield, 89.181% purity) as a light-yellow oil.

Compound **13**:

To a solution of **12** (3 g, 6.79 mmol, 1 eq) in DCM (50 mL) was added m-CPBA (2.21 g, 10.87 mmol, 85% purity, 1.6 eq) at 0°C. The mixture was stirred at 25°C for 12 hr. The residue was diluted with $Na_2SO_3$ (50 mL) and extracted with dichloromethane (50 mL × 3). The combined organic layers were washed with $NaHCO_3$ (50 mL × 3), dried over $Na_2SO_4$, filtered and concentrated under reduced pressure to **13** (3.2 g, crude) as a white solid.

Compound **14, 14A, 14B**:

To a solution of **13** (2 g, 4.22 mmol, 1 eq) in dioxane (20 mL) was added TFA (722.34 mg, 6.34 mmol, 469.05 uL, 1.5 eq) and 4-(4-methylpiperazin-1-yl) aniline (969.40 mg, 5.07 mmol, 1.2 eq). The mixture was stirred at 120°C for 12 hr. The residue was purified by prep-HPLC (FA condition, column: Phenomenex luna C18 (250×70mm, 15 um); mobile phase: [water (FA)-ACN]; B%: 30%-60%, 20min) to give product **14**. Then **14** was separated by SFC column: DAICEL CHIRALPAK AD (250mm×50mm,10um); mobile phase: A: $CO_2$ B: EtOH (0.1%IPAm, v/v); B%: 60%-60%, 7min to give **14A** (Rt = 1.709 min, 500 mg, 855.12 umol, 20.25% yield) and **14B** (Rt =1.993 min, 530 mg, 906.43 umol, 21.46% yield) as a brown solid.

Compound **15A, 15B**:

To a solution of **14A** or **14B** (350 mg, 598.6 umol, 1 eq) in HCl/EtOAc (4 M, 3.5 mL, 23.3 eq) was stirred at 25°C for 1 hr. The reaction mixture was concentrated under reduced pressure to give product **15A** or **15B** as yellow solid used into the next step without further purification.

**ZNL-2 (*S*):**

To a solution of **15A** (125 mg, 239.9 umol, 1 eq, HCl) and DIEA (93.0 mg, 719.7 umol, 125.3 uL, 3 eq) in DMF (2 mL) was added a DCM solution of prop-2-enoyl chloride (26.1 mg, 287.9 umol, 23.5 uL, 1.2 eq) at 0°C. The reaction was stirred at 0°C for 1 hr. The reaction mixture was filtered. The residue was purified by prep-HPLC (FA condition; column:Phenomenex luna C18 100×40mm×3 um; mobile phase: [water(FA)-ACN]; B%: 1%-40%,8min), and then the residue was purified by prep-HPLC (neutral condition; column: Waters Xbridge Prep OBD C18 150×40mm×10um; mobile phase: [water ($NH_4HCO_3$)-ACN]; B%: 20%-50%, 8min) to yield **ZNL-2 (*S*)** (100% purity, 69 mg) as a white solid. MS (ESI): m/z = 539.2 $[M+H]^+$

VT (Variable Temperature) -$^1$H NMR (400 MHz, DMSO-*d*$_6$) $\delta$ = 8.96 - 8.80 (m, 1H), 7.99 - 7.84 (m, 1H), 7.59 - 7.43 (m, 2H), 7.30 - 7.15 (m, 3H), 6.86 (d, J = 9.0 Hz, 2H), 6.72 - 6.51 (m, 1H), 6.29 - 6.12 (m, 1H), 5.78 - 5.68 (m, 1H), 5.68 - 5.50 (m, 1H), 4.25 - 4.05 (m, 2H), 3.96 - 3.82 (m, 1H), 3.80 - 3.69 (m, 1H), 3.62 - 3.49 (m, 1H), 3.34 (s, 3H), 3.10 - 3.04 (m, 4H), 2.46 (br d, J = 4.9 Hz, 4H), 2.28 - 2.17 (m, 4H), 2.15 - 1.96 (m, 1H)

**ZNL-2 (*S*)** was also made starting with **(*S*)-10**, and the absolutely configuration was confirmed by SFC comparing.

**ZNL-2 (*R*):**

To a solution of **15B** (300 mg, 575.75 umol, 1 eq, HCl) and TEA (174.78 mg, 1.73 mmol, 240.41 uL, 3 eq) in DCM (5 mL) was added a DCM solution of prop-2-enoyl chloride (52.11 mg, 575.75 umol, 46.95 uL, 1 eq). The mixture was stirred at 0°C for 1 hr. The reaction mixture was concentrated under reduced pressure to give a residue. The residue was purified by prep-HPLC (FA condition, column: Phenomenex Luna C18 75×30mm×3um ;mobile phase: [water(FA)-ACN]; B%:1%-35%,8min) and (NH4HCO3 condition, column: Phenomenex C18 75×30mm×3um;mobile phase: [water( NH4HCO3)-ACN]; B%: 20%-55%,8min) to give **ZNL-2 (*R*)** (76.10 mg, 139.89 umol, 24.30% yield, 99.016% purity) as a yellow solid. MS (ESI): m/z = 539.2 $[M+H]^+$

**ZNL-2 (*R*)** was also made starting with **(*R*)-10**, and the absolutely configuration was confirmed by SFC comparing.

**Synthesis of ZNL-3**

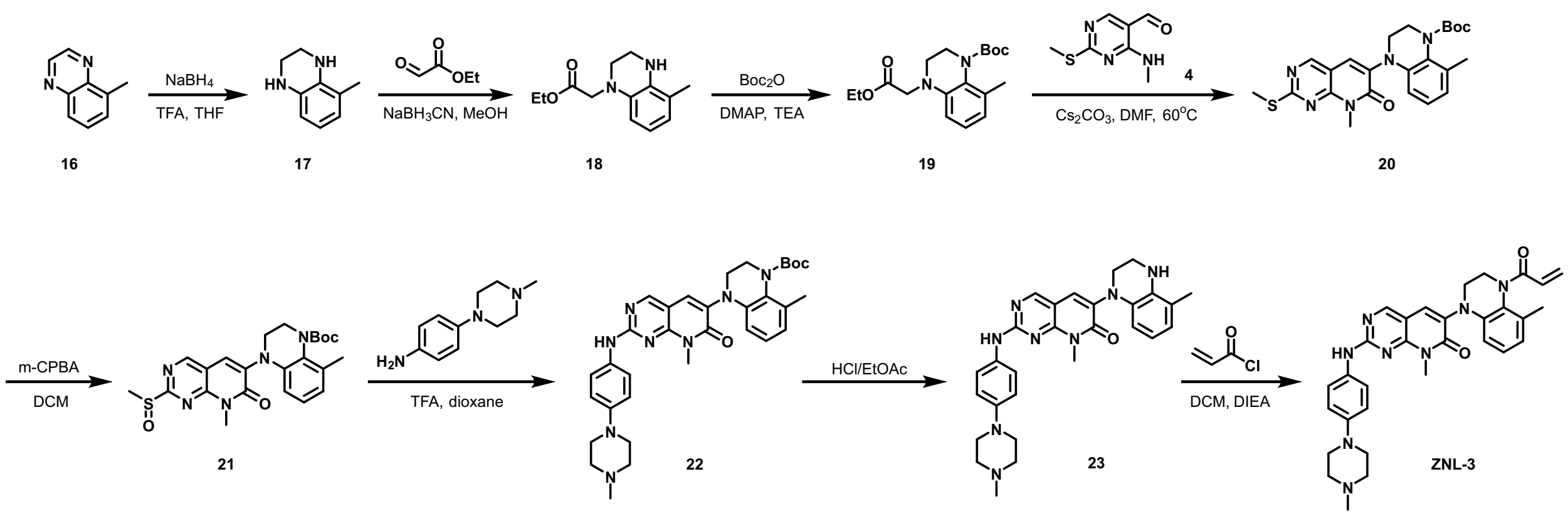


**Compound 17:**

To a solution of 5-methylquinoxaline **16** (5.0 g, 34.7 mmol, 1 *eq*) in THF (50 mL) was added $NaBH_4$ (4.6 g, 121.4 mmol, 3.5 *eq*). The mixture was stirred at 0°C for 0.5 hr. TFA (7.1 g, 62.4 mmol, 4.6 mL, 1.8 *eq*) was added to the mixture at 0°C. The mixture was stirred at 0°C for 0.5 hr. Then the mixture was stirred at 20°C for 12 hr. The reaction mixture was added water (100 mL) at 0°C. The mixture was extracted with ethyl acetate (20 mL × 3). The combined organic layers were washed with brine (15 mL × 2), dried over $Na_2SO_4$, filtered and concentrated under reduced pressure to give a residue. The residue was purified by column chromatography ($SiO_2$, Petroleum ether/Ethyl acetate=5/1 to 3/1) to give 5-methyl-1,2,3,4-tetrahydroquinoxaline **17** (4.7 g, 31.7 mmol, 91.4% yield) as red oil.

**Compound 18:**

To a solution of **17** (4.3 g, 29.0 mmol, 1 *eq*) and ethyl 2-oxoacetate (6.5 g, 31.9 mmol, 50% purity, 1.1 *eq*) in MeOH (100 mL) was adjust pH to 4-5 by AcOH, the mixture was stirred at 25°C for 3 hr, then $NaBH_3CN$ (5.5 g, 87.0 mmol, 3 *eq*) was added, the mixture was stirred at 25°C for 12 hr. The mixture was concentrated to get a residue, the residue was quenched by Sat. $NaHCO_3$ (20 mL) and then extracted by ethyl acetate (30 mL × 3), the combined organic layers was dried by $Na_2SO_4$, filtered and the filtrate was concentrated

to get a residue. The residue was purified by column chromatography ($SiO_2$, Petroleum ether /Ethyl acetate=10/1 to 3/1) to give ethyl 2-(5-methyl-3,4-dihydro-2H-quinoxalin-1-yl) acetate **18** (3 g, 12.8 mmol, 44.1% yield) as brown oil.

**Compound 19:**

To a solution of **18** (2.5 g, 10.7 mmol, 1 *eq*) in $Boc_2O$ (15 mL) was added DMAP (130.4 mg, 1.1 mmol, 0.1 *eq*) and TEA (5.4 g, 53.4 mmol, 7.4 mL, 5 *eq*). The mixture was stirred at 80°C for 48 hr. The mixture was concentrated to get a residue. The residue was purified by column chromatography ($SiO_2$, Petroleum ether / Ethyl acetate=10/1 to 5/1) to give **19** (2.45 g, 7.3 mmol, 68.7% yield) as red oil.

**Compound 20:**

To a solution of **19** (2.4 g, 7.3 mmol, 1 *eq*) in DMF (20 mL) was added $Cs_2CO_3$ (7.2 g, 22.0 mmol, 3 *eq*) and 4-(methylamino)-2-methylsulfanyl-pyrimidine-5-carbaldehyde **4** (1.5 g, 8.1 mmol, 1.1 *eq*). The mixture was stirred at 80°C for 12 hr. The mixture was poured into water (100 mL) and then extracted by ethyl acetate (30 mL × 3), the organic layers was washed by brine (20 mL × 2), dried by $Na_2SO_4$, then filtered and the filtrate was concentrated to get a residue. The residue was purified by flash silica gel chromatography (25 g Silica Flash Column, Eluent of 0~15% Ethyl acetate/Petroleum ether gradient @ 120 mL /min) to give **20** (2.1 g, 4.6 mmol, 63.2% yield) as a yellow solid.

**Compound 21:**

To a solution of **20** (2.1 g, 4.63 mmol, 1 *eq*) in DCM (50 mL) was added m-CPBA (1.7 g, 8.3 mmol, 85% purity, 1.8 eq). The mixture was stirred at 25°C for 12 hr. The reaction mixture was partitioned between $Na_2SO_3$ (30 mL) and dichloromethane (30 mL × 3). The organic phase was separated, washed with $NaHCO_3$ (20 mL × 3), dried over $Na_2SO_4$, filtered and concentrated under reduced pressure to give a residue. The residue was purified by column chromatography ($SiO_2$, Petroleum ether/Ethyl acetate=10/1 to 1/1) to give compound **21** (1 g, 2.1 mmol, 46.0% yield) as a red solid.

**Compound 22:**

To a solution of **21** (400 mg, 857.8 umol, 1 *eq*) in dioxane (4 mL) was added TFA (116.6 mg, 1022.2 umol, 75.9 uL, 1.2 *eq*) and 4-(4-methylpiperazin-1-yl) aniline (163.7 mg, 851.9 umol, 1 *eq*). The mixture was stirred at 80°C for 12 hr. The mixture was concentrated to get a residue. The residue was purified by prep-HPLC (FA condition; column: Phenomenex luna C18 100 × 40mm × 3 um; mobile phase: [water (TFA)-ACN]; B%: 25%-70%,8min) to give **22** (300 mg, 502.8 umol, 59.0% yield) as a red solid.

**Compound 23:**

To a solution of **22** (300 mg, 502.8 umol, 1 *eq*) in EtOAc (5 mL) was added HCl/EtOAc (10 mL, 4M). The mixture was stirred at 25°C for 1 hr. The mixture was concentrated to give **23** (250 mg, crude) as a yellow solid.

**ZNL-3:**

To a solution of **23** (100 mg, 187.6 umol, 1 eq, HCl) in DCM (1 mL) was added DIEA (97.0 mg, 750.4 umol, 130.7 uL, 4 eq) and prop-2-enoyl chloride (17.0 mg, 187.6 umol, 15.3 uL, 1 eq). The mixture was stirred at 0°C for 1 hr. The mixture was concentrated to get a residue. The residue was purified by prep-HPLC (FA condition; column: Phenomenex Luna C18 200 × 40mm × 10um; mobile phase: [water (FA)-ACN]; B%:5%-40%, 8min) to give compound **ZNL-3** (28.1 mg, 51.0 umol, 27.2% yield, 100% purity) as a white solid. MS (ESI): m/z = 551.3 $[M+H]^+$

$^1H$ NMR (400 MHz, DMSO-$d_6$) $\delta$ = 9.89 (br d, $J$ = 1.0 Hz, 1H), 8.71 (s, 1H), 7.90 (s, 1H), 7.66 (br d, $J$ = 8.1 Hz, 2H), 6.99 - 6.86 (m, 3H), 6.64 (d, $J$ = 8.0 Hz, 1H), 6.35 (d, $J$ = 8.0 Hz, 1H), 6.31 - 6.23 (m, 2H), 5.73 (m, 1H), 4.88 (m, 1H), 3.70 (m, 1H), 3.63 - 3.55 (m, 3H), 3.41 (m, 1H), 3.13 - 3.06 (m, 4H), 3.01 (m, 1H), 2.48 - 2.43 (m, 4H), 2.22 (s, 3H), 2.15 - 2.01 (m, 3H)

**Synthesis of ZNL-3r**

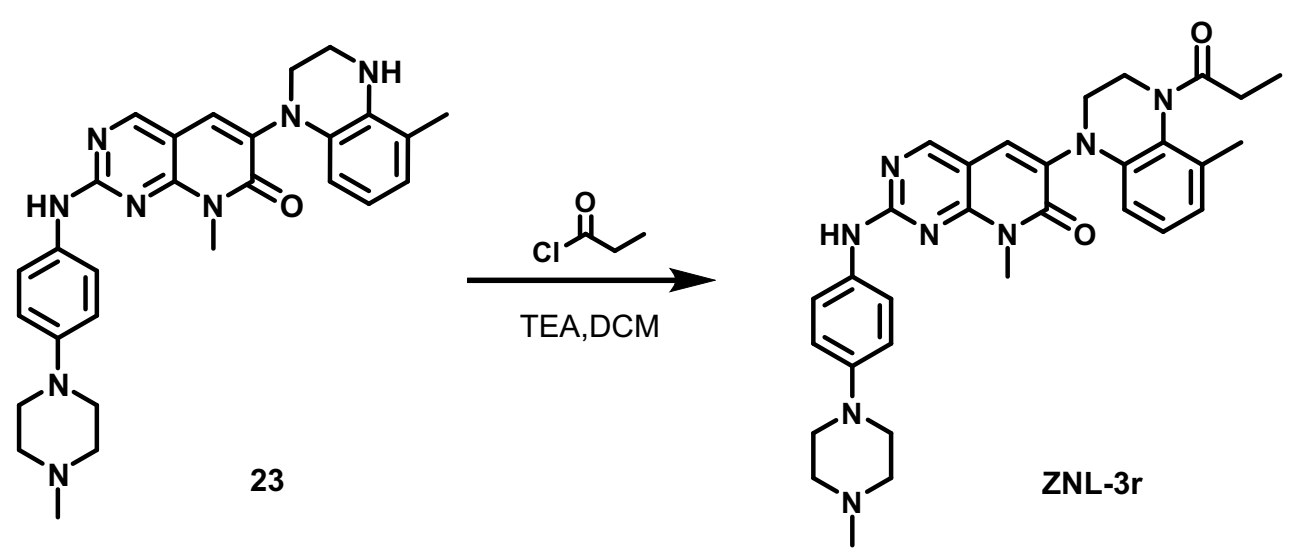


To a solution of **23** (60 mg, 112.6 umol, 1 *eq*, HCl) in DCM (3 mL) was added TEA (34.2 mg, 337.7 umol, 47.0 uL, 3 *eq*) and propanoyl chloride (20.8 mg, 225.1 umol, 20.8 uL, 2 *eq*) at 0°C. The mixture was stirred at 25°C for 12 hr. The reaction mixture was concentrated under reduced pressure to remove **solvent**. The residue was purified by prep-HPLC (**FA condition** column: Phenomenex C 18 75 × 30 mm × 3um; mobile phase: [water (FA)-ACN]; B%: 10%-45%, 8 min) to compound **ZNL-3r** (27.8 mg, 50.1 umol, 44.5% yield, 99.8% purity) as a yellow solid. MS (ESI): m/z = 553.5 $[M+H]^+$

$^1H$ NMR (400 MHz, DMSO-$d_6$) $\delta$ = 9.66 (s, 1H), 8.70 (s, 1H), 7.82 (s, 1H), 7.75 - 7.66 (m, 2H), 7.09 - 6.97 (m, 2H), 6.92 - 6.81 (m, 1H), 6.70 - 6.58 (m, 1H), 6.33 (br d, $J$ = 8.0 Hz, 1H), 5.06 - 4.45 (m, 1H), 3.62 (m, 5H), 3.40 - 3.25 (m, 5H), 2.88 (s, 3H), 2.50 - 2.45 (m, 4H), 2.45 - 1.89 (m, 5H), 1.04 (br s, 3H)

**Synthesis of ZNL-4**

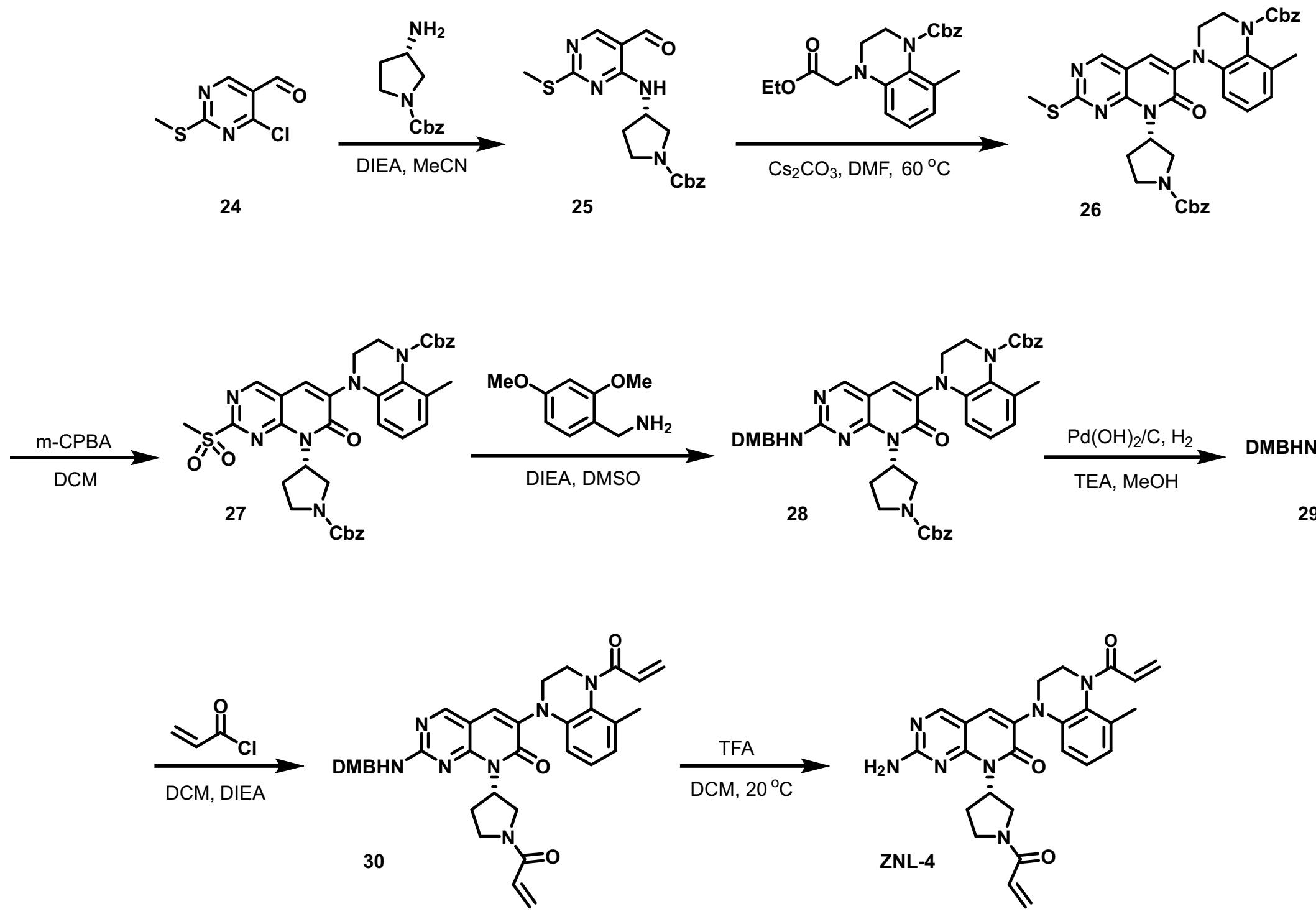


**Compound 25:**

To a solution of 4-chloro-2-methylsulfanyl-pyrimidine-5-carbaldehyde **24** (1.56 g, 8.27 mmol, 1.1 *eq*) in ACN (100 mL) was added DIEA (2.92 g, 22.55 mmol, 3.93 mL, 3 *eq*) and benzyl (3*S*)-3-aminopyrrolidine-1-carboxylate (1.66 g, 7.52 mmol, 1 *eq*). The mixture was stirred at 20°C for 1 hr. The reaction mixture was diluted with $H_2O$ 100 ml and then extracted with DCM (50 mL * 4). The combined organic layers were dried over $Na_2SO_4$, filtered and concentrated under reduced pressure to give a residue. The residue was purified by flash silica gel chromatography (ISCO®; 20 g SepaFlash® Silica Flash Column, Eluent of 0 ~ 20% Ethyl acetate/ Petroleum ether gradient @ 80 mL/ min). Compound **25** (2 g, 5.26 mmol, 70.00% yield, 98.00% purity) was obtained as a yellow solid.

**Compound 26:**

To a solution of **25** (1 g, 2.68 mmol, 1 *eq*) in DMF (20 mL) was added $Cs_2CO_3$ (2.62 g, 8.05 mmol, 3 *eq*) and benzyl 4-(2-ethoxy-2-oxo-ethyl)-8-methyl-2,3-dihydroquinoxaline-1-carboxylate (989.22 mg, 2.68 mmol, 1 *eq*). The mixture was stirred at 60°C for 2 hr. LCMS showed benzyl (S)-3-((5-formyl-2-(methylthio) pyrimidin-4-yl) amino) pyrrolidine-1-carboxylate (1 g, 2.68 mmol, 1 *eq*) was consumed completely and 14% of desired compound was detected. The reaction mixture was diluted with $H_2O$ 100 ml and then extracted with DCM (50 mL * 4). The combined organic layers were dried over $Na_2SO_4$, filtered and concentrated under reduced pressure to give a residue. The residue was purified by flash silica gel chromatography (ISCO®; 40g SepaFlash® Silica Flash Column, Eluent of 0 ~ 30% Ethyl acetate/ Petroleum ether gradient @ 80 mL/ min). Compound **26** (1.3 g, 1.61 mmol, 60.09% yield, 84% purity) was obtained as a yellow solid.

**Compound 27:**

To a solution of **26** (1.2 g, 1.77 mmol, 1 *eq*) in DCM (3 mL) was added *m*-CPBA (539.97 mg, 2.66 mmol, 85% purity, 1.5 *eq*). The mixture was stirred at 20°C for 12 hr. The reaction mixture was diluted with $Na_2SO_3$ saturated 100 ml and then extracted with DCM (50 mL *4). The combined organic layers were dried over $Na_2SO_4$, filtered and concentrated under reduced pressure to give a residue. Compound **27** (1.3 g, crude) was obtained as a yellow oil.

**Compound 28:**

To a solution of **27** (650 mg, 917.07 μmol, 1 *eq*) in Tol. (0.5 mL) was added DIEA (474.10 mg, 3.67 mmol, 638.95 μL, 4 *eq*) and (2,4-dimethoxyphenyl) methanamine (168.67 mg, 1.01 mmol, 151.55 μL, 1.1 *eq*). The mixture was stirred at 20°C for 1 hr. The reaction mixture was diluted with $H_2O$ 100ml and then extracted with DCM (50 mL * 4). The combined organic layers were dried over $Na_2SO_4$, filtered and concentrated under reduced pressure to give a residue. The residue was purified by flash silica gel chromatography (ISCO®; 20 g SepaFlash® Silica Flash Column, Eluent of 0 ~ 20% Petroleum ether: Ethyl acetate @ 80 mL/ min). Compound **28** (550 mg, 674.47 μmol, 73.55% yield, 97.6% purity) was obtained as a yellow solid.

**Compound 29:**

To a solution of **28** (470 mg, 590.54 μmol, 1 *eq*) in MeOH (25 mL) and THF (2 mL) was added $Pd(OH)_2$ (0.400 g, 590.54 μmol, 10% purity, 1.00 *eq*) and TEA (59.76 mg, 590.54 μmol, 82.20 μL, 1 *eq*). The mixture was stirred at 20 °C for 1 hr under $H_2$. The mixture was filtered through a Celite pad, and the filtrate was concentrated to give a residue. Compound **29** (330 mg, crude) was obtained as a yellow solid.

**Compound 30:**

To a solution of **29** (150 mg, 284.30 μmol, 1 *eq*) in DCM (5 mL) was added TEA (86.30 mg, 852.89 μmol, 118.71 μL, 3 *eq*) and prop-2-enoyl chloride (46.32 mg, 511.74 μmol, 41.58 μL, 1.8 *eq*) at 0°C. The mixture was stirred at 20°C for 1 hr. The reaction mixture was concentrated under reduced pressure to give a residue. Compound **30** (150 mg, crude) was obtained as a yellow solid.

**ZNL-4:**

To a solution of **30** (150 mg, 235.96 μmol, 1 *eq*) in DCM (5 mL) was added TFA (807.11 mg, 7.08 mmol, 525.81 μL, 30 *eq*). The mixture was stirred at 12°C for 12 hr. The mixture was filtered through a Celite pad, and the filtrate was concentrated to give a residue. The residue was purified by prep-HPLC (column: Phenomenex Luna C18 75 * 30 mm * 3 um; mobile phase: [$H_2O$ (0.1% TFA)-ACN]; gradient: 15% - 40% B over 8.0 min). **ZNL-4** (62.00 mg, 98.68 μmol, 41.82% yield, 95.43% purity, TFA) was obtained as a yellow solid. MS (ESI): *m*/*z* = 486.4 $[M+H]^+$

$^1H$ NMR (400 MHz, DMSO-$d_6$) $\delta$ = 8.59 (s, 1H), 7.85 (br s, 1H), 7.28 (br s, 2H), 7.05 - 6.76 (m, 1H), 6.70 - 6.46 (m, 2H), 6.34 (br d, *J* = 8.0 Hz, 1H), 6.28 (br s, 1H), 6.26 (br d, *J* = 2.5 Hz, 1H), 6.17 - 6.10 (m, 1H),

5.88 - 5.55 (m, 2H), 4.86 (br dd, $J$ = 3.8, 12.3 Hz, 1H), 4.13 - 3.91 (m, 1H), 3.89 - 3.59 (m, 4H), 3.49 (br s, 1H), 3.05 - 2.90 (m, 1H), 2.80 - 2.62 (m, 1H), 2.27 - 1.90 (m, 5H)

**Synthesis of ZNL-5**

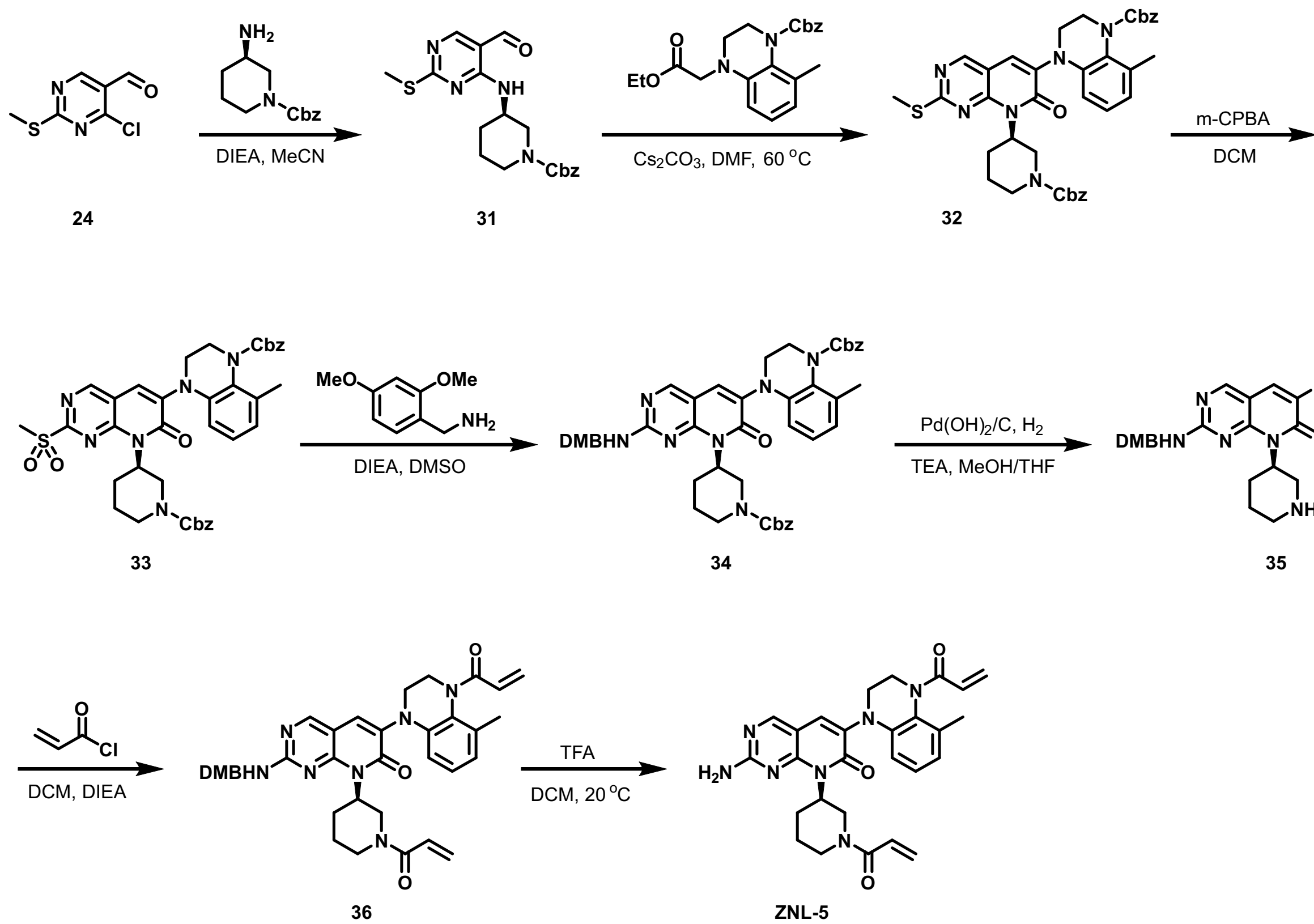


**Compound 31:**

To a solution of 4-chloro-2-methylsulfanyl-pyrimidine-5-carbaldehyde **24** (5.40 g, 28.64 mmol, 1 *eq*) in MeCN (75 mL) was added DIEA (11.10 g, 85.91 mmol, 14.96 mL, 3 *eq*) and benzyl (3*R*)-3-aminopiperidine-1-carboxylate (6.37 g, 27.20 mmol, 0.95 *eq*) at 20°C. The mixture was stirred at 20°C for 4 hr. The residue was purified by column chromatography ($SiO_2$, Petroleum ether/ Ethyl acetate = 1/0 to 1/2, TLC (Petroleum ether: Ethyl acetate = 1/2, Rf = 0.55). Compound **31** (5 g, 12.94 mmol, 45.18% yield) was obtained as a yellow oil.

**Compound 32:**

To a solution of **31** (1 g, 2.59 mmol, 1 *eq*) in DMF (25 mL) was added $Cs_2CO_3$ (2.53 g, 7.76 mmol, 3 *eq*) and benzyl 4-(2-ethoxy-2-oxo-ethyl)-8-methyl-2,3-dihydroquinoxaline-1-carboxylate (953.32 mg, 2.59 mmol, 1 *eq*). The mixture was stirred at 60°C for 2 hr. The reaction mixture was filtered and the filtrate concentrated under reduced pressure to give a residue. The residue was purified by column chromatography ($SiO_2$, Petroleum ether/ Ethyl acetate = 1/0 to 1/1, TLC: Petroleum ether/Ethyl acetate = 1/1, Rf = 0.42). Compound **32** (1.1 g, 1.59 mmol, 61.54% yield) was obtained as a yellow solid.

**Compound 33:**

To a solution of **32** (1.1 g, 1.59 mmol, 1 *eq*) in DCM (15 mL) was added *m*-CPBA (858.70 mg, 3.98 mmol, 80% purity, 2.5 *eq*) at 0°C. The mixture was stirred at 25°C for 2 hr. The reaction mixture was quenched by addition *sat.aq.* $Na_2SO_3$ 10 mL at 0°C and then extracted with DCM 20 mL (10 mL * 2). The combined organic layers were washed with brine 20 mL (5 mL * 4), dried over $Na_2SO_4$, filtered and concentrated under reduced pressure to give a yellow solid. Compound **33** (1.0 g, 1.38 mmol, 86.88% yield) was obtained as a yellow solid.

**Compound 34:**

To a solution of **33** (500 mg, 691.75 µmol, 1 *eq*) in DMSO (5 mL) was added DIEA (268.20 mg, 2.08 mmol, 361.46 µL, 3 *eq*) and (2,4-dimethoxyphenyl)methaneamine (231.33 mg, 1.38 mmol, 207.84 µL, 2 *eq*). The reaction mixture was concentrated under reduced pressure to give a residue. The residue was purified by column chromatography ($SiO_2$, Petroleum ether/ Ethyl acetate = 1/0 to 1/1). Compound **34** (350 mg, 411.41 µmol, 59.47% yield, 95.2% purity) was obtained as a yellow solid.

**Compound 35:**

To a solution of **34** (350 mg, 432.15 µmol, 1 *eq*) in MeOH (3 mL) and THF (0.5 mL) was added TEA (43.73 mg, 432.15 µmol, 60.15 µL, 1 *eq*) and $Pd(OH)_2/C$ (100 mg, 432.15 µmol, 1.00 *eq*) under $N_2$ atmosphere. The suspension was degassed and purged with $H_2$ for 3 times. The mixture was stirred under H2 (15 Psi) at 25°C for 2 hr. The reaction mixture was filtered, and the filtrate was concentrated under reduced pressure to give a yellow solid. Compound **35** (180 mg, 332.32 µmol, 76.90% yield) was obtained as a yellow solid.

**Compound 36:**

To a solution **36** (130.00 mg, 240.01 µmol, 1 *eq*) in DCM (2 mL) was added TEA (72.86 mg, 720.03 µmol, 100.22 µL, 3 *eq*) and prop-2-enoyl chloride (43.45 mg, 480.02 µmol, 39.00 µL, 2 *eq*) at 0°C. The mixture was stirred at 25 °C for 2 hr. Compound **36** (250 mg, crude) was obtained as a yellow solid.

**ZNL-5:**

To a solution of **36** (250 mg, 404.70 µmol, 1 *eq*) in DCM (4 mL) was added TFA (1.23 g, 10.77 mmol, 0.8 mL, 26.61 *eq*). The mixture was stirred at 25 °C for 2 hr. The mixture was concentrated by vacuum under reduced pressure. The residue was purified by prep-HPLC (column: Phenomenex Luna C18 100 * 30 mm*5 um; mobile phase: [$H_2O$ (0.2% FA) - ACN]; gradient: 1%-55% B over 8.0 min). Compound **ZNL-5** (13.46 mg, 24.63 µmol, 40.85% yield, 99.85% purity) was obtained as a yellow solid. MS (ESI): *m*/*z* = 500.3 $[M+H]^+$

$^1$H NMR (400 MHz, DMSO-$d_6$) $\delta$ = 8.56 (s, 1H), 7.81 (s, 1H), 7.29 (br s, 2H), 7.02 - 6.71 (m, 2H), 6.66 - 6.49 (m, 1H), 6.40 - 6.18 (m, 3H), 6.11 (br d, J=16.63 Hz, 1H), 5.86 - 5.58 (m, 2H), 4.86 (m, 1H), 4.55 - 4.32 (m, 1H), 4.15 - 3.98 (m, 1H), 3.65 (br d, *J* = 6.25 Hz, 1H), 3.49 - 3.40 (m, 2H), 3.31 - 3.21 (m, 2H), 3.04 - 2.92 (m, 1H), 2.17 - 1.96 (m, 3H), 1.90 - 1.66 (m, 2H), 1.55 - 1.19 (m, 2H)

**Synthesis of ZNL-6**

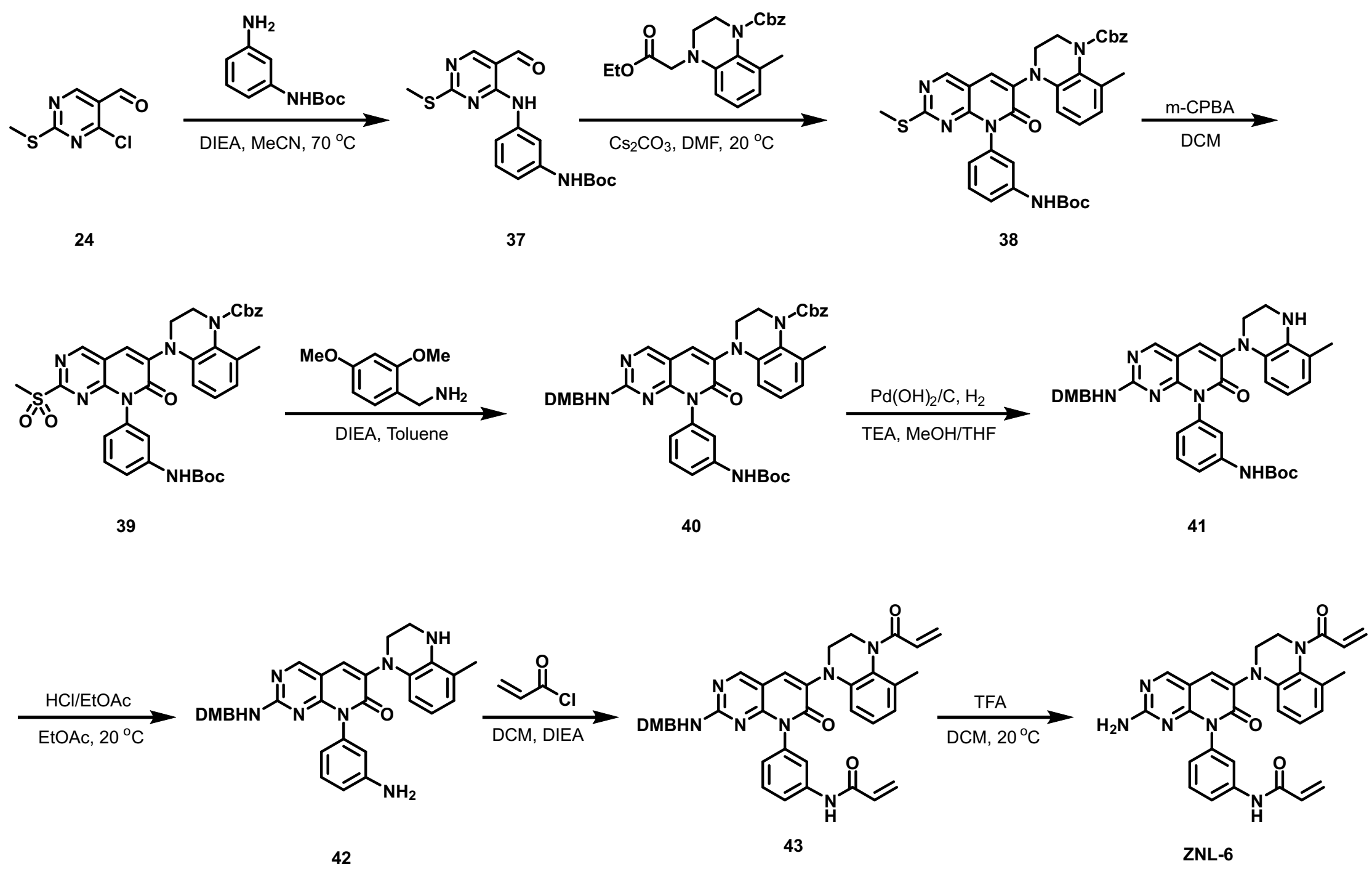


**Compound 37:**

To a solution of 4-chloro-2-(methylthio) pyrimidine-5-carbaldehyde **24** (3 g, 15.90 mmol, 1 *eq*) and tert-butyl N-(3-aminophenyl) carbamate (3.31 g, 15.90 mmol, 1 *eq*) in MeCN (30 mL) was added DIEA (4.11 g, 31.81 mmol, 5.54 mL, 2 *eq*). The mixture was stirred at 70°C for 12 hr. The reaction mixture was concentrated under reduced pressure to remove solvent. The residue was diluted with $H_2O$ 30 mL and extracted with EtOAc (30 mL * 3). The combined organic layers were dried over $Na_2SO_4$, filtered and concentrated under reduced pressure to give a residue. The residue was purified by flash silica gel chromatography (ISCO®; 12 g SepaFlash® Silica Flash Column, Eluent of 0 ~ 30% Ethyl acetate/Petroleum ethergradient @ 80 mL/min). Compound **37** (4.3 g, 11.93 mmol, 75.01% yield) was obtained as a yellow oil.

**Compound 38:**

To a solution of 37 (500 mg, 1.39 mmol, 1 eq) in DMF (20 mL) was added $Cs_2CO_3$ (1.36 g, 4.16 mmol, 3 eq) and benzyl 4-(2-ethoxy-2-oxoethyl)-8-methyl-3,4-dihydroquinoxaline-1(2H)-carboxylate (511.09 mg, 1.39 mmol, 1 eq). The mixture was stirred at 20 °C for 2 hr. The reaction mixture was diluted with $H_2O$ 100 ml and then extracted with DCM (50 mL * 4). The combined organic layers were dried over $Na_2SO_4$, filtered and concentrated under reduced pressure to give a residue. The residue was purified by flash silica gel chromatography (ISCO®; 40g SepaFlash® Silica Flash Column, Eluent of 0~20% Ethyl acetate/Petroleum ethergradient @ 80 mL/min). Compound **38** (370 mg, 500.92 μmol, 36.11% yield, 90% purity) was obtained as a white solid.

**Compound 39:**

To a solution of **38** (370 mg, 556.58 μmol, 1 *eq*) in DCM (10 mL) was added *m*-CPBA (169.50 mg, 834.87 μmol, 85% purity, 1.5 *eq*). The mixture was stirred at 20 °C for 12 hr. The reaction mixture was diluted with $Na_2SO_3$ saturated 20 ml and then extracted with DCM (10 mL *4). The combined organic layers were dried over $Na_2SO_4$, filtered and concentrated under reduced pressure to give a residue. Compound 39 (380 mg, crude) was obtained as a yellow solid.

**Compound 40:**

To a solution of **39** (360 mg, 516.67 μmol, 1 eq) in Tol. (3 mL) was added DIEA (267.10 mg, 2.07 mmol, 359.97 μL, 4.0 *eq*), (2,4-dimethoxyphenyl) methanamine (95.03 mg, 568.34 μmol, 85.38 μL, 1.1 *eq*). The mixture was stirred at 20°C for 1 hr. The reaction mixture was diluted with $H_2O$ 100 ml and then extracted with DCM (50 mL * 4). The combined organic layers were dried over $Na_2SO_4$, filtered and concentrated under reduced pressure to give a residue. The residue was purified by flash silica gel chromatography (ISCO®; 20 g SepaFlash® Silica Flash Column, Eluent of 0~20% Petroleum ether:Ethyl acetate @ 80 mL/min). Compound **40** (400 mg, 474.57 μmol, 91.85% yield, 93% purity) was obtained as a white solid.

**Compound 41:**

To a solution of **40** (400 mg, 510.29 μmol, 1 *eq*) in MeOH (2.5 mL) and THF (0.4 mL) was added $Pd(OH)_2/C$ (0.400 g, 510.29 μmol, 10% purity, 1.00 eq) and TEA (51.64 mg, 510.29 μmol, 71.03 μL, 1 eq). The mixture was stirred at 20°C for 1 hr under $H_2$ (15 Psi). The mixture was filtered through a Celite pad, and the filtrate was concentrated to give a residue. Compound **41** (330 mg, crude) was obtained as a yellow solid.

**Compound 42:**

To a solution of **41** (150 mg, 230.86 μmol, 1 eq) in EtOAc (2 mL) was added HCl/EtOAc (4 M, 2 mL, 34.65 eq). The mixture was stirred at 20°C for 2 hr. The reaction mixture was concentrated under reduced pressure to give a residue. Compound **41** (130 mg, crude, HCl) was obtained as a yellow solid.

**Compound 43:**

To a solution of **42** (130 mg, 236.53 μmol, 1 eq) in DCM (5 mL) was added TEA (119.67 mg, 1.18 mmol, 164.61 μL, 5 eq) and prop-2-enoyl chloride (38.53 mg, 425.75 μmol, 34.59 μL, 1.8 eq) at 0°C. The mixture was stirred at 20°C for 1 hr. The mixture was concentrated by vacuum under reduced pressure. Compound **43** (155.57 mg, crude) was obtained as a yellow solid.

**ZNL-6:**

To a solution of **43** (130 mg, 197.65 μmol, 1 eq) in DCM (2.5 mL) was added TFA (901.46 mg, 7.91 mmol, 587.27 μL, 40 *eq*). The mixture was stirred at 20 °C for 12 hr. The mixture was concentrated by vacuum under reduced pressure. The mixture was concentrated by vacuum under reduced pressure. The residue was purified by prep-HPLC (column: Phenomenex Luna C18 100*30 mm*5 um;mobile phase: [$H_2O$ (0.2%

FA) - ACN]; gradient:1%-55% B over 8.0 min). Compound **ZNL-6** (8.60 mg, 15.47 μmol, 22.89% yield, 99.55% purity) was obtained as a yellow solid. MS (ESI): *m/z* = 508.3 [M+H] $^+$

$^1$H NMR (400 MHz, DMSO-$d_6$) $\delta$ = 10.30 (br s, 1 H), 8.62 (br s, 1H), 7.95 (br s, 1H), 7.76 - 7.62 (m, 1H), 7.54 (br s, 1H), 7.44 (br d, *J* = 6.8 Hz, 1H), 7.21 - 6.83 (m, 4H), 6.63 - 6.35 (m, 3H), 6.33 - 6.12 (m, 3H), 5.85 - 5.60 (m, 2H), 5.00 - 4.72 (m, 1H), 3.77 - 3.62 (m, 1H), 3.19 - 3.16 (m, 1H), 2.98-2.95 (m, 1H), 2.14 - 1.98 (m, 3H).

**Synthesis of ZNL-7**

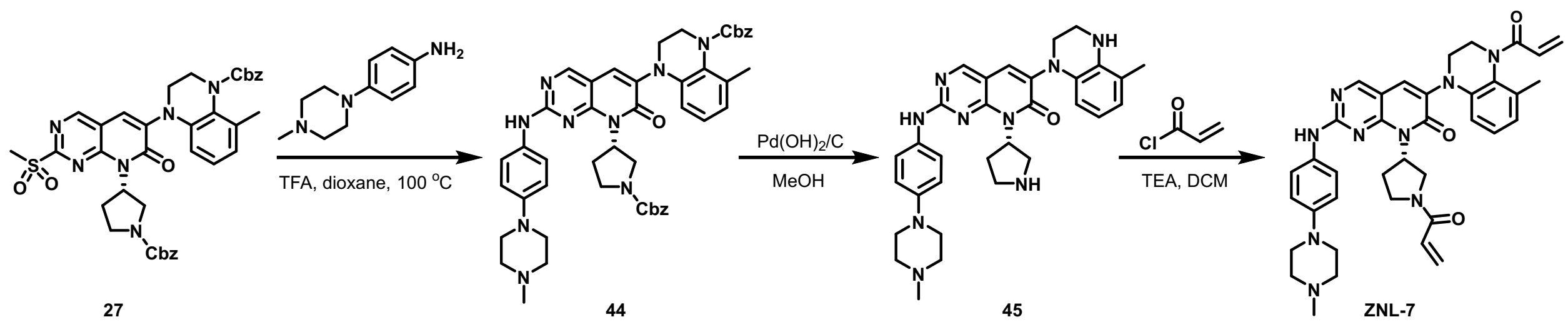


**Compound 44:**

To a solution of **27** (650 mg, 917.07 μmol, 1 eq) in dioxane (0.5 mL) was added TFA (156.85 mg, 1.38 mmol, 102.18 μL, 1.5 eq) 4-(4-methylpiperazin-1-yl) aniline (192.95 mg, 1.01 mmol, 1.1 eq). The mixture was stirred at 120°C for 1 hr. The reaction mixture was concentrated under reduced pressure to give a residue. The residue was purified by prep-HPLC (column: Phenomenex luna C18 100 * 40 mm *5 um; mobile phase: [$H_2O$ (0.1% TFA)-ACN]; gradient: 40% - 70% B over 8.0 min). Compound **44** (400 mg, 427.04 μmol, 46.57% yield, 99.71% purity, TFA) was obtained as a yellow solid.

**Compound 45:**

To a solution of **44** (200 mg, 243.92 μmol, 1 *eq*) in MeOH (25 mL) and THF (2 mL) was added $Pd(OH)_2$ (0.200 g, 10% purity) and TEA (24.68 mg, 243.92 μmol, 33.95 μL, 1 *eq*). The mixture was stirred at 20°C for 1 hr under $H_2$. The mixture was filtered through a Celite pad, and the filtrate was concentrated to give a residue. Compound **45** (130 mg, crude) was obtained as a yellow solid.

**ZNL-7:**

To a solution of **45** (120 mg, 217.52 μmol, 1 *eq*) in DCM (5 mL) was added TEA (66.03 mg, 652.55 μmol, 90.83 μL, 3 *eq*) and prop-2-enoyl chloride (35.44 mg, 391.53 μmol, 31.81 μL, 1.8 eq) at 0°C. The mixture was stirred at 20°C for 1 hr. The mixture was filtered through a Celite pad, and the filtrate was concentrated to give a residue. The residue was purified by prep-HPLC (column: Phenomenex Luna C18 75 * 30 mm* 3 um; mobile phase: [$H_2O$ (0.1% TFA) - ACN]; gradient:15% - 40% B over 8.0 min). Compound **ZNL-7** (88.11 mg, 113.10 μmol, 52.00% yield, 99.33% purity, TFA) was obtained as a white solid. MS (ESI): *m/z* = 660.5 [M+H] $^+$

$^1$H NMR (400 MHz, DMSO-D$_6$) $\delta$ = 10.00 - 9.77 (m, 1H), 8.72 (d, J = 2.1 Hz, 1H), 7.90 (br d, J = 3.5 Hz, 1H), 7.50 (br t, J = 8.5 Hz, 2H), 6.96 - 6.89 (m, 1H), 6.88 - 6.81 (m, 2H), 6.72 - 6.62 (m, 1H), 6.59 - 6.44 (m, 1H), 6.42 - 6.31 (m, 1H), 6.29 - 6.26 (m, 1H), 6.21 - 6.07 (m, 2H), 5.79 - 5.57 (m, 2H), 4.98 - 4.78 (m, 1H), 4.32 - 3.93 (m, 1H), 3.90 - 3.79 (m, 1H), 3.76 - 3.64 (m, 2H), 3.13 - 2.75 (m, 8H), 2.45 (br s, 4H), 2.23 (s, 3H), 2.18 - 1.98 (m, 5H)

**Synthesis of ZNL-8**

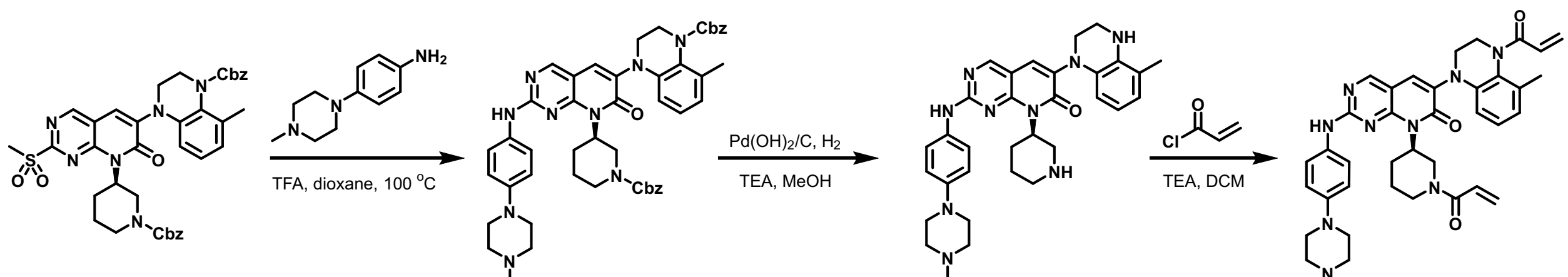


**Compound 46:**

To a solution of **33** (500 mg, 691.75 µmol, 1 *eq*) and 4-(4-methylpiperazin-1-yl) aniline (132.31 mg, 691.75 µmol, 1 *eq*) in dioxane (5 mL) was added TFA (230.25 mg, 2.02 mmol, 0.15 mL, 2.92 *eq*). The mixture was stirred at 120°C for 12 hr. The mixture was concentrated by vacuum under reduced pressure. The residue was purified by prep-HPLC (column: Phenomenex Luna C18 100 * 30 mm * 5 um; mobile phase: [$H_2O$ (0.2% FA) - ACN]; gradient: 40% - 70% B over 8.0 min). Compound **46** (400 mg, 479.63 µmol, 69.34% yield) was obtained as a yellow solid.

**Compound 47:**

A mixture of $Pd(OH)_2$ (200 mg, 1.42 mmol, 3.96 *eq*) in MeOH (1.5 mL) and THF (1.5 mL) was added to **46** (300 mg, 359.72 µmol, 1 *eq*) and TEA (218.10 mg, 2.16 mmol, 300.00 µL, 5.99 *eq*) was degassed and purged with $H_2$ for 3 times, then the mixture was stirred at 25°C for 2 hr under $H_2$ (15 Psi) atmosphere. The reaction mixture was filtered, and the filtrate was concentrated to give a residue. Compound **47** (150 mg, crude) was obtained as a yellow oil.

**ZNL-8:**

To a solution of **47** (150 mg, 265.15 µmol, 1 *eq*) in DCM (2 mL) was added TEA (80.49 mg, 795.46 µmol, 110.72 µL, 3 *eq*) and prop-2-enoyl chloride (48.00 mg, 530.31 µmol, 43.09 µL, 2 *eq*) at 0°C. The mixture was stirred at 25°C for 2 hr. The mixture was concentrated by vacuum under reduced pressure. The residue was purified by prep-HPLC (column: Phenomenex Luna C18 100 * 30 mm* 5um; mobile phase: [$H_2O$ (0.2% FA)-ACN]; gradient: 1% - 55% B over 8.0 min). Compound **ZNL-8** (39.57 mg, 54.52 µmol, 20.56% yield, 99.17% purity) was obtained as a yellow solid. MS (ESI): $m/z$ = 674.5 $[M+H]^+$

$^1$H NMR (400 MHz, DMSO-$d_6$) $\delta$ = 10.01 - 9.49 (m, 1H), 8.71 (s, 1H), 7.88 (s, 1H), 7.63 - 7.22 (m, 2H), 7.03 - 6.72 (m, 4H), 6.65 (br d, *J* = 7.1 Hz, 1H), 6.41 - 6.03 (m, 4H), 5.84 - 5.71 (m, 1H), 5.64 - 5.31 (m, 1H), 5.00 - 4.74 (m, 1H), 4.67 - 4.34 (m, 1H), 4.23 - 4.03 (m, 1H), 3.88 - 3.62 (m, 2H), 3.17 - 2.97 (m, 6H), 2.79

- 2.53 (m, 2H), 2.46 (br d, *J* = 3.4 Hz, 4H), 2.23 (s, 3H), 2.17 - 2.02 (m, 3H), 1.93 - 1.62 (m, 2H), 1.49 - 1.10 (m, 2H)

**Synthesis of YNW-1**

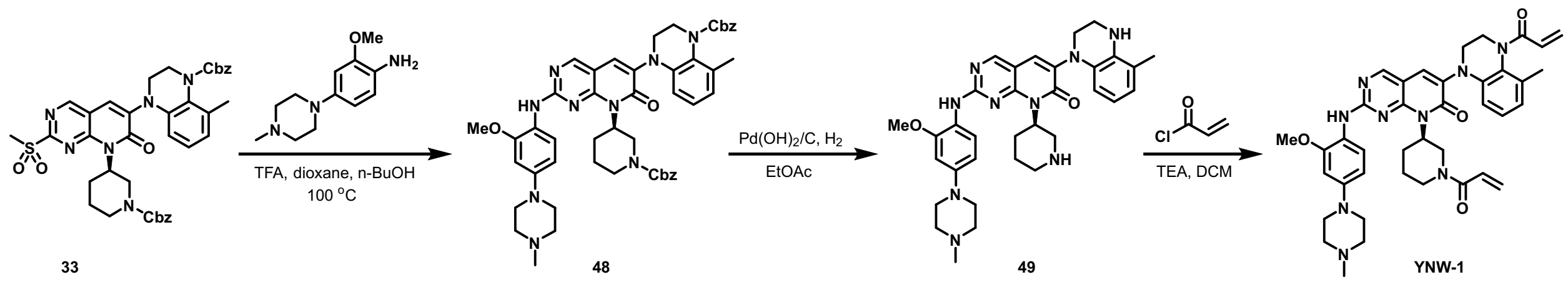

**Compound 48:**

To a solution of benzyl 33 (2 g, 2.8 mmol, 1 eq) in dioxane (16 mL) and n-BuOH (32 mL) was added 2-methoxy-4-(4-methylpiperazin-1-yl) aniline (1.2 g, 5.5 mmol, 2 eq) and TFA (1.9 g, 16.6 mmol, 1.2 mL, 6 eq). The mixture was stirred at 100°C for 12 hr. The reaction mixture was concentrated under reduced pressure to give a residue. The residue was purified by flash silica gel chromatography (120 g Silica Flash Column, Eluent of 0~7% Methanol/Dichloromethane gradient @ 120 mL/min) to give **48** (5.5 g, 6.4 mmol, ee%=100%) as a yellow solid.

**Compound 49:**

To a solution of $Pd(OH)_2/C$ (5 g, 20% purity) in EtOAc (50 mL) was added **48** (5 g, 5.8 mmol, 1 eq) under $N_2$ atmosphere. The suspension was degassed and purged with $H_2$ for 3 times. The mixture was stirred under $H_2$ (15 Psi) at 40°C for 12 hr. Then the reaction mixture was filtered and concentrated under reduced pressure to give a residue to give **49** (4 g, crude) as a yellow solid.

**YNW-1:**

To a solution of **49** (4 g, 6.7 mmol, 1 eq) in DCM (150 mL) was added TEA (2.0 g, 20.1 mmol, 2.8 mL, 3 eq). Then prop-2-enoyl chloride (1.2 g, 13.4 mmol, 1.1 mL, 2 eq) was added to the mixture at 0°C. The mixture was stirred at 0°C for 1 hr. The reaction mixture was concentrated under reduced pressure to remove solvent. The residue was purified by prep-HPLC (FA condition column: Phenomenex luna C18 250mm × 100mm × 15um; mobile phase: [$H_2O$ (0.02%FA)-ACN]; gradient: 10%-40% B over 28.0 min) to give **YNW-1** (852 mg, 98.63% purity) as a yellow solid. MS (ESI): m/z = 704.4 $[M+H]^+$

VT $^1$HNMR (400 MHz, DMSO-$d_6$) $\delta$ = 8.66 - 8.60 (m, 1H), 8.59 - 8.50 (m, 1H), 7.81 - 7.78 (m, 1H), 7.45 - 7.28 (m, 1H), 6.94 - 6.82 (m, 1H), 6.72 - 6.60 (m, 3H), 6.49 - 6.43 (m, 1H), 6.34 (br d, *J* = 8.1 Hz, 1H), 6.30 - 6.20 (m, 2H), 6.11 - 6.01 (m, 1H), 5.75 - 5.65 (m, 1H), 5.61 (br d, *J* = 9.4 Hz, 1H), 5.16 - 5.03 (m, 1H), 4.96 - 4.74 (m, 1H), 4.23 - 3.92 (m, 2H), 3.78 (s, 3H), 3.64 - 3.39 (m, 2H), 3.22 - 3.10 (m, 5H), 3.08 - 2.89 (m, 1H), 2.70 - 2.53 (m, 2H), 2.49 - 2.47 (m, 4H), 2.28 - 2.24 (m, 3H), 2.15 - 2.06 (m, 3H), 1.77 - 1.57 (m, 2H), 1.47 - 1.31 (m, 1H)

**Synthesis of YNW-2**

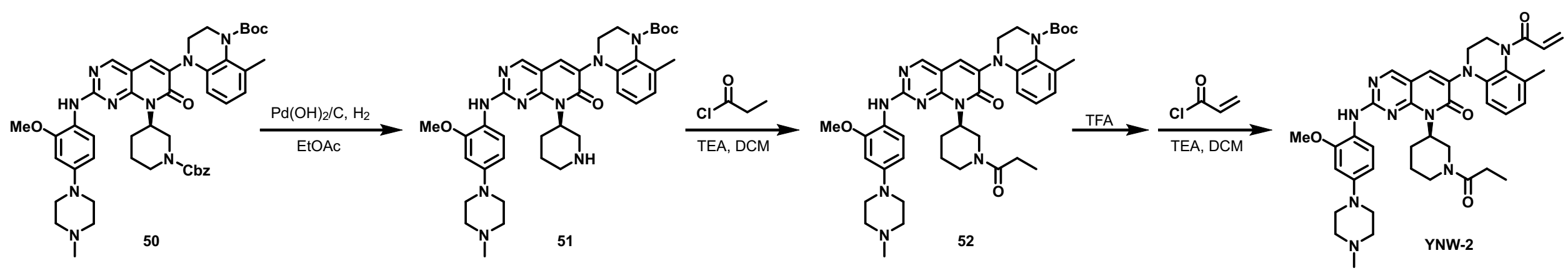

**Compound 51**:

To a solution of $Pd(OH)_2/C$ in EtOAc was added **50** (1 eq) under $N_2$ atmosphere. The suspension was degassed and purged with $H_2$ for 3 times. The mixture was stirred under $H_2$ (15 Psi) at 40 °C for 12 hr. The reaction mixture was filtered and concentrated under reduced pressure to give a residue to give **51**.

**Compound 52:**

To a solution of **51** in DCM was added TEA (3 eq). Then propionyl chloride (2 eq) was added to the mixture at 0 °C. The mixture was stirred at 0 °C for 1 hr. The reaction mixture was concentrated under reduced pressure to remove solvent. Then purified by ISCO (methanol in DCM 0-30%) to give **52**.

**YNW-2:**

To a solution of **52** in DCM was added TEA (3 eq). Then prop-2-enoyl chloride (2 eq) was added to the mixture at 0 °C. The mixture was stirred at 0 °C for 1 hr. The reaction mixture was concentrated under reduced pressure to remove solvent. Then purified by HPLC to give **YNW-2** as a yellow solid. MS (ESI): m/z = 706.1 $[M+H]^+$

$^1$H NMR (500 MHz, DMSO-$d_6$) $\delta$ (ppm) = 9.83 - 9.72 (m, 1 H), 8.65 (s, 1 H), 7.85 (s, 1 H), 6.89 (m, 1H), 6.72 (m, 1H), 6.59 (m, 3H), 6.38 - 6.18 (m, 3H), 5.72 (m, 1H), 4.91 - 4.84 (m, 1H), 3.91 - 3.63 (m, 6H), 3.56 (d, $J$ = 10.0 Hz, 2H), 3.41 (m, 1H), 3.18 (m, 2H), 2.92 (m, 5H), 2.11-2.02 (s, 6H), 1.56 - 1.28 (m, 8H), 0.93 (m, 4H).

**Synthesis of YNW-3**

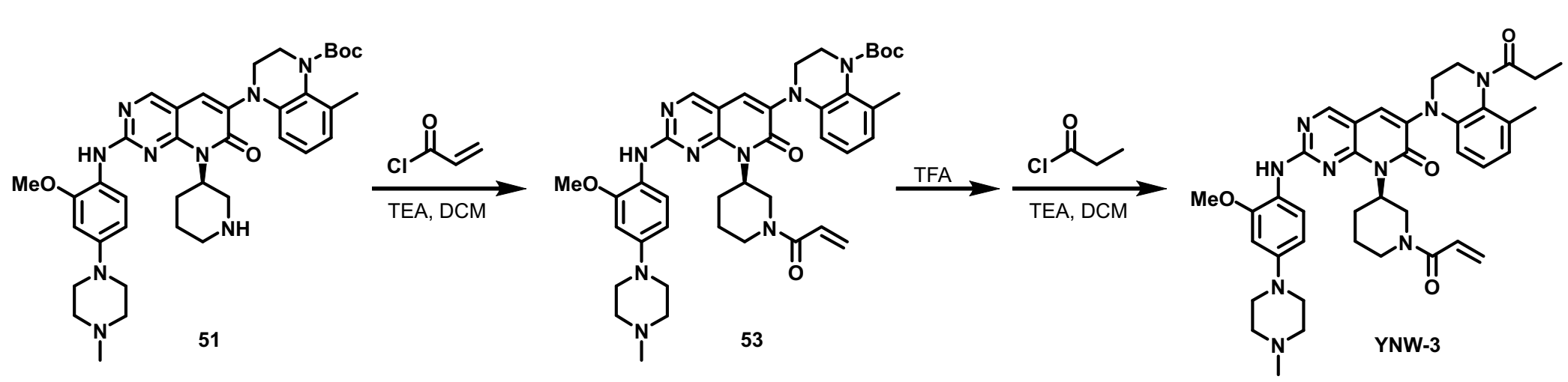

**Compound 53:**

To a solution of **51** in DCM was added TEA (3 eq). Then prop-2-enoyl chloride (2 eq) was added to the mixture at 0 °C. The mixture was stirred at 0°C for 1 hr. The reaction mixture was concentrated under reduced pressure to remove solvent. Then purified by ISCO (methanol in DCM 0-30%) to give **53**.

**YNW-3:**

To a solution of **53** in DCM was added TEA (3 eq). Then propionyl chloride (2 eq) was added to the mixture at 0 °C. The mixture was stirred at 0 °C for 1 hr. The reaction mixture was concentrated under reduced pressure to remove solvent. Then purified by HPLC to give **YNW-3** as a yellow solid. MS (ESI): m/z = 706.1 $[M+H]^+$

$^1$H NMR (500 MHz, DMSO-$d_6$) $\delta$ (ppm) = 9.75 (m, 1H), 8.67 (s, 1H), 7.86-7.80 (m, 1H), 6.89 (m, 1H), 6.74-6.65 (m, 3H), 6.53 (m, 1H), 6.37-6.25 (m, 2H), 6.06 (m, 1H), 5.70-5.57 (m, 1 H), 4.85-4.77 (m, 1H), 3.82 (m, 6H), 3.56 (m, 2H), 3.41-3.32 (m, 1H), 3.19 (m, 2H), 2.97-2.83 (m, 5H), 2.21 (s, 3H), 2.16-1.96 (m, 3H), 1.94-1.20 (m, 8H), 1.03 (m, 4H).
